# Structure of Furan from 44 Isotopologues—Resolving Differences in the Theoretical (*r*_*e*_) and Semi-Experimental Equilibrium (*r_e_* ^SE^) Structures

**DOI:** 10.1021/acs.jpca.6c04036

**Published:** 2026-09-16

**Authors:** Kolton T. Mehalko, Brian J. Esselman, Peter R. Franke, Martin S. Holdren, Gabi Wenzel, D. Archie Stewart, Zachary P. Burke, Brett A. McGuire, R. Claude Woods, Robert J. McMahon

**Affiliations:** † Department of Chemistry, University of Wisconsin-Madison, 1101 University Avenue, Madison, Wisconsin 53706, United States; ‡ Quantum Theory Project, Departments of Physics and Chemistry, 3463 University of Florida, Gainesville, Florida 32611, United States; § Department of Chemistry, 2167 Massachusetts Institute of Technology, 77 Massachusetts Avenue, Cambridge, Massachusetts 02139, United States; ∥ Center for Astrophysics | Harvard & Smithsonian, 60 Garden Street, Cambridge, Massachusetts 02138, United States; ⊥ National Radio Astronomy Observatory, 520 Edgemont Road, Charlottesville, Virginia 22903, United States

## Abstract

The rotational spectra of furan and numerous isotopologues were recorded and analyzed between 6 and 750 GHz. High-temperature, high-pressure, base-catalyzed H/D exchange was employed to create a mixture of all possible furan-*d*_*x*_ isotopologues, allowing for the measurement and analysis of the rotational spectra of 44 furan isotopologues. The experimental rotational constants of these isotopologues, combined with CCSD­(T)/cc-pCVTZ corrections for the effects of vibration–rotation interaction and the electron-mass distribution, were used to determine a semi-experimental equilibrium (*r*_*e*_^SE^) structure of furan. The large number of isotopologues used, including 35 isotopologues observed for the first time, allowed for the determination of the *r*_*e*_^SE^ structure to a high level of accuracy and precision (≤0.0003 Å uncertainties in bond distances and ≤0.02° in bond angles). The current study additionally corrects for differences in principal axis rotation between the structure at which the vibration–rotation interaction constants were evaluated (CCSD­(T)/cc-pCVTZ) and the *r*_*e*_^SE^ structure. The difference in rotation between these two structures had been previously reported, but not addressed. The “best theoretical estimate” (BTE) for the structure of furan was determined using a CCSD­(T)/cc-pCV5Z geometry, with additional corrections addressing finite basis set, higher-level electron correlation, and relativistic effects, as well as the diagonal Born–Oppenheimer correction. The BTE structure of furan is compared to the *r*_*e*_^SE^ structure of furan, which is also compared to the previously determined *r*_*e*_^SE^ structure of its sulfur-containing analogue, thiophene.

## Graphical abstract

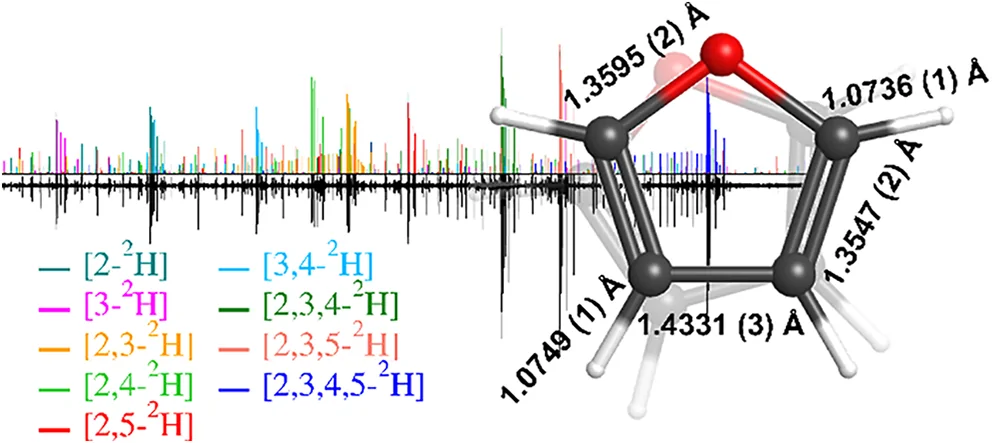

## Introduction

Furan (*c*-C_4_H_4_O, *C*_2*v*_, Figure 1) is a near-oblate (Ray’s asymmetry parameter,^1^ κ = 0.916) aromatic heterocycle with a moderate dipole moment (μ_*a*_ = 0.661 (6) D).^2^ Sirvetz reported the initial measurement of the rotational spectrum from 19 to 26 GHz in 1951,^2^ and Tomasevich et al. reported an analysis of the hydrogen hyperfine splitting of four Q-branch transitions between 2 and 5 GHz in 1973.^3^ The ground and excited vibrational states have been the focus of numerous studies involving rotational, high-resolution infrared (IR), and rotationally resolved Raman spectroscopy.^3–12^ The rotational spectrum of furan is also of interest to the field of astrochemistry, as furan has long been considered a strong candidate molecule for detection in the interstellar medium (ISM).^10,13–15^ Furan derivatives have been established to make up a more significant portion of the insoluble organic matter within the Murchison CM2 meteorite than aromatics with oxygen-containing substituents,^16^ and furan, hydrogenated derivatives of furan, and substituted furans have all recently been identified within comet 67P’s dusty coma.^17^ Despite furan’s presence in extraterrestrial bodies, the identification of many aromatic organic compounds in the ISM, and numerous dedicated astronomical searches, it and other aromatic heterocycles remain undetected in the ISM.^10,13–15,18–22^

**Figure 1:**
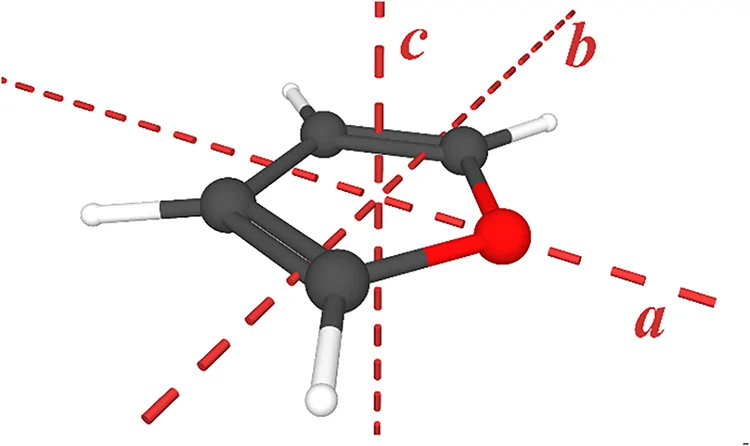
Furan (C_4_H_4_O, *C*_2*v*_, μ_*a*_ = 0.661 (6) D,^2^ κ = 0.916) with principal inertial axes.

The gas-phase structure of furan has been determined numerous times, with the first partial substitution (*r*_*s*_) structure determined by Bak et al. in 1955 from the normal isotopologue and three deuterium-substituted isotopologues.^23^ In a subsequent study, Bak et al. measured the rotational transitions of the ^13^C- and ^18^O-substituted furan isotopologues and produced the first complete *r*_*s*_ structure from the observed rotational constants (*B*_0_) from seven isotopologues.^24^ In 1978, Mata et al. reinvestigated the spectroscopy of the deuteriated furan isotopologues, including furan-*d*_4_ for the first time, and produced a new *r*_*s*_ structure from eight isotopologues that was in close agreement with the previously determined structure.^25^ In 2011, Demaison et al.^26^ produced the first semi-experimental equilibrium (*r*_*e*_^SE^) structure of furan, incorporating a ninth deuterium-substituted isotopologue from a separate study.^27^ They identified a previously unrecognized issue in *r*_*e*_^SE^ structure determinations wherein small differences between the *r*_*e*_ and *r*_*e*_^SE^ structures cause the computed vibration–rotation interaction corrections to be poorly estimated for some isotopologues, which occurred for [2-^13^C]-furan.^26^ In 2015, Penocchio et al.^28^ and Piccardo et al.^29^ determined additional *r*_*e*_^SE^ structures using nine isotopologues with vibration–rotation interaction corrections calculated using density functional theory (DFT), and produced structures that agree with the earlier work by Demaison et al. The most recent *r*_*e*_^SE^ structure of furan was determined by Özer et al., who used rotationally resolved Raman spectroscopy to reinvestigate furan and its ^13^C- and ^18^O-isotopologues. They used a statistical weighting of various rotational constants determined for each isotopologue to produce a new *r*_*e*_^SE^ structure, which is in agreement with the prior *r*_*e*_^SE^ structures.^12^

It has been shown that the precision and accuracy of *r*_*e*_^SE^ structure determinations is significantly improved by the incorporation of many isotopologues that provide additional constraints on the structural parameters, as well as use of vibration–rotation interaction and electron-mass corrections computed at the CCSD­(T)/cc-pCVTZ level of theory.^30–41^ Bond lengths and angles with 2σ uncertainties as low as ≤0.0002 Å and ≤0.02°, respectively, have been achieved when a large number of isotopologues are incorporated into the structure determination. Due to this level of precision, meaningful comparisons between *r*_*e*_^SE^ structures and high-level computed equilibrium (*r*_*e*_) structures can be made, providing insight into the accuracy of both the experimental spectroscopic constants and the computational methods being used. Several studies have now determined *r*_*e*_ structures at a high level of theory (CCSD­(T)/cc-pCV5Z or cc-pCV6Z) for comparison to *r*_*e*_^SE^ structures.^30–41^ Between studies, there are varying levels of agreement among the resultant structural parameters. Some molecular structures show complete agreement between the *r*_*e*_^SE^ structure and the “best theoretical estimate” (BTE) *r*_*e*_ structure, in the sense that all computed bond lengths and angles fall within the experimental value and uncertainty,^30,32,37^ while others deviate slightly.^31,33–36,38–41^ While the small discrepancies between the (BTE) *r*_*e*_ and the *r*_*e*_^SE^ structures could be due to poorly determined spectroscopic constants (*B*_0_) in a few limited cases, the lack of agreement is presumed to be largely due to deficiencies in the computed corrections used to obtain the equilibrium rotational constants (*B*_*e*_) or the CCSD­(T) *r*_*e*_ structure.^41^ The present study revisits the structure of furan to implement two substantive improvements. First, the current *r*_*e*_^SE^ structure is determined using a much larger number of isotopologues (44) than earlier studies. Second, the analysis employs our recently reported method^41^ to provide insight into the discrepancies between the computed *r*_*e*_ structure (CCSD­(T)/cc-pCVTZ) and the actual *r*_*e*_^SE^ structure and to remedy the issue of differential isotopologue rotation between the structures that impacts the computed vibration–rotation interaction corrections.

## Methods

### Synthesis of Furan-***d***_***x***_ Isotopologues

Deuterium-substituted furan isotopologues, furan-*d*_*x*_, were synthesized via a base-catalyzed H/D exchange of furan in D_2_O (Scheme 1), following a procedure similar to that described by Heinrich et al.^42^ Furan, D_2_O, and K_2_CO_3_ were added to a stainless steel vessel, which was sealed and heated to 235 °C for 10 h, producing a mixture of all possible deuterium-substituted furan isotopologues. In many instances, the corresponding singly substituted heavy-atom isotopologues of the various deuterium-containing species could also be detected at natural abundance. Synthetic details are provided in the Supporting Information.

**Scheme 1:**
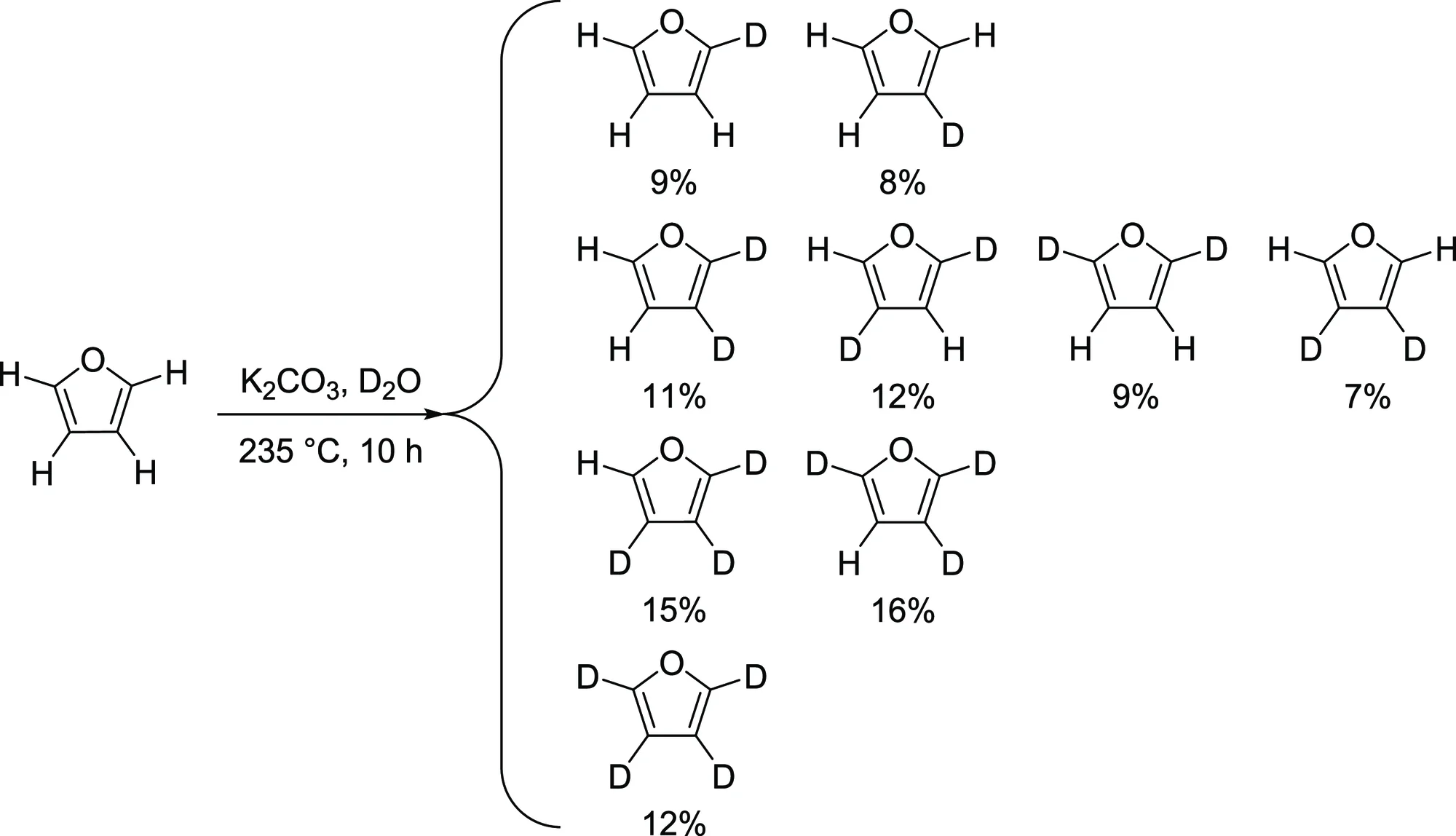
Deuteriation of Furan by Carbonate-Mediated Exchange with D_2_O under High-Temperature, High-Pressure Conditions^a^

a The approximate percentages in the scheme are estimated from relative intensities of transitions in the rotational spectrum.

### Rotational Spectroscopy

Rotational spectra of furan-*h*_4_ and furan-*d*_4_ and their corresponding heavy-atom isotopologues were obtained using commercially available samples, and the synthesized furan-*d*_*x*_ sample contained all other isotopologues. The rotational spectra were collected using one spectrometer at the University of Wisconsin-Madison and two spectrometers at the Massachusetts Institute of Technology. At the University of Wisconsin, continuous broadband spectra were obtained in the ranges of 82–125 GHz, 154–244 GHz, 235–360 GHz, 350–500 GHz, and 500–750 GHz at room temperature and a pressure of 20 mTorr, using an instrument described previously.^37,43,44^ Spectra were obtained in an automated fashion over approximately 14 days, given the following experimental parameters: 0.6 MHz/s sweep rate, 10 ms time constant, and 50 kHz AM and 500 kHz FM modulation in a tone-burst design.^45^ Measurements made using this spectrometer assume a uniform experimental uncertainty of 50 kHz. At the Massachusetts Institute of Technology, rotational spectra were measured using a previously described chirped-pulse Fourier transform microwave (CP-FTMW) spectrometer with a pulsed-jet expansion sample introduction in the range of 6.5–18 GHz.^46^ Furan was mixed with argon carrier gas to generate an ∼1% mixture by transferring ∼60 Torr of furan into a previously evacuated gas cylinder held under vacuum. Argon was added to this cylinder up to ∼6000 Torr. This mixture was pulsed through a 1 mm pinhole with a repetition rate of 3 Hz using a standard Parker Series 9 valve held open for 350 μs into a vacuum chamber held at a base pressure of ∼10^–6^ Torr, generating a supersonic expansion and rotationally cooling the analytes to ∼2 K. After a 600 μs delay, ten amplified chirps spanning 10–18 GHz for the parent species, or 6.5–18 GHz for the furan-*d*_*x*_ sample, with a chirp duration of 2 μs and separated by 30 μs were used to polarize the sample. The molecular free induction decay (FID) emission was then amplified, recorded, and digitized at 80 GS/s for 25 μs after each chirp. This process was repeated for a total of 2 million FIDs averaged for the parent species and 1.5 million FIDs averaged for the deuteriated samples. The averaged FID was filtered with a Hann window function and zero padded before being fast Fourier transformed into the frequency domain for analysis. Each sample was also measured at room temperature using a commercial BrightSpec nanoMRR (microwave rotational resonance) spectrometer operating from 18–26 GHz. Each sample was measured by injecting ∼4 mL into a previously evacuated sample holder attached to the flow cell of the instrument. Needle valves were used to flow the sample through the cell, held at a stable 4 mTorr. Ten million FIDs were collected and averaged for the parent species and 5 million FIDs were recorded and averaged for the deuteriated samples, where chirps spanning 18 MHz were used in steps across the total bandwidth to obtain the final averaged spectrum. Each 18-MHz step was acquired at a rate of ∼50 kHz, taking ∼200 s for 10 million acquisitions per step. Measurements made using these spectrometers assume a uniform experimental uncertainty of 10 kHz.

Kisiel’s Assignment and Analysis of Broadband Spectra (AABS) software package^47,48^ was used to visualize and measure rotational transitions and combine spectral segments into a continuous spectrum. SPFIT/SPCAT^49^ were used for the least-squares fitting of the rotational constants and spectral predictions, and PIFORM, PLANM, and AC programs^50^ were used for analysis. All previously reported measured transitions that were outside of the collected frequency regions were incorporated into the data set using the uncertainty reported in the original publication. All least-squares fitting files are available in the Supporting Information.

## Computational Methods

Computations were done using a development version of CFOUR^51^ using the CCSD­(T) level of theory and are similar to the calculations used in several recent *r*_*e*_^SE^ structure determinations.^30–41^ Calculations using cc-pVDZ or cc-pVTZ basis sets^52,53^ were performed with the frozen-core (fc) approximation. Calculations using cc-pCVTZ, cc-pCVQZ, and cc-pCV5Z basis sets^54^ included correlation of all electrons (ae). The CCSD­(T)/cc-pCV5Z optimized geometry was used as the basis of the BTE structure. Additionally, anharmonic second-order vibrational perturbation theory (VPT2) and magnetic calculations were carried out for the CCSD­(T)/cc-pCVTZ optimized structure at the same level of theory, providing vibration–rotation interaction corrections, centrifugal distortion constants, and electron-mass corrections for each isotopologue. Geometry optimization and anharmonic vibrational frequency calculations were additionally completed for the normal isotopologue of furan using Gaussian 16 ^55^ with the WebMO^56^ interface at the B3LYP/6–311+G­(2d,p) level, using the “verytight” convergence criteria and an “ultrafine” integration grid. As noted in a previous report,^57^ the output files of the Gaussian calculation are mislabeled as being in the I^*l*^ representation, but the constants are actually calculated in the III^*l*^ representation. Details of the BTE calculations, all computational output files, and isotopologue numbering scheme (Table S2) are available in the Supporting Information.

Vibration–rotation interaction constants from VPT2 calculations, electron-mass corrections from rotational *g*-tensor calculations, and averaged experimental determinable rotational constants were input into the *xrefit* module of CFOUR to determine the *r*_*e*_^SE^ structure through least-squares fitting. The *xrefit* module uses eq 1 ^58,59^ to determine the corrected moments of inertia for each isotopologue, where β refers to the rotational axis, *B*_*e*_ is the equilibrium rotational constant, *B*_0_″ is the determinable rotational constant,^60^ the half-sum is the vibration–rotation interaction correction, $\frac{m_{e}}{M_{p}}$ is the electron–proton mass ratio, and *g* ^ββ^ is the corresponding magnetic *g*-tensor component. The *xrefiteration* routine^32,33^ was also employed to assess the impacts on the structure of individual isotopologues and identify any isotopologues with suspect data sets. Explained in greater detail elsewhere, *xrefiteration* works by first determining an *r*_*e*_^SE^ structure from the core set of the normal and singly substituted isotopologues and subsequently adding isotopologues one-by-one in the order that results in the greatest reduction in the structural uncertainty. Multiple Z-matrices were used to obtain all structural parameters and their uncertainties.

1 $$B_{e}^{\beta }={B_{0}}^{\prime \prime \beta }+\frac{1}{2}\sum_{i}\alpha_{i}^{\beta }-\frac{m_{e}}{M_{P}}g^{\beta \beta }B^{\beta }$$

## Results and Discussion

### Analysis of Rotational Spectra

Furan is a near-oblate asymmetric top molecule (κ = 0.916) with a moderate dipole moment along its *a*-axis (μ_*a*_ = 0.661 (6) D),^2^ which gives rise to a rotational spectrum composed of doubly degenerate *a*-type transitions (^*a*^R_0,1_) at low values of *K*_*a*_. As *K*_*a*_ increases, the transitions lose degeneracy and eventually become degenerate in *K*_*c*_. Each R-branch is accompanied by a lower-intensity Q-branch consisting of two degenerate transitions (^*a*^Q_0,–1_ and ^*a*^Q_2,–1_). Due to the large value of the *C*_0_ rotational constant leading to large separation of the rotational bands, along with the relatively large energy separation between the ground state and the lowest-energy vibrationally excited state (ν_11_, 599.693 665 (26) cm^–1^),^9^ the density of spectral lines does not preclude the analysis of the ^18^O and ^13^C isotopologues at natural abundance (Figure 2). The majority of the remaining transitions observed in the spectrum have been assigned to vibrationally excited states of furan, which will be analyzed in future work.

**Figure 2:**
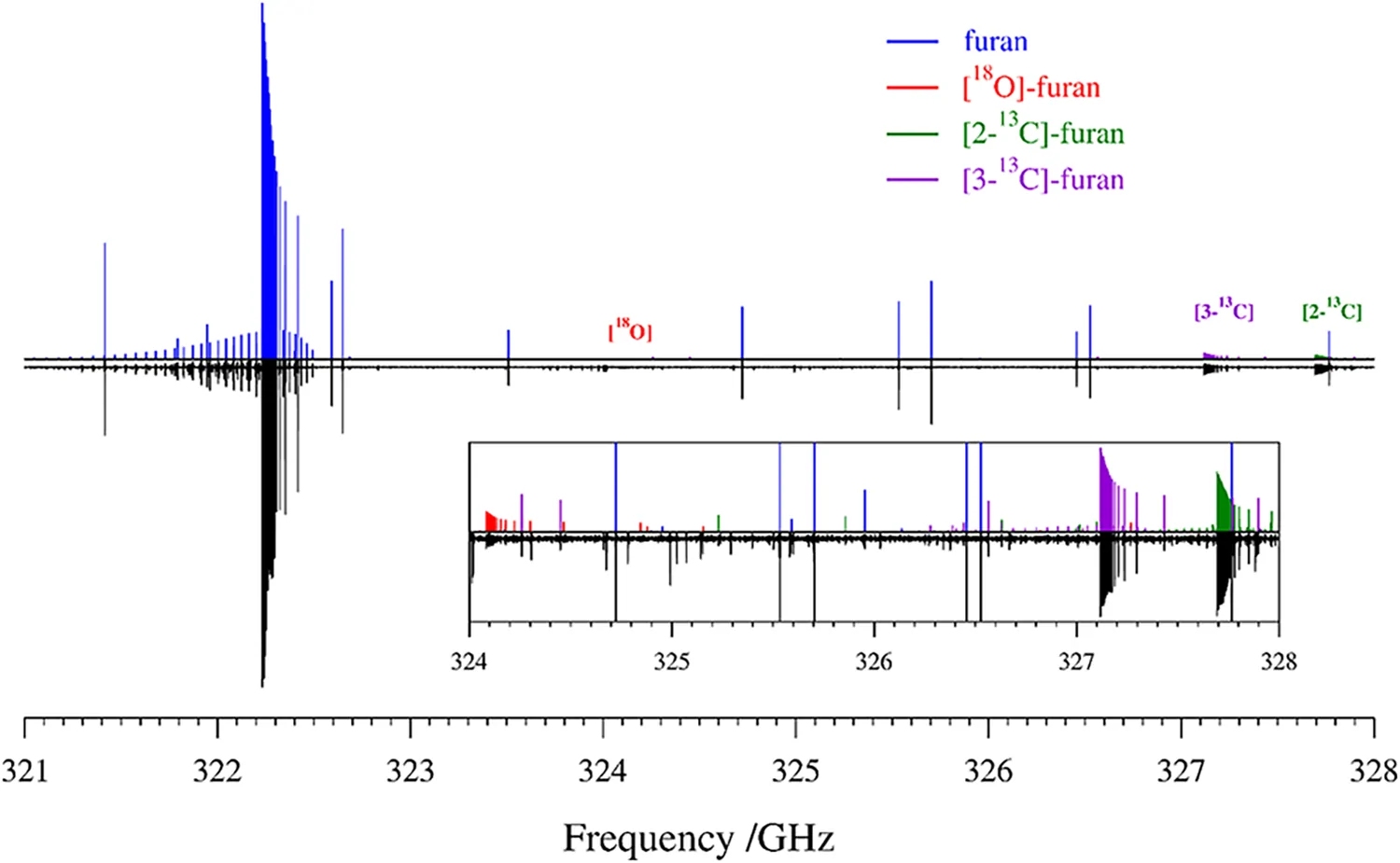
Experimental rotational spectrum (bottom) and predicted stick spectra (top) of furan and its heavy-atom isotopologues in the range of 321 to 328 GHz. The inset spectrum shows the region from 324 to 328 GHz to show the R-branch bands of the heavy-atom isotopologues. The unlabeled spectral features have been assigned to vibrationally excited states of furan.

Initial predictions for the ground vibrational state of the normal isotopologue of furan were based on prior works.^9–11^ Over 4300 rotational transitions were measured, assigned, and least-squares fit to a complete sextic distorted-rotor Hamiltonian in both the A reduction, I representation and S reduction, III representation. As shown by the data distribution plot in Figure 3, the data set incorporates a wide range of quantum numbers, covering a *J*″ range of 0 to 92 and a *K*_*a*_″ range of 0 to 60. The missing R-branch *J*″ and *K*_*a*_″ transitions above *J*″ = 45 are due to the R-branch “collapsing” onto itself as the spectrum increases in frequency, causing individual transitions to be unresolvable. At higher frequencies, some transitions once again became resolvable and were measured and assigned. Unsurprisingly, the A-reduced least-squares fit in the III representation exhibited a breakdown in the Hamiltonian, which struggled to model the high *K*_*a*_/low *K*_*c*_ transitions. This behavior is well-known and has been observed with other near-oblate asymmetric tops.^35,37,41,61–66^ Due to this breakdown, only the A reduction, I representation and S reduction, III representation were utilized for the analyses presented in this work. Experimental and computed spectroscopic constants for the normal isotopologue in the A reduction, I^*r*^ representation and S reduction, III^*r*^ representation are provided in Table 1. For the normal isotopologue of furan, there is generally good agreement between the experimental and computed rotational and centrifugal distortion constants, except for the CCSD­(T)/cc-pCVTZ computed value of Φ_*JK*_, which is 45% greater than the experimentally determined value. While the origin of the larger deviation of Φ_*JK*_ is unclear, this deviation does not stand out as unusually concerning.

**Figure 3:**
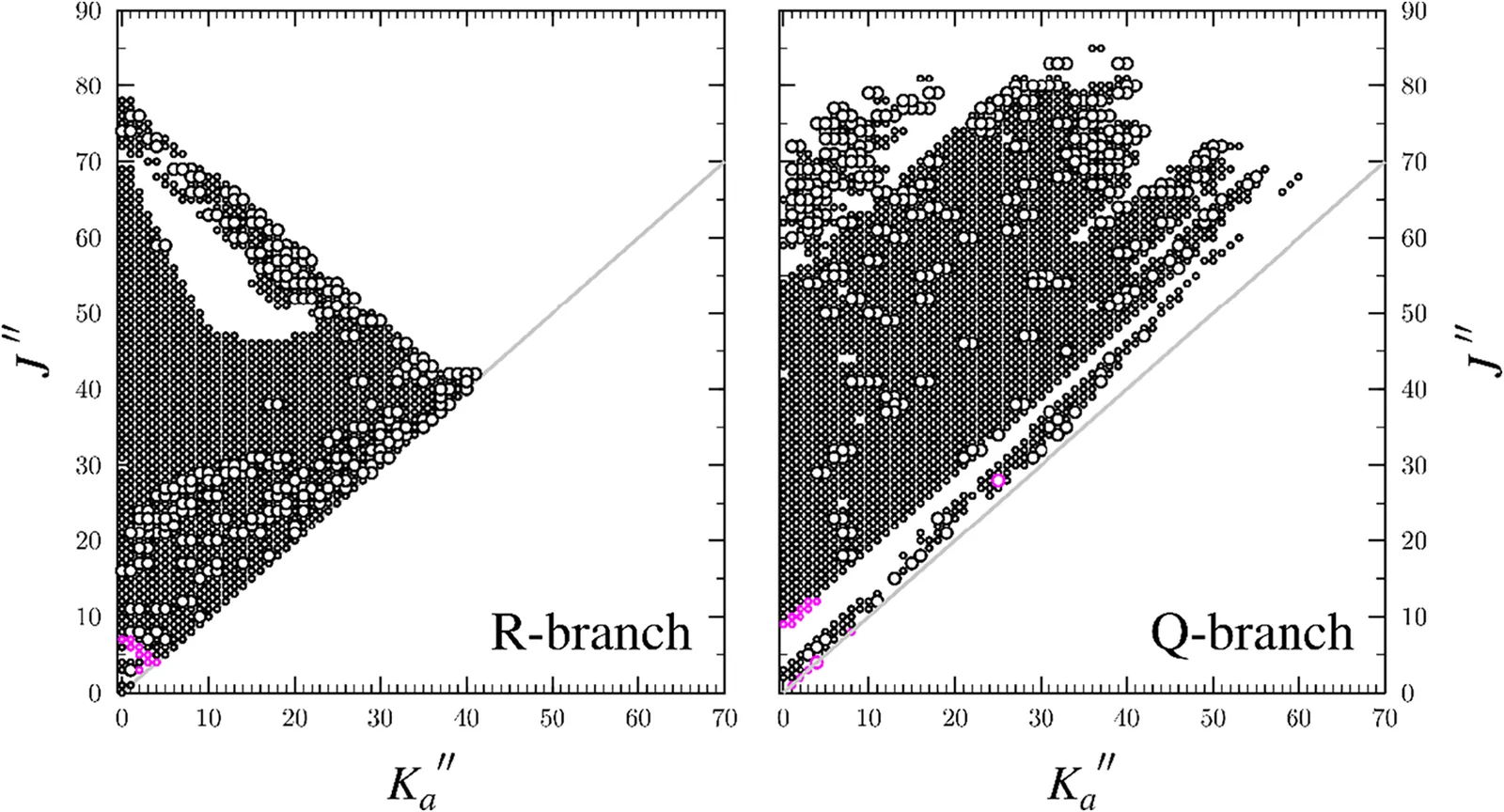
Data distribution plot for the least-squares fit of spectroscopic data for the vibrational ground state, normal isotopologue of furan. Black circles are measurements from the current work, while pink circles are from prior works.^3,10,25^ The size of the outlined circle is proportional to the value of |(*fobs*. – *fcalc.*)/*δf*|, where δ*f* is the frequency measurement uncertainty, and all values represented are less than or equal to 3.

**Table 1:** Spectroscopic Constants for the Ground Vibrational State of Furan, Normal Isotopologue (A Reduction, I^*r*^ Representation and S Reduction, III^*r*^ Representation)

A Reduction, I^*r*^ Representation				S Reduction, III^*r*^ Representation^a^			
	experimental^b^	CCSD(T)^c^	B3LYP^d^,^e^		experimental^b^	CCSD(T)^c^	B3LYP^d^
*A*_0_ (MHz)	9447.120 653 (29)	9381	9403	*A*_0_ (MHz)	9447.122 669 (29)	9381	9403
*B*_0_ (MHz)	9246.745 723 (28)	9171	9250	*B*_0_ (MHz)	9246.743 252 (28)	9171	9250
*C*_0_ (MHz)	4670.824 577 (30)	4635	4661	*C*_0_ (MHz)	4670.824 931 (30)	4635	4661
Δ_*J*_ (kHz)	1.749 299 (13)	1.71	1.72	*D*_*J*_ (kHz)	3.334 341 (16)	3.27	3.29
Δ_*JK*_ (kHz)	–0.249 163 (27)	–0.207	–0.226	*D*_*JK*_ (kHz)	–5.263 773 (23)	–5.15	–5.19
Δ_*K*_ (kHz)	1.850 066 (34)	1.78	1.82	*D*_*K*_ (kHz)	2.282 523 (18)	2.23	2.25
δ_*J*_ (kHz)	0.698 106 0 (29)	0.682	0.687	*d*_1_ (kHz)	–0.051 173 4 (33)	–0.052 9	–0.055 5
δ_*K*_ (kHz)	1.319 101 (14)	1.30	1.29	*d*_2_ (kHz)	0.043 246 8 (30)	0.044 4	0.042 5
Φ_*J*_ (Hz)	0.000 740 8 (18)	0.000 719		*H*_*J*_ (Hz)	0.001 464 8 (23)	0.001 45	0.001 44
Φ_*JK*_ (Hz)	0.000 292 1 (88)	0.000 423		*H*_*JK*_ (Hz)	–0.006 098 7 (56)	–0.005 95	–0.005 96
Φ_*KJ*_ (Hz)	–0.005 339 (22)	–0.005 40		*H*_*KJ*_ (Hz)	0.007 792 5 (75)	0.007 55	0.007 60
Φ_*K*_ (Hz)	0.005 386 (22)	0.005 31		*H*_*K*_ (Hz)	–0.003 162 2 (37)	–0.003 05	–0.003 08
φ_*J*_ (Hz)	0.000 372 12 (57)	0.000 360		*h*_1_ (Hz)	–0.000 087 0 (20)	–0.000 083 2	–0.000 103
φ_*JK*_ (Hz)	0.000 794 1 (49)	0.000 848		*h*_2_ (Hz)	–0.000 094 3 (21)	–0.000 101	–0.000 090 1
φ_*K*_ (Hz)	0.002 910 4 (75)	0.002 77		*h*_3_ (Hz)	–0.000 016 19 (57)	–0.000 012 9	–0.000 016 0
*N*_lines_ (rot)^f^	4344			*N*_lines_ (rot)^f^	4344		
σ_fit_ (MHz)	0.027			σ_fit_ (MHz)	0.027		
κ^g^	0.916 096	0.912	0.931	κ^g^	0.916 094	0.912	0.931
Δ_*i*_ (μÅ^2^)^h^	0.048 742 (1)	0.056 6	0.056 9	Δ_*i*_ (μÅ^2^)^h^	0.048 730 (1)	0.056 6	0.056 9

a Left-handed output from SPFIT was converted to right-handed for presentation.

b Includes transitions from previous works.^3,10,25^

c Evaluated using the cc-pCVTZ basis set.

d Evaluated using the 6–311+G­(2d,p) basis set.

e Centrifugal distortion constants calculated directly from the *B*_*e*_ constants and the τ centrifugal distortion constants using the equations provided by Gordy and Cook in Tables 8.14 and 8.16.^60^

f Number of independent transition frequencies.

g κ = (2*B* – *A* – *C*)/(*A* – *C*) calculated from *B*_0_ values using PLANM.

h Inertial defect (Δ_*i*_ = *I_c_ – I_a_ – I_b_)* calculated from *B*_0_ values using PLANM.

Following the analysis of the normal isotopologue, the ^18^O- and ^13^C-substituted furan isotopologues, furan-*d*_4_, and the synthesized furan-*d*_*x*_ sample were analyzed in a similar manner. For brevity, the spectroscopic constants and data distribution plots of the isotopically substituted furan species are provided in the Supporting Information. As shown in Figure 4, all possible deuterium-substituted isotopologues were visible within the spectrum of the furan-*d*_*x*_ sample, allowing for the assignment and measurement of the deuterium-substituted isotopologues, as well as all singly ^13^C-substituted isotopologues of each of the deuterium-substituted species at natural abundance. In the synthesized sample, the presence of multiple isotopic species means that each individual ^13^C-substituted isotopologue is present in very low abundance, and therefore at low partial pressure, making the absolute intensity of these species’ rotational transitions quite low. The ^18^O-substituted isotopologues in the furan-*d*_*x*_ sample were unobservable in the millimeter-wave spectrum due to their low natural abundance. Although ^18^O-substituted isotopologues were observed in the microwave spectrum, too few transitions could be measured (<4) to obtain reliable rotational constants via least-squares fitting.

**Figure 4:**
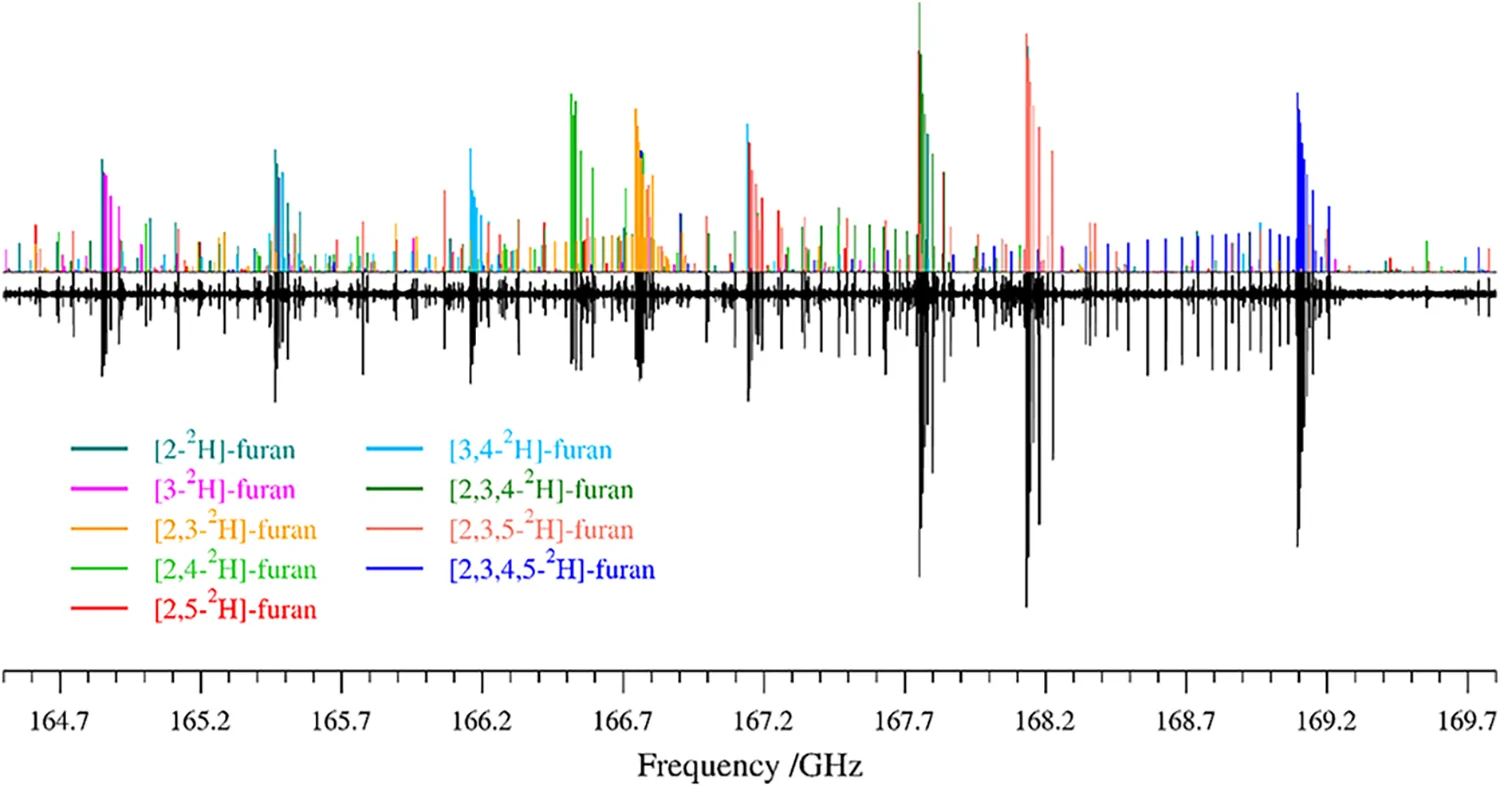
Experimental rotational spectrum (bottom) of furan-*d*_*x*_ and predicted stick spectra (top) of deuterium-substituted furan isotopologues from experimental spectroscopic constants in the range of 164.5 to 169.8 GHz.

Since most isotopologues are present in very low abundance within the furan-*d*_*x*_ sample, assignment of their transitions is challenging due to the presence of transitions from many isotopologues and their vibrationally excited states. While rotational constants computed at the CCSD­(T)/cc-pCVTZ level are typically helpful for assigning species with intense transitions—a situation for which the accuracy of the prediction is less important—the accuracy of the computed constants was not sufficient for assigning transitions of low-abundance furan species. Instead, we corrected the equilibrium rotational constants (*B*_*e*_^xrefit^) from a preliminary *r*_*e*_^SE^ structure from the already analyzed isotopologues with the computed vibration–rotation interaction and electron-mass corrections to achieve a better approximation of the *B*_0_ values. The average residual, or difference from experimental value, of *A*_0_, *B*_0_, and *C*_0_ derived using the *B*_*e*_^xrefit^ constants from the *r*_*e*_^SE^(final) structure determination (*vide infra*) is −0.141 (59) MHz, −0.068 (51) MHz, and 0.026 (8) MHz, respectively (Table S7). In contrast, the CCSD­(T)/cc-pCVTZ *A*_0_, *B*_0_, and *C*_0_ have average residuals of −40.5 (6.9) MHz, −33.6 (8.3) MHz, and −18.7 (1.1) MHz (Table S7). Therefore, the highly accurate *B*_0_ constants from preliminary *B*_*e*_^xrefit^ values were used to predict the rotational spectra of low-intensity species and/or species present in high-density areas of the spectrum; when used in combination with predicted centrifugal distortion constants from the VPT2 calculation, predicted transition frequencies typically fell within 50 kHz of the observed line center in the microwave spectra (<18.5 GHz) and within a few MHz of the transition at higher frequencies in the millimeter-wave spectra. As more isotopologues were measured, least-squares fit, and incorporated into the *r*_*e*_^SE^ structure, the accuracy of the *B*_*e*_^xrefit^ values for as yet unassigned isotopologues improved. In total, transitions of 44 furan isotopologues were measured and fit, and the rotational constants for all the isotopologues were used in the subsequent *r*_*e*_^SE^ structures. In the case that a centrifugal distortion constant could not be confidently determined from the least-squares fitting, the constant was instead held at the CCSD­(T)/cc-pCVTZ calculated value. The least-squares fitting output files for each isotopologue are available in the Supporting Information.

The normal isotopologue of furan did not display ^1^H-hyperfine splitting within the measured spectral region. The furan-*d*_*x*_ sample revealed partially resolved ^2^H-hyperfine splitting in the microwave region (<18.5 GHz) of the spectrum. This splitting, however, was not analyzed as part of this work and the hyperfine-free line center was used in the least-squares fitting where applicable. Due to the small residual effect of the unresolved hyperfine splitting on the measured line center, the measurement uncertainty of these transitions was estimated to be 25 kHz.

### Initial Semi-Experimental Structure Determination

Similar to prior *r*_*e*_^SE^ structure determinations,^30–41^ the ground-state rotational constants of each isotopologue in the A and S reductions (*B*_0_^(A)^ and *B*_0_^(S)^) were converted to determinable rotational constants (*B*_0_″^(A)^ and *B*_0_″^(S)^), which removes the effects of centrifugal distortion.^60^ The determinable constants and the equations used to calculate them are provided in Supporting Information. The *B*_0_″^(A)^ and *B*_0_″^(S)^ values differed by less than 0.01 MHz for every isotopologue (Table S5), providing confidence that each fit adequately treated the data set and that the resultant average value of the determinable constants is sufficiently well determined to be used in the *r*_*e*_^SE^ structure determination. The experimentally determined and averaged *B*_0_″ values of the 44 furan isotopologues provide 88 independent moments of inertia, allowing for a significant overdetermination of the eight structural parameters of furan. As mentioned previously, the computed vibration–rotation interaction and electron-mass corrections were input into the *xrefit* module of CFOUR along with the averaged *B*_0_″ values to determine the equilibrium structural parameters of furan by least-squares fitting.

To assess the quality of the determinable rotational constants and the computed corrections used in the *r*_*e*_^SE^ structure determination, the inertial defects (Δ_*i*_) of each isotopologue were compared. By definition, the equilibrium inertial defect (Δ_*ie*_) of a planar molecule must be exactly zero. Table 2 shows the inertial defects of each furan isotopologue determined from five treatments of the experimental rotational constants. The observable ground-state inertial defects (Δ_*i*0_) for furan are small positive values, similar to those observed for other aromatic heterocycles.^30–32,34,35,38,39,41^ When vibration–rotation interaction corrections are applied to the determinable constants, the resulting Δ_*ie*_ values are overcorrected and become negative, but are closer to the true value of zero by an order of magnitude. Further correcting the rotational constants for the electron-mass distribution results in positive Δ_*ie*_ values that are even closer to zero, indicating that the corrections generally treated the determinable constants in a consistent manner. This pattern of reducing inertial defect magnitudes with further corrections is in alignment with prior works.^30–41^ The fully corrected Δ_*ie*_ values of each isotopologue are in close agreement with the average fully corrected Δ_*ie*_ (as evidenced by the small *s* value), providing confidence that each is well-modeled and appropriate for use in determining the *r*_*e*_^SE^ structure.

**Table 2:** Inertial Defects (Δ_*i*_) of Furan Isotopologues with and without Vibration-Rotation Interaction and Electron-Mass Distribution Corrections

		*r*_*e*_^SE^(initial)		*r*_*e*_^SE^(final)	
isotopologue	Δ_*i*0_ (μÅ^2^)	Δ_*ie*_ (μÅ^2^)^a^	Δ_*ie*_ (μÅ^2^)^b^	Δ_*ie*_ (μÅ^2^)^c^	Δ_*ie*_ (μÅ^2^)^d^
furan	0.04866	–0.00695	0.00122	–0.00607	0.00211
[^18^O]	0.04972	–0.00694	0.00124	–0.00624	0.00194
[2-^13^C]	0.04917	–0.00711	0.00107	–0.00610	0.00208
[3-^13^C]	0.04929	–0.00701	0.00117	–0.00608	0.00210
[2-^2^H]	0.04715	–0.00715	0.00103	–0.00607	0.00211
[2-^2^H, 2-^13^C]	0.04751	–0.00714	0.00104	–0.00606	0.00212
[2-^2^H, 3-^13^C]	0.04744	–0.00746	0.00072	–0.00639	0.00179
[2-^2^H, 4-^13^C]	0.04764	–0.00725	0.00093	–0.00615	0.00203
[2-^2^H, 5-^13^C]	0.04753	–0.00719	0.00099	–0.00614	0.00204
[3-^2^H]	0.04871	–0.00706	0.00112	–0.00606	0.00212
[3-^2^H, 2-^13^C]	0.04917	–0.00710	0.00108	–0.00608	0.00210
[3-^2^H, 3-^13^C]	0.04906	–0.00715	0.00103	–0.00615	0.00203
[3-^2^H, 4-^13^C]	0.04897	–0.00738	0.00080	–0.00641	0.00177
[3-^2^H, 5-^13^C]	0.04905	–0.00723	0.00095	–0.00619	0.00199
[2,3-^2^H]	0.04734	–0.00715	0.00104	–0.00603	0.00215
[2,3-^2^H, 2-^13^C]	0.04774	–0.00700	0.00118	–0.00596	0.00222
[2,3-^2^H, 3-^13^C]	0.04772	–0.00723	0.00095	–0.00610	0.00208
[2,3-^2^H, 4-^13^C]	0.04789	–0.00709	0.00109	–0.00603	0.00215
[2,3-^2^H, 5-^13^C]	0.04780	–0.00709	0.00109	–0.00599	0.00219
[2,4-^2^H]	0.04698	–0.00718	0.00100	–0.00603	0.00215
[2,4-^2^H, 2-^13^C]	0.04737	–0.00713	0.00105	–0.00598	0.00221
[2,4-^2^H, 3-^13^C]	0.04754	–0.00725	0.00093	–0.00610	0.00208
[2,4-^2^H, 4-^13^C]	0.04746	–0.00710	0.00108	–0.00596	0.00223
[2,4-^2^H, 5-^13^C]	0.04741	–0.00723	0.00095	–0.00607	0.00211
[2,5-^2^H]	0.04555	–0.00709	0.00109	–0.00601	0.00217
[2,5-^2^H, 2-^13^C]	0.04600	–0.00699	0.00119	–0.00590	0.00228
[2,5-^2^H, 3-^13^C]	0.04612	–0.00711	0.00107	–0.00603	0.00216
[3,4-^2^H]	0.04892	–0.00694	0.00124	–0.00601	0.00217
[3,4-^2^H, 2-^13^C]	0.04937	–0.00694	0.00124	–0.00599	0.00219
[3,4-^2^H, 3-^13^C]	0.04947	–0.00688	0.00130	–0.00593	0.00225
[2,3,4-^2^H]	0.04730	–0.00713	0.00105	–0.00598	0.00220
[2,3,4-^2^H, 2-^13^C]	0.04763	–0.00714	0.00104	–0.00597	0.00221
[2,3,4-^2^H, 3-^13^C]	0.04779	–0.00707	0.00111	–0.00595	0.00223
[2,3,4-^2^H, 4-^13^C]	0.04769	–0.00716	0.00102	–0.00601	0.00217
[2,3,4-^2^H, 5-^13^C]	0.04769	–0.00719	0.00099	–0.00602	0.00216
[2,3,5-^2^H]	0.04553	–0.00717	0.00101	–0.00599	0.00219
[2,3,5-^2^H, 2-^13^C]	0.04590	–0.00712	0.00106	–0.00595	0.00223
[2,3,5-^2^H, 3-^13^C]	0.04596	–0.00720	0.00098	–0.00600	0.00219
[2,3,5-^2^H, 4-^13^C]	0.04614	–0.00714	0.00104	–0.00596	0.00222
[2,3,5-^2^H, 5-^13^C]	0.04592	–0.00710	0.00108	–0.00592	0.00226
[2,3,4,5-^2^H]	0.04560	–0.00704	0.00114	–0.00593	0.00226
[2,3,4,5-^2^H, ^18^O]	0.04652	–0.00701	0.00117	–0.00589	0.00229
[2,3,4,5-^2^H, 2-^13^C]	0.04593	–0.00703	0.00115	–0.00591	0.00227
[2,3,4,5-^2^H, 3-^13^C]	0.04605	–0.00706	0.00112	–0.00591	0.00227
					
Average (x̅)	0.04753	–0.00711	0.00107	–0.00604	0.00214
Std. Dev. (*s*)	0.00122	0.00011	0.00011	0.00011	0.00011

a Vibration–rotation interaction corrections only, computed at the CCSD­(T)/cc-pCVTZ geometry.

b Vibration–rotation interaction corrections and electron-mass distribution corrections computed at the CCSD­(T)/cc-pCVTZ geometry.

c Vibration–rotation interaction corrections only, computed at the *r*_*e*_^SE^(initial) geometry.

d Vibration–rotation interaction corrections computed at the *r*_*e*_^SE^(initial) geometry and electron-mass distribution corrections computed at the CCSD­(T)/cc-pCVTZ geometry.

Examination of the relative angle of rotation of the principal axes for each isotopologue compared to the normal isotopologue can reveal poorly corrected *A* and *B* rotational constants with large residuals that can be masked in the determination of the inertial defects, but may negatively impact the *r*_*e*_^SE^ structure. Following the methodology of previous works,^26,41^ the relative angle of rotation of the principal axes was calculated for each isotopologue to determine whether improved vibration–rotation interaction corrections were necessary for furan; the results are shown in Table 3. The analysis was performed using the initial semi-experimental equilibrium structure (*r*_*e*_^SE^(initial)); details of the analysis are provided in the Supporting Information. The rotational constant residuals (*B*_*e*_^xrefit^ – *B*_*e*_^SE^) from the *r*_*e*_^SE^(initial) structure determination are provided in Table 3. Focusing first on the case of [2-^13^C]-furan, which was previously identified as a problematic isotopologue in an *r*_*e*_^SE^ structure determination,^26^ we note that the improved level of theory for the geometry optimization and frequency calculation of this work afford a significantly improved θ*r*_*e*_ – θ*r*_*e*_^SE^ value of −2.47°, relative to the value of −18.6° obtained using DFT methods.^26^ Despite the improvement, this difference in rotation angle is still accompanied by large residuals of −1.17 and 1.06 MHz in *A*_*e*_ and *B*_*e*_, respectively (Table 3), though these values represent an improvement over the residuals of −2.21 and 2.03 MHz from the previous study. The difference in rotation of the *r*_*e*_ geometry relative to the *r*_*e*_^SE^ geometry causes an incorrect application of a portion of the *a*-axis and *b*-axis vibration–rotation interaction correction, manifesting as roughly equal and opposite *A*_*e*_ and *B*_*e*_ residuals with large magnitudes that stand out from the others. Analysis of the other isotopologues’ residuals and relative angles of rotation revealed three additional isotopologues with potential issues. Despite a small difference of only 0.21° between the *r*_*e*_ and *r*_*e*_^SE^ angles of rotation, the *A*_*e*_ and *B*_*e*_ values of [2,3-^2^H, 4-^13^C]-furan showed similarly large residuals of opposite sign (−0.84 and 0.71 MHz). Although the *A*_*e*_ and *B*_*e*_ values of [2,4-^2^H]- and [2,4-^2^H, 4-^13^C]-furan display small residuals, the differences in rotation angles are relatively large (−1.07° and −2.58°, respectively). Because of these concerning issues, the *A*_*e*_ and *B*_*e*_ constants of [2-^13^C]-, [2,3-^2^H, 4-^13^C]-, [2,4-^2^H]-, and [2,4-^2^H, 4-^13^C]-furan were excluded from the determination of the *r*_*e*_^SE^(initial) structure. Only the *C*_*e*_ rotational constants of these isotopologues were used in order to generate the best possible structure. Figure 5 illustrates the angle of rotation relative to the normal isotopologue in the *r*_*e*_^SE^(initial) structure for each isotopologue analyzed in this work and shows that there is a wide range of principal axis rotation angles within the data set. Consistent with the recent investigation of oxazole,^41^ no clear trends can be observed between the proximity of θ*r*_*e*_^SE^ to 45° and the resulting issues in the *r*_*e*_^SE^ structure determination, despite a prior suggestion that an angle near 45° may magnify the error.^26^ While [2-^13^C]-furan has a θ*r*_*e*_^SE^ value of 39.59°, other isotopologues have angles even closer to 45° (Table 3). For example, [2,4-^2^H, 3-^13^C]-furan has θ*r*_*e*_^SE^ of 43.10° and a difference in rotation angles of 0.71°, which is notably larger than the average difference in rotation angles. Yet the residuals for *A*_*e*_ and *B*_*e*_ (−0.13 and 0.02 MHz) for this isotopologue are typical and unremarkable.

**Figure 5:**
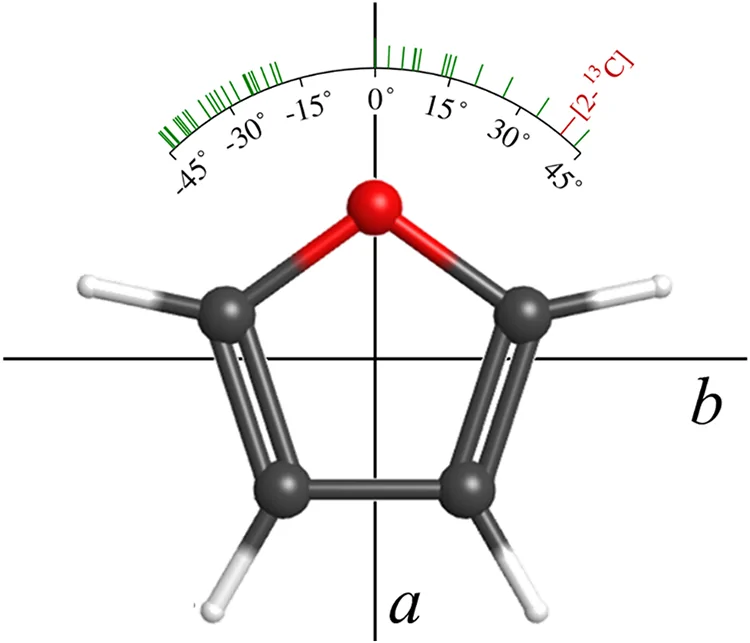
Normal isotopologue of furan with principal inertial axes. Each green dash represents the direction of the *a*-axis for an isotopologue studied in this work in the *r*_*e*_^SE^(initial) geometry. The value of angular rotation corresponds to θ*r*_*e*_^SE^ (°). [2-^13^C]-Furan is highlighted in maroon for convenience. The center of mass for each set of axes is shifted slightly (not displayed), but this shift does not affect the relative angles.

**Table 3:** Principal Axis Rotations *(*θ) Relative to the Normal Isotopologue of Furan and Rotational Constant Residuals (*B*_*e*_^xrefit^ – *B*_*e*_^SE^)^a^

isotopologue	θ*r*_*e*_ (°)^b^	θ*r*_*e*_^SE^ (°)^c^	θ*r*_*e*_ – θ*r*_*e*_^SE^ (°)	*A*_*e* residual_ (MHz)^c^	*B*_*e* residual_ (MHz)^c^	*C*_*e* residual_ (MHz)^c^
furan	0.00	0.00	0.00	–0.021	–0.045	0.037
[^18^O]	0.00	0.00	0.00	–0.039	–0.059	0.027
[2-^13^C]	37.12	39.59	–2.47	–1.168	1.065	0.027
[3-^13^C]	19.55	20.06	–0.51	–0.177	0.080	0.028
[2-^2^H]	–28.06	–27.64	–0.42	–0.141	0.088	0.032
[2-^2^H, 2-^13^C]	–18.95	–18.66	–0.28	–0.076	–0.005	0.022
[2-^2^H, 3-^13^C]	–24.41	–23.84	–0.57	–0.186	0.142	0.021
[2-^2^H, 4-^13^C]	–34.77	–34.40	–0.38	0.045	–0.081	0.025
[2-^2^H, 5-^13^C]	–25.30	–25.00	–0.30	–0.078	0.003	0.022
[3-^2^H]	–30.62	–30.92	0.29	–0.142	0.037	0.023
[3-^2^H, 2-^13^C]	–33.99	–34.37	0.38	–0.279	0.132	0.014
[3-^2^H, 3-^13^C]	–24.57	–24.89	0.32	–0.089	–0.021	0.015
[3-^2^H, 4-^13^C]	–31.68	–31.92	0.24	–0.091	0.012	0.013
[3-^2^H, 5-^13^C]	–37.04	–37.34	0.30	–0.359	0.208	0.014
[2,3-^2^H]	–42.38	–42.14	–0.24	–0.415	0.318	0.019
[2,3-^2^H, 2-^13^C]	–38.21	–37.97	–0.25	–0.140	0.011	0.011
[2,3-^2^H, 3-^13^C]	–43.64	–43.35	–0.29	–0.326	0.205	0.010
[2,3-^2^H, 4-^13^C]	–43.94	–43.73	–0.21	–0.837	0.707	0.009
[2,3-^2^H, 5-^13^C]	–39.37	–39.16	–0.21	–0.234	0.106	0.012
[2,4-^2^H]	34.73	33.66	1.07	–0.094	0.014	0.020
[2,4-^2^H, 2-^13^C]	26.56	26.04	0.52	–0.075	–0.031	0.013
[2,4-^2^H, 3-^13^C]	43.82	43.10	0.71	–0.126	0.020	0.011
[2,4-^2^H, 4-^13^C]	15.80	13.22	2.58	–0.068	–0.035	0.014
[2,4-^2^H, 5-^13^C]	15.52	15.01	0.51	–0.085	–0.017	0.011
[2,5-^2^H]	0.00	0.00	0.00	–0.018	–0.037	0.026
[2,5-^2^H, 2-^13^C]	2.62	2.59	0.03	–0.049	–0.054	0.017
[2,5-^2^H, 3-^13^C]	7.52	7.40	0.12	–0.033	–0.047	0.018
[3,4-^2^H]	0.00	0.00	0.00	–0.125	–0.026	0.009
[3,4-^2^H, 2-^13^C]	7.39	7.55	–0.16	–0.141	–0.034	0.002
[3,4-^2^H, 3-^13^C]	8.34	8.45	–0.11	–0.135	–0.047	0.003
[2,3,4-^2^H]	–39.56	–40.15	0.58	–0.223	0.092	0.007
[2,3,4-^2^H, 2-^13^C]	–40.40	–39.65	–0.75	–0.213	0.056	0.000
[2,3,4-^2^H, 3-^13^C]	–31.93	–32.73	0.80	–0.273	0.114	0.001
[2,3,4-^2^H, 4-^13^C]	–39.20	–39.61	0.42	–0.165	0.018	0.000
[2,3,4-^2^H, 5-^13^C]	–42.89	–42.40	–0.49	–0.306	0.152	–0.001
[2,3,5-^2^H]	–24.94	–24.69	–0.25	–0.092	0.005	0.015
[2,3,5-^2^H, 2-^13^C]	–19.80	–19.60	–0.20	–0.082	–0.036	0.007
[2,3,5-^2^H, 3-^13^C]	–21.66	–21.39	–0.27	–0.110	0.001	0.009
[2,3,5-^2^H, 4-^13^C]	–29.71	–29.45	–0.26	–0.115	–0.001	0.008
[2,3,5-^2^H, 5-^13^C]	–23.48	–23.28	–0.20	–0.111	–0.017	0.007
[2,3,4,5-^2^H]	0.00	0.00	0.00	–0.019	–0.106	0.003
[2,3,4,5-^2^H, ^18^O]	0.00	0.00	0.00	–0.025	–0.120	–0.002
[2,3,4,5-^2^H, 2-^13^C]	5.40	5.29	0.11	–0.037	–0.116	–0.004
[2,3,4,5-^2^H, 3-^13^C]	14.45	14.04	0.41	–0.086	–0.064	–0.003
						
Average (x̅)				–0.172	0.059	0.013
Std. Dev. (*s*)				0.211	0.207	0.010

a Calculation of θ values explained in the Supporting Information and by Demaison et al.^26^ and Esselman et al.^41^

b Calculated using the CCSD­(T)/cc-pCVTZ geometry.

c Calculated using the *r*_*e*_^SE^(initial) geometry.

### Final Semi-Experimental Structure Determination

Following our previously reported procedure to address the θ*r*_*e*_ – θ*r*_*e*_^SE^ discrepancies that lead to errors with the vibration–rotation interaction corrections,^41^ a new CCSD­(T)/cc-pCVTZ anharmonic frequency calculation was completed holding the atom positions of furan at the *r*_*e*_^SE^(initial) geometry. Even though the anharmonic frequency calculation was not performed at a stationary point on the computed potential energy surface and therefore inherently introduced an undetermined but small error into the cubic force constants, the improved vibration–rotation interaction constants rectified a larger error arising from an inaccurate evaluation of the *a*- and *b*-components of those constants. The new vibration–rotation interaction corrections were then applied to the averaged *B*_0_″ values, and a new *r*_*e*_^SE^(final) geometry was determined using all of the rotational constants of all 44 isotopologues. No rotational constants were excluded from the new structure determination because the new vibration–rotation constants mitigate the problems originating from differences in the angles of rotation in the initial data set. The effects of the new vibration–rotation interaction corrections can be assessed through the changes of the Δ_*ie*_ values (Table 2) and *A*_*e*_ and *B*_*e*_ residuals (Table S6). The overall effect of the improved constants on the fully corrected Δ_*ie*_ values is an increase in the mean by a factor of ∼2 with a similar standard deviation (Table 2). The improved residuals for many isotopologues provide more convincing evidence, in this case, for improvement to the vibration–rotation interaction corrections. The *A*_*e*_ and *B*_*e*_ residuals of [2-^13^C]-furan were reduced to −0.31 and 0.08 MHz, from −1.17 and 1.06 MHz, respectively. Those of [2,3-^2^H, 4-^13^C]-furan were reduced to −0.19 MHz and −0.03 MHz, from −0.84 and 0.71 MHz, respectively. While the residuals did not universally improve and some isotopologues showed a small increase in residuals using the final vibration–rotation interaction corrections, the standard deviations of the *A*_*e*_ and *B*_*e*_ residuals were reduced by a factor of ∼4 while the means remained at a similar value (Table S6). This behavior provides strong evidence that the final corrections more consistently treat each isotopologue by removing differential rotation of the *a*- and *b*-axes between the *r*_*e*_ and *r*_*e*_^SE^ geometries.

The *xrefiteration* plot showing the uncertainties of the *r*_*e*_^SE^(final) structure and the overall uncertainty of the *r*_*e*_^SE^(initial) structure is shown in Figure 6. (Figure S45 in the Supporting Information shows an analogous plot comparing all of the structural uncertainties of the *r*_*e*_^SE^(initial) and *r*_*e*_^SE^(final) structure determinations). For the *r*_*e*_^SE^(initial) structure, the total uncertainty declines until incorporation of the 36th isotopologue, and then gradually increases for the remaining isotopologues. This behavior is consistent with recent *r*_*e*_^SE^ structure determinations involving many isotopologues.^31,32,34,37–39^ The lowest overall uncertainty is obtained following the incorporation of the 35th isotopologue (δ*r*_*e*_^SE^(initial) = 0.000 154). An equivalently low structural uncertainty is never reached within the *r*_*e*_^SE^(final) structure determination, indicating that there is still a small systematic error in the *r*_*e*_^SE^(final) structure determination that raises the structural uncertainty. This error likely arises as a consequence of using a nonstationary point to calculate the cubic force constants used to determine the vibration–rotation interaction corrections. The structural uncertainty upon incorporating only the core set of isotopologues in the *r*_*e*_^SE^(final) determination is higher than that in the *r*_*e*_^SE^(initial) determination, which is likely due to the *A*_*e*_ and *B*_*e*_ rotational constants of [2-^13^C]-furan being included in the *r*_*e*_^SE^(final) determination that were excluded in the *r*_*e*_^SE^(initial) determination. The structural parameter uncertainties for the *r*_*e*_^SE^(final) structure determination, however, show a smooth decrease and an asymptotic behavior upon incorporation of the final isotopologues, further validating the *r*_*e*_^SE^(final) vibration–rotation interaction corrections.

**Figure 6:**
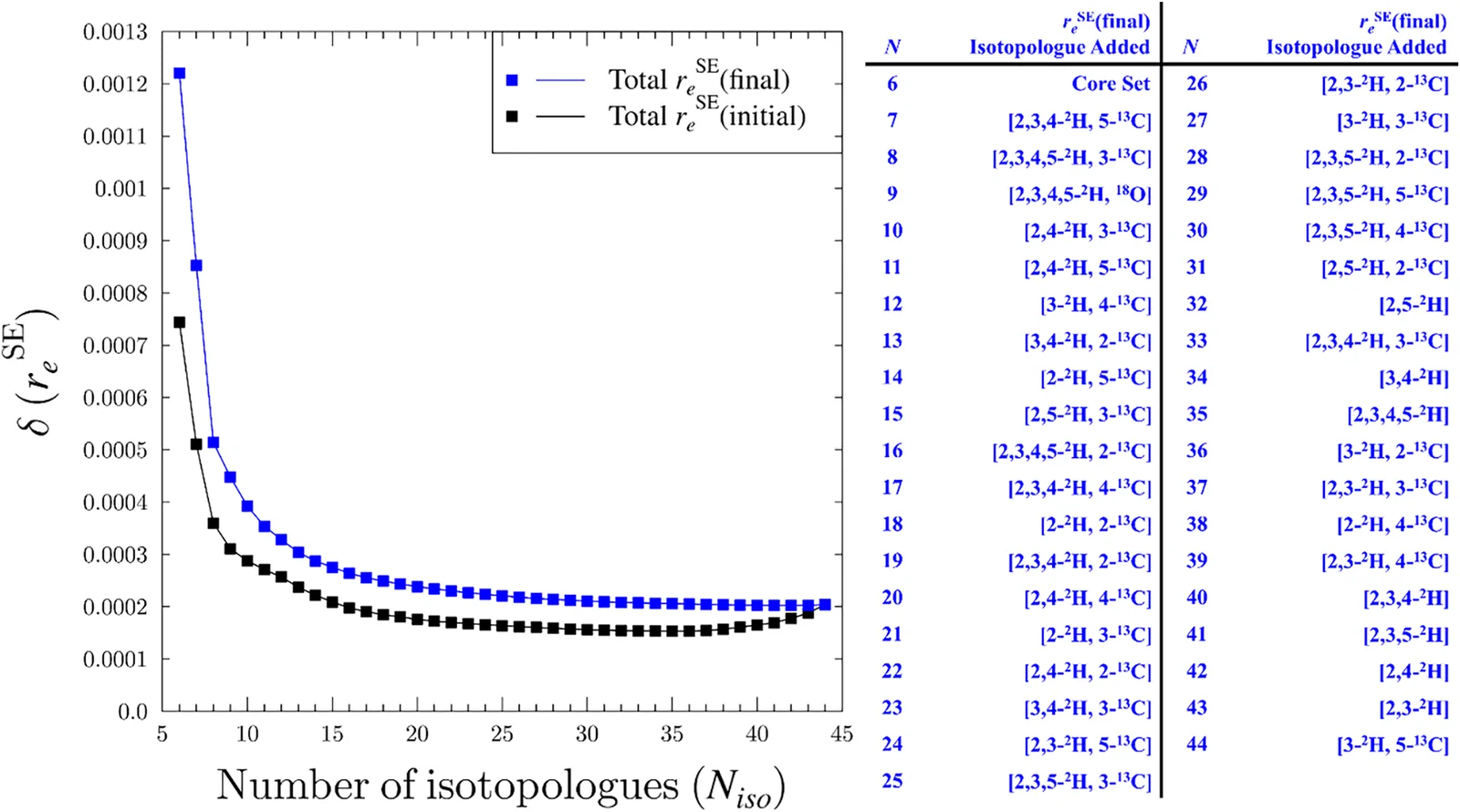
Plot of structural 2σ parameter uncertainty values (δ*r*_*e*_^SE^) as a function of the number of isotopologues (*N*_iso_) incorporated into the structure determination for furan. The total uncertainty of the *r*_*e*_^SE^(initial) structure determination utilized all three rotational constants for 40 isotopologues and only the *C* rotational constant for [2-^13^C]-, [2,3-^2^H, 4-^13^C]-, [2,4-^2^H]-, and [2,4-^2^H, 4-^13^C]-furan. The structural uncertainties of the *r*_*e*_^SE^(final) structure determination utilized all three rotational constants for all 44 isotopologues. Isotopologue ordering along the *x*-axis is provided in the table on the right for only the *r*_*e*_^SE^(final) structure determination.

Despite the overall structural uncertainty after the inclusion of all of the isotopologues being identical to three significant figures for the *r*_*e*_^SE^(final) structure determination (δ*r*_*e*_^SE^(initial) = 0.000 204, δ*r*_*e*_^SE^(final) = 0.000 204), comparison of the individual *xrefiteration* parameter plots (Figures 7a vs b, 8a vs b) suggests that the parameters of *r*_*e*_^SE^(final) geometry are likely to be more accurate than the *r*_*e*_^SE^(initial) geometry with vibration–rotation interaction corrections from the CCSD­(T)/cc-pCVTZ geometry. Figures 7 (bond lengths) and 8 (bond angles) show the impact of each isotopologue on the *r*_*e*_^SE^(initial) and *r*_*e*_^SE^(final) structural parameters. The *r*_*e*_^SE^(initial) parameter plots show abrupt changes to the parameters with the inclusion of the seventh, eighth, and/or 12th isotopologues (Figures 7a and 8a) and a gradual drift away from a seemingly converged value after the 40th isotopologue for several of the parameters involving C2. The *r*_*e*_^SE^(final) parameter plots (Figures 7b, and 8b), however, are remarkably asymptotic in comparison, showing well-converged structural parameters that do not deviate or show a substantial change in their statistical uncertainties after about the 20th isotopologue.

**Figure 7:**
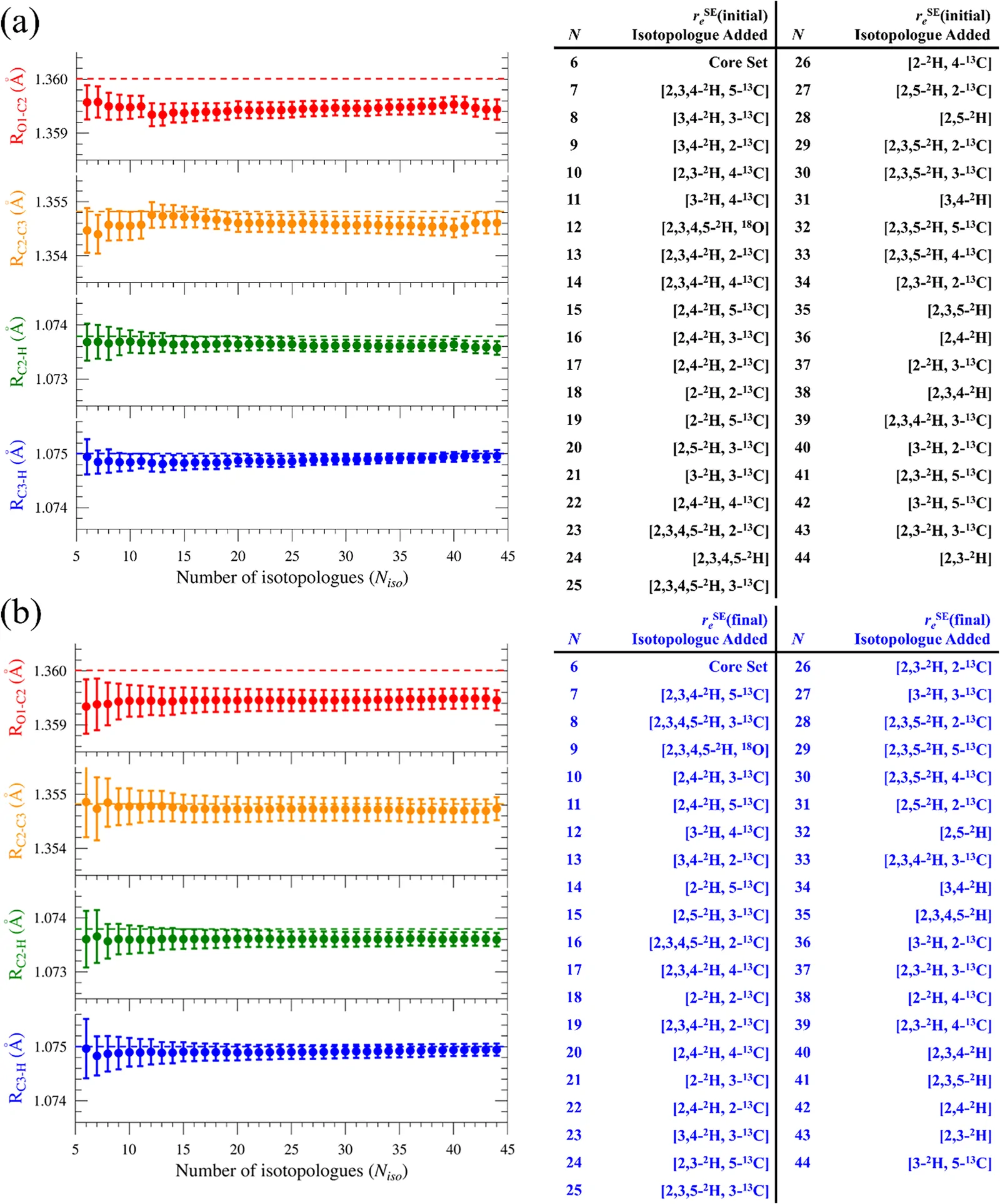
Plots of bond distances as a function of the number of isotopologues (*N*_iso_) and their 2σ uncertainties for (a) the *r*_*e*_^SE^(initial) geometry using all three rotational constants for 40 isotopologues and only the *C* rotational constant for [2-^13^C]-, [2,3-^2^H, 4-^13^C]-, [2,4-^2^H]-, and [2,4-^2^H, 4-^13^C]-furan, and (b) the *r*_*e*_^SE^(final) geometry using all three rotational constants for 44 isotopologues. Consistent scales are used for each bond distance (0.0020 Å). The colored, dashed line is the BTE value calculated for that parameter. Isotopologue ordering along the *x*-axis is provided in the tables on the right.

**Figure 8:**
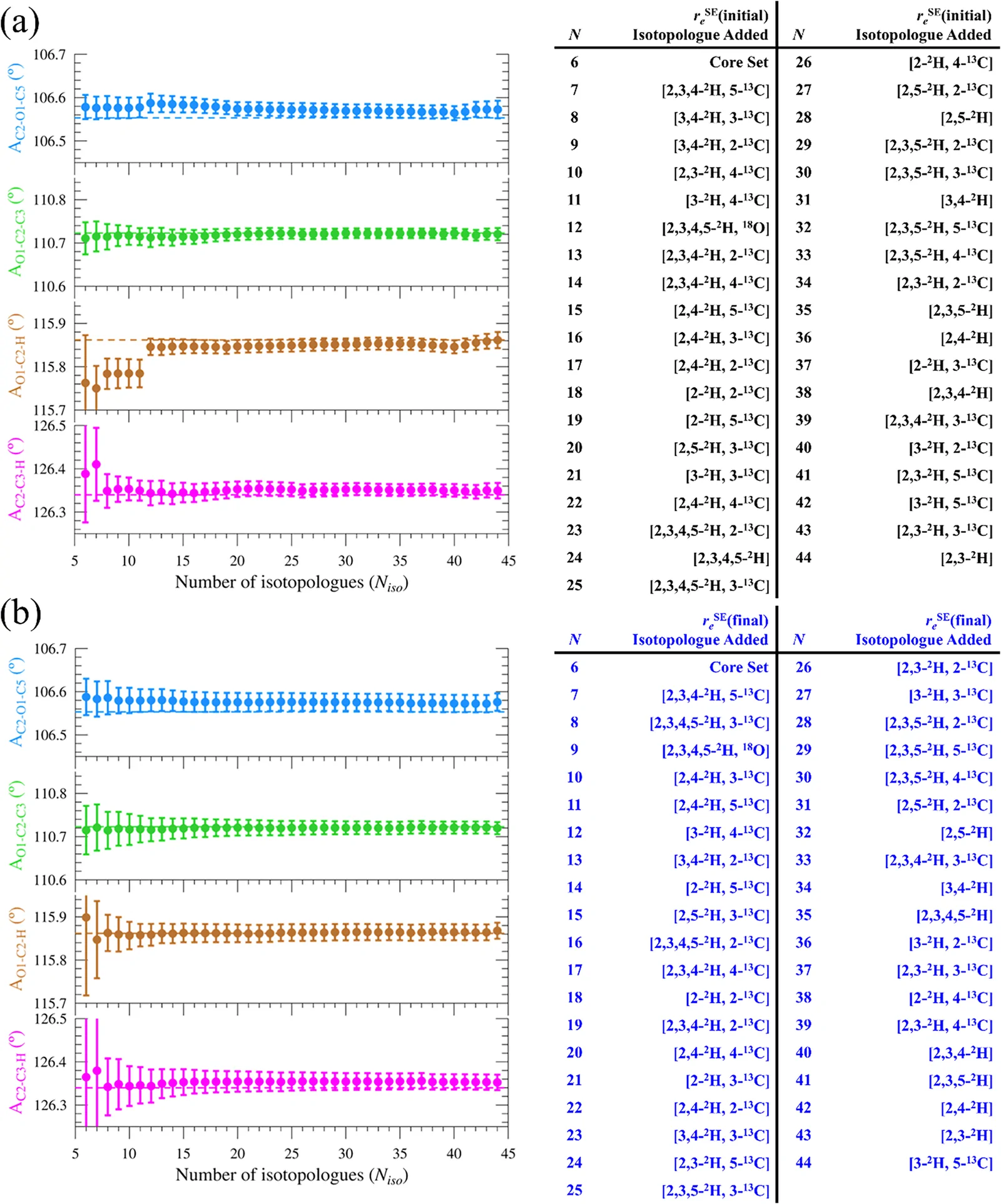
Plots of bond angles as a function of the number of isotopologues (*N*_iso_) and their 2σ uncertainties for (a) the *r*_*e*_^SE^(initial) geometry using all three rotational constants for 40 isotopologues and only the *C* rotational constant for [2-^13^C]-, [2,3-^2^H, 4-^13^C]-, [2,4-^2^H]-, and [2,4-^2^H, 4-^13^C]-furan, and (b) the *r*_*e*_^SE^(final) geometry using all three rotational constants for 44 isotopologues. Consistent scales are used for each bond angle (0.25°). The colored, dashed line is the BTE value calculated for that parameter. Isotopologue ordering along the *x*-axis is provided in the tables on the right.

The *r*_*e*_^SE^(final) and *r*_*e*_^SE^(initial) structures of furan are presented in Table 4 and Figure 9a,b. The accuracy and precision of the *r*_*e*_^SE^(final) structure is comparable to other molecular structures determined using the same methodology (most other determinations, however, use vibration–rotation interaction corrections calculated only at the computed *r*_*e*_ geometry),^30–41^ and the precision of each of the structural parameters is improved by an order of magnitude compared to the most recent *r*_*e*_^SE^ structure of furan (Figure 9d).^12^ Bond distances are determined with 2σ uncertainties ≤0.0003 Å, which are at or near the current limit of bond-length precision for the *r*_*e*_^SE^ structure determination methodology.^36,37^ Bond angles are also very well determined, with the largest uncertainty being 0.023° in the determination of the C3–C2–H angle. In general, there is a rather small effect due to the change in vibration–rotation interaction corrections, as many structural parameters showed little to no change between the initial and final structure determinations. Even for parameters that did change, the magnitude of the change fell within the uncertainty of the parameter. The parameter that had the largest change between the *r*_*e*_^SE^(initial) and *r*_*e*_^SE^(final) structures was the O–C2–H angle, which changed by only 0.007°, a value almost three times smaller than the corresponding 2σ uncertainty of 0.019°.

**Figure 9:**
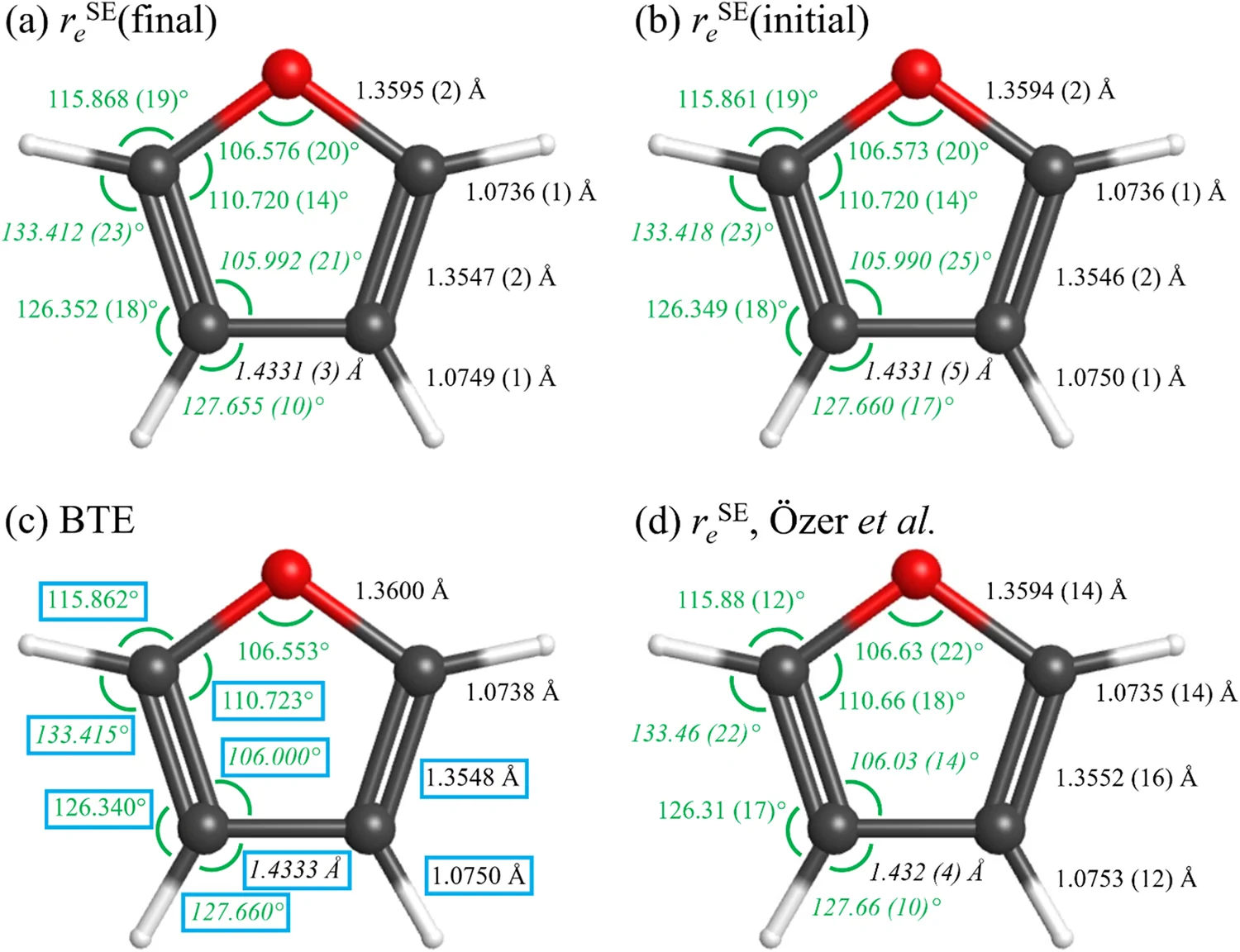
Semi-experimental equilibrium structures (*r*_*e*_^SE^) and theoretical BTE *r*_*e*_ structure of furan. Bond distances are shown in black and bond angles are shown in green, along with 2σ statistical uncertainties for experimental parameters. Parameters in italics were calculated using an alternate *Z*-matrix or geometric relationships. (a) *r*_*e*_^SE^(initial) structure from this work using all three rotational constants for 40 isotopologues and only the *C* rotational constant for [2-^13^C]-, [2,3-^2^H, 4-^13^C]-, [2,4-^2^H]-, and [2,4-^2^H, 4-^13^C]-furan. (b) *r*_*e*_^SE^(final) structure from this work using all three rotational constants for 44 isotopologues. (c) *r*_*e*_ BTE structure calculated at CCSD­(T)/cc-pCV5Z with additional corrections. Parameters within blue boxes agree with the corresponding *r*_*e*_^SE^(final) values. (d) *r*_*e*_^SE^ structure using 8 isotopologues from Özer et al.^12^

**Table 4:** Experimental and Computational Structural Parameters of Furan^a^ ^,^ ^b^

	*r*_*e*_^SE^ Özer et al.^12^	*r*_*e*_^SE^(initial)^c^	*r*_*e*_^SE^(final)^c^(Recommended)	CCSD(T) BTE^d^	CCSD(T)/cc-pCV5Z
*N*_iso_	8	44	**44**		
*N*_*I*_ ^e^	16	84	**88**		
*R*_O–C2_ (Å)	1.3594 (14)	1.3594 (2)	**1.3595 (2)**	**1.3600**	1.3586
*R*_C2–C3_ (Å)	1.3552 (16)	1.3546 (2)	**1.3547 (2)**	**1.3548**	1.3546
*R*_C2–H_ (Å)	1.0735 (14)	1.0736 (1)	**1.0736 (1)**	**1.0738**	1.0737
*R*_C3–H_ (Å)	1.0753 (12)	1.0750 (1)	**1.0749 (1)**	**1.0750**	1.0749
*R*_C3–C4_ (Å)	1.432 (4)	*1.4331 (5)*	*1.4331 (3)*	1.4333	1.4333
θ_O–C2–C3_ (°)	110.66 (18)	110.720 (14)	**110.720 (14)**	**110.723**	110.720
θ_O–C2–H_ (°)	115.88 (12)	115.861 (19)	**115.868 (19)**	**115.862**	115.912
θ_C2–C3–H_ (°)	126.31 (17)	126.349 (18)	**126.352 (18)**	**126.340**	126.364
θ_C2–O–C5_ (°)	106.63 (12)	106.573 (20)	**106.576 (20)**	**106.553**	106.613
θ_C3–C2–H_ (°)	133.46 (22)	*133.418 (23)*	*133.412 (23)*	133.415	133.367
θ_C4–C3–H_ (°)	127.66 (10)	*127.660 (17)*	*127.655 (10)*	127.660	127.663
θ_C2–C3–C4_ (°)	106.03 (14)	*105.990 (25)*	*105.992 (21)*	106.000	105.973

a Values in boldface facilitate comparison of best available experimental and computational data.

b Values in italics are a dependent parameter determined in an alternate *Z*-matrix or by mathematical relationships with propagated statistical uncertainty.

c Uncertainties reported are 2σ values.

d Evaluated using the cc-pCV5Z basis set with additional corrections.

e Number of independent moments of inertia.

### Structural Comparison to the BTE

In recent structure determinations of pyrimidine,^30^ 1*H*-1,2,4-triazole,^35^ and ketene,^37^ the *r*_*e*_^SE^ value of each structural parameter agreed with the corresponding BTE value within the uncertainty of the experimental value. For benzene,^36^ thiophene,^38^ and pyridine,^39^ however, one or more structural parameters fell outside of the experimental 2σ uncertainty in the parameter. As seen in Table 4, the *r*_*e*_^SE^(final) structure of furan falls into the latter category of agreement. While the majority of the BTE structural parameters (Figure 9c) agree with the *r*_*e*_^SE^(final) parameters (Figure 9a), the BTE values of the O–C2 and C2–H bond lengths and the C2–O–C5 bond angle fall just outside of the 2σ uncertainties of the *r*_*e*_^SE^(final) structure. The experimental parameters are very precisely determined, revealing a relatively small inaccuracy in the parameters of the BTE. Due to the very small actual differences in those parameters (*r*_*e*_^SE^(final) vs BTE, 0.0005 Å, 0.0002 Å, and 0.023°, respectively) the lack of agreement is unconcerning.

Figure 10 provides a direct comparison of the *r*_*e*_ and *r*_*e*_^SE^ structural parameters for furan. The *r*_*e*_ structures are derived from CCSD­(T)/cc-pCV*X*Z (*X* = 3, 4, or 5) and B3LYP/6–311+G­(2d,p) calculations. The *r*_*e*_^SE^(initial) and *r*_*e*_^SE^(final) structural parameters are presented with their corresponding 2σ uncertainties. As the size of the basis set increases, each CCSD­(T) *r*_*e*_ parameter displays the expected convergence behavior with the exception of the C2–C3–H bond angle (which necessitates the use of a two-point extrapolation to the complete basis set limit.) Some *r*_*e*_ parameters, such as the C2–H and O–C2 bond lengths, may be anticipated to show improved agreement between the *r*_*e*_ BTE and *r*_*e*_^SE^(final) structures by employing a larger basis set to determine the *r*_*e*_ geometry to which the BTE corrections are applied. In those cases, it appears that the expected decrease in the computed parameter and subsequent BTE correction may position the final value within the 2σ estimated statistical uncertainty of their corresponding *r*_*e*_^SE^(final) values, or at least move them closer. Owing to the large number of isotopologues, the tight convergence of the structural parameters, the general agreement between isotopologue inertial defect values, the small residuals of the isotopologue rotational constants, and the improved vibration–rotation interaction corrections utilized in the *r*_*e*_^SE^(final) structure, the discrepancies between the *r*_*e*_^SE^(final) structure and BTE structure are ascribed to theoretical limitations in evaluating the *r*_*e*_ geometry, the higher-order vibration–rotation interaction terms (γ), or the corrections used in determining the BTE.

**Figure 10:**
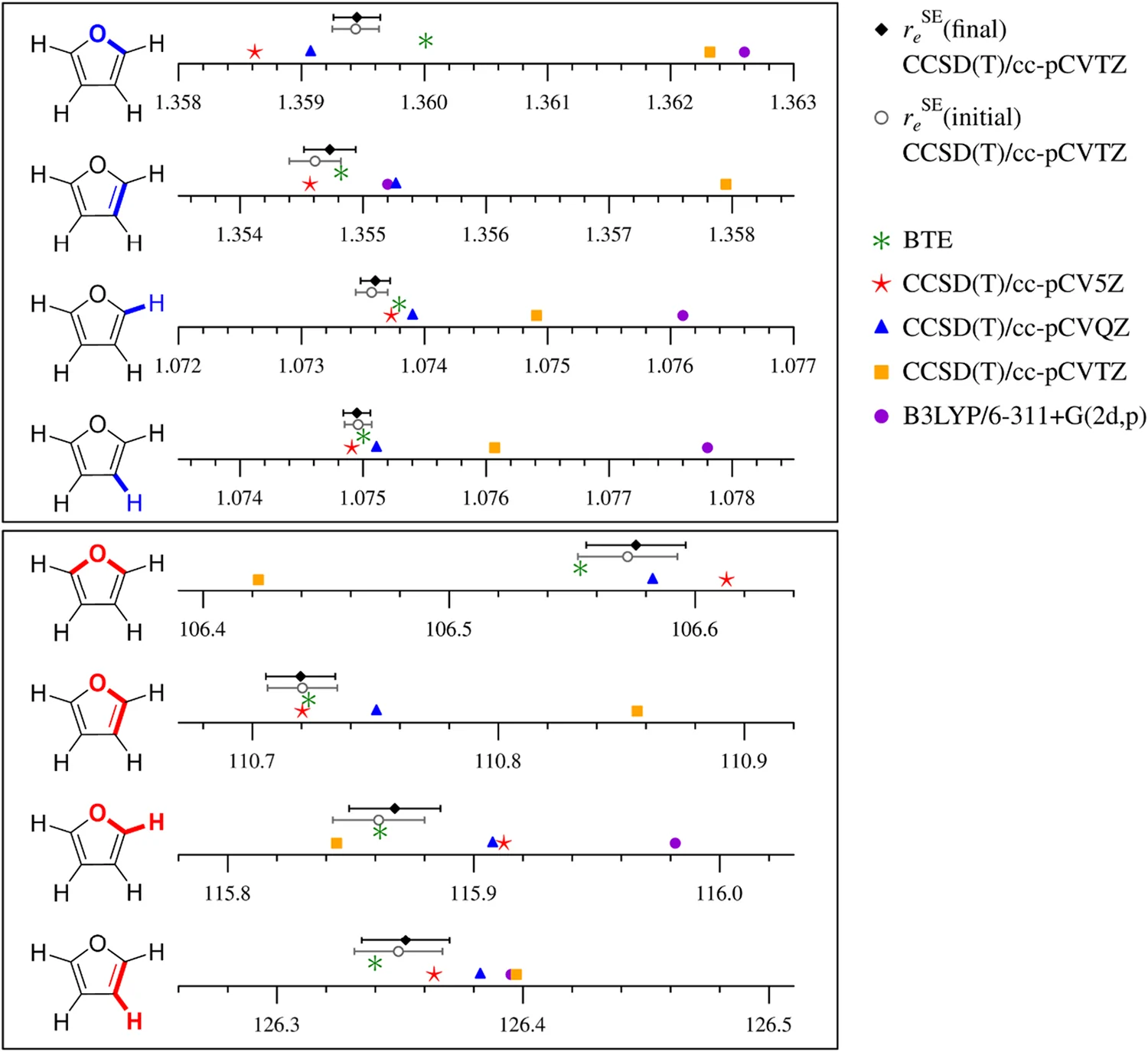
Graphical comparison of furan structural parameters with bond distances in Ångstrom (Å) and angles in deg (°). Uncertainties shown are 2σ. Bond distances and angles are shown with consistent ranges of 0.005 Å and 0.25°, respectively. The B3LYP/6–311+G­(2d,p) markers for θ_C2–O–C5_ and θ_O–C2–H_ are not visible because they are off scale.

### Structural Comparison to Thiophene

With an improved *r*_*e*_^SE^ structure of furan and the recently reported *r*_*e*_^SE^ structure of thiophene,^38^ the structural effects of substituting an oxygen atom with a sulfur atom within 5-membered aromatic heterocycles can be explored. Figure 11 shows the *r*_*e*_^SE^ structures of both furan and thiophene. Unsurprisingly, the O–C2 bond distance in furan is significantly shorter than the S–C2 bond distance in thiophene (1.36 Å vs 1.71 Å). There is also a pronounced difference in the C2–O–C5 vs C2–S–C5 bond angles (106.6° vs 92.1°) due to the differences in electronic properties^67,68^ of oxygen vs sulfur. The remaining structural differences between furan and thiophene are much more subtle. While both C–H bond lengths are slightly elongated in thiophene compared to furan, for both species the C2–H bond is slightly shorter than the C3–H bond. The structures of both furan and thiophene show bond length alternation between the C2–C3 and C3–C4 bond lengths. This effect is more pronounced in furan, which has a difference of 0.0784 Å between the C2–C3 and C3–C4 bond lengths, compared to a difference of 0.0564 Å within thiophene.

**Figure 11:**
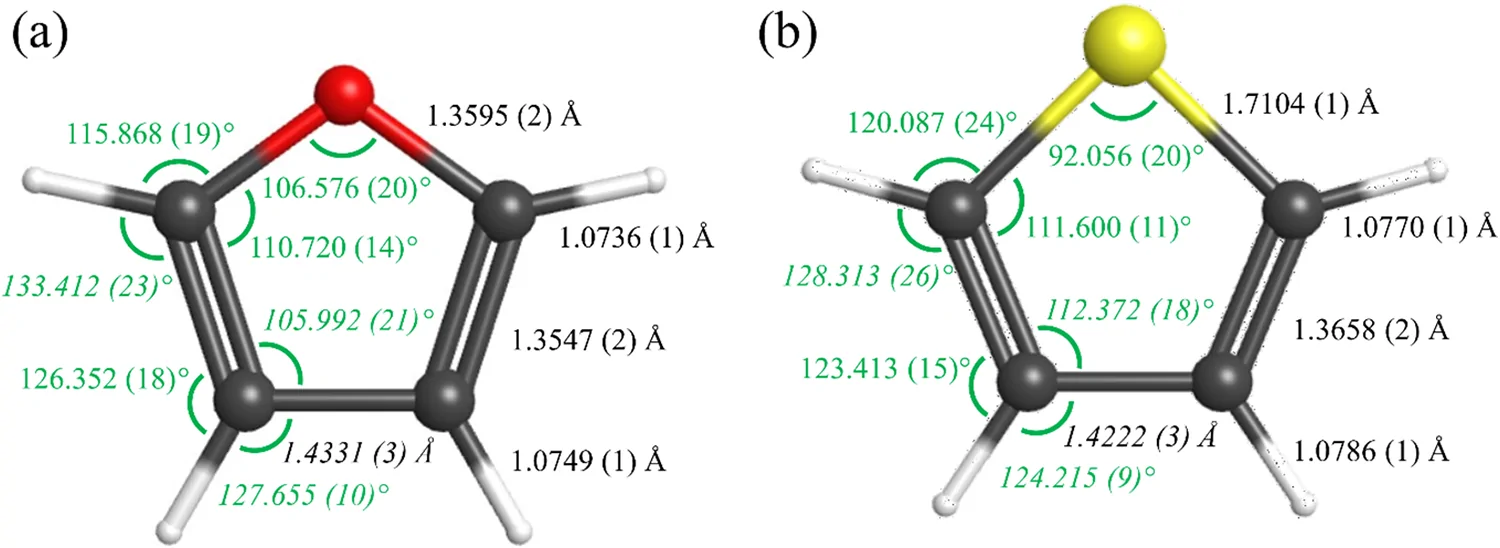
Structural parameters of (a) furan from the *r*_*e*_^SE^(final) structure determination (this work) and (b) thiophene from the *r*_*e*_^SE^ structure determination by Esselman et al.^38^ Bond distances in Ångstrom (Å) and angles in deg (°). Parameters in italics were calculated using an alternate *Z*-matrix.

Structural changes between furan and thiophene involving the carbon-centered bond angles are perhaps even more nuanced. Due to the geometric constraints of the 5-membered ring, the internal bond angles distort from the nominal *sp* ^2^ angle (120°) and instead are closer to the internal angle of a regular pentagon (108°) in both molecules. The X–C2–C3 (X = O or S) angles show similar values greater than 108° between both molecules (110.7° in furan, 111.6° in thiophene), presumably expanding to offset the contraction in the C2–X–C5 angle. The C2–C3–C4 angle within thiophene is also expanded, presumably further compensating for the small C2–S–C5 angle in thiophene. In furan, however, C2–C3–C4 is instead contracted, leading to an almost 6.5° difference and opposite behaviors in analogous angles between the molecules. The C3-centered angles (C2–C3–H and C4–C3–H) in furan (126.4° and 127.7°) and in thiophene (123.4° and 124.2°) are nearly equivalent in each individual structure, only differing by 1.3° in furan and 0.8° in thiophene, and are therefore about equivalently expanded from the ideal 120° geometry due to the contracted internal C2–C3–C4 angle in both molecules. The situation involving the C2-centered angles (X–C2–H and C3–C2–H) in furan and thiophene is significantly different. Within thiophene, the S–C2–H angle is 120.1° while the C3–C2–H angle is expanded to 128.3° to compensate for the contraction of the internal angle, resulting in a difference between these two angles of 8.2°. In the case of furan, this effect is even more pronounced. The O–C2–H angle is contracted to 115.9°, and the C3–C2–H angle is expanded to 133.4°, leading to a difference of 17.5° in these angles. We have also noticed a similar trend in the analogous C5-centered bond angles within thiazole^31^ and oxazole,^41^ which show comparable differences between the C4–C5–H and X–C5–H bond angles of 7.3° and 18.2°, respectively. The origin of the behavior of the C2-/C5-centered bond angles is not readily apparent and is likely the combination of multiple subtle factors.

## Conclusion

A new, highly accurate and precise semi-experimental equilibrium (*r*_*e*_^SE^) structure of furan (*c*-C_4_H_4_O) has been determined from rotational constants of 44 isotopologues, 35 of which were observed for the first time. The improved and newly determined spectroscopic constants can be used to support future astronomical searches for both the normal and isotopic furan species at frequencies up to 750 GHz. The *r*_*e*_^SE^(final) structure additionally validates the most recent *r*_*e*_^SE^ structure of furan by Özer et al.^12^ while improving the precision in the structural parameters by an order of magnitude.

The current study corrects for differences in the principal axis rotation between the computed (CCSD­(T)/cc-pCVTZ *r*_*e*_) structure and the *r*_*e*_^SE^ structure that occurs for some isotopologues of furan and other, near-oblate asymmetric top molecules.^41^ Application of methodology recently developed for the case of oxazole^41^ was used to correct for differences in principal axis rotation between the *r*_*e*_ and *r*_*e*_^SE^(initial) geometries to afford an improved *r*_*e*_^SE^(final) geometry for furan. Two conclusions can be drawn from the structure determinations of oxazole and furan regarding the importance of recalculating the vibration–rotation interaction constants at a geometry that closely matches the *r*_*e*_^SE^(final) structure. First, calculating the vibration–rotation interaction corrections at the best possible geometry is important. As we have shown, evaluating these corrections at what might otherwise be considered a very good computed geometry (e.g., CCSD­(T)/cc-pCVTZ) is not necessarily sufficient to obtain a structure of the highest accuracy and precision. Second, when a data set is determined to contain problematic isotopologues, a high quality structure can still be obtained by removing the problematic constants from the data set. As there is currently no way to know *a priori* which isotopologues may have poor θ*r*_*e*_ – θ*r*_*e*_^SE^ values or which ones will provide important structural information to constrain the structure, it remains the best practice to try to obtain as many isotopologues as feasible.

Due to the large number of isotopologues and the quality of the rotational constants utilized in the *r*_*e*_^SE^(final) structure determination, the lack of full agreement between theory and experiment is believed to originate from limitations of the computational/theoretical methods. Improvements in the calculation of the BTE *r*_*e*_ structure or improvements to the vibration–rotation interaction and electron-mass distribution corrections are needed to bring the structural parameters into better agreement. This verdict is consistent with the recent structure determinations of molecules with many isotopologues, particularly thiophene^38^ and oxazole,^41^ which also suggests that the discrepancies between the BTE structure and the *r*_*e*_^SE^ structures of molecules are theory-based. It remains unclear whether the structural parameters determined in this work are nearing or at the limit of *r*_*e*_^SE^ structure determinations. Further improvements to the evaluation of vibration–rotation interaction constants, such as the inclusion of higher-order terms (γ), are needed to refine our understanding of factors influencing high precision molecular structures and to assess the possible role of the breakdown of the Born–Oppenheimer approximation in these structures.

## Supplementary Material





## Notes

Equations used for calculating determinable constants and BTE corrections, isotopologue numbering scheme and dipole moments, tables of spectroscopic constants, data distribution plots for all isotopologues, additional information as mentioned in the text, experimental details for isotopologue synthesis and characterization (PDF)
Least-squares fitting files, computational output files, *xrefit* files, *xrefiteration* files, output files used to compute the BTE (ZIP)
