# Supplementary material for: Structure of Furan from 44 Isotopologues—Resolving Differences in the Theoretical (r e ) and Semi-Experimental Equilibrium (re SE) Structures
Source: J Phys Chem A. 2026 Sep 16;130(39):7783–800. doi: 10.1021/acs.jpca.6c04036 (PMC13637081; doi:10.1021/acs.jpca.6c04036)
Supplement: Supplementary file 1 [file jp6c04036_si_001.pdf]

Supporting Information

Structure of Furan from 44 Isotopologues – Resolving Differences in the Theoretical (r_e) and Semi-Experimental Equilibrium (r_e^{SE}) Structures

Kolton T. Mehalko,[1] Brian J. Esselman,[1] Peter R. Franke,[2]

Martin S. Holdren,[3] Gabi Wenzel,[3,4] D. Archie Stewart,[3] Zachary P. Burke,[3]

Brett A. McGuire,[3,5] R. Claude Woods,[1,*] Robert J. McMahon[1,*]

[1] *Department of Chemistry, University of Wisconsin−Madison, 1101 University Avenue, Madison, Wisconsin 53706, United States*

[2] *Quantum Theory Project, Departments of Physics and Chemistry, University of Florida, Gainesville, Florida 32611, United States*

[3] *Department of Chemistry, Massachusetts Institute of Technology, 77 Massachusetts Avenue, Cambridge, Massachusetts 02139, United States*

[4] *Center for Astrophysics | Harvard & Smithsonian, 60 Garden Street, Cambridge, Massachusetts 02138, United States*

[5] *National Radio Astronomy Observatory, 520 Edgemont Road, Charlottesville, Virginia 22903, United States*

* corresponding authors
E-mail address: rcwoods@wisc.edu (R.C. Woods).
E-mail address: robert.mcmahon@wisc.edu (R.J. McMahon).

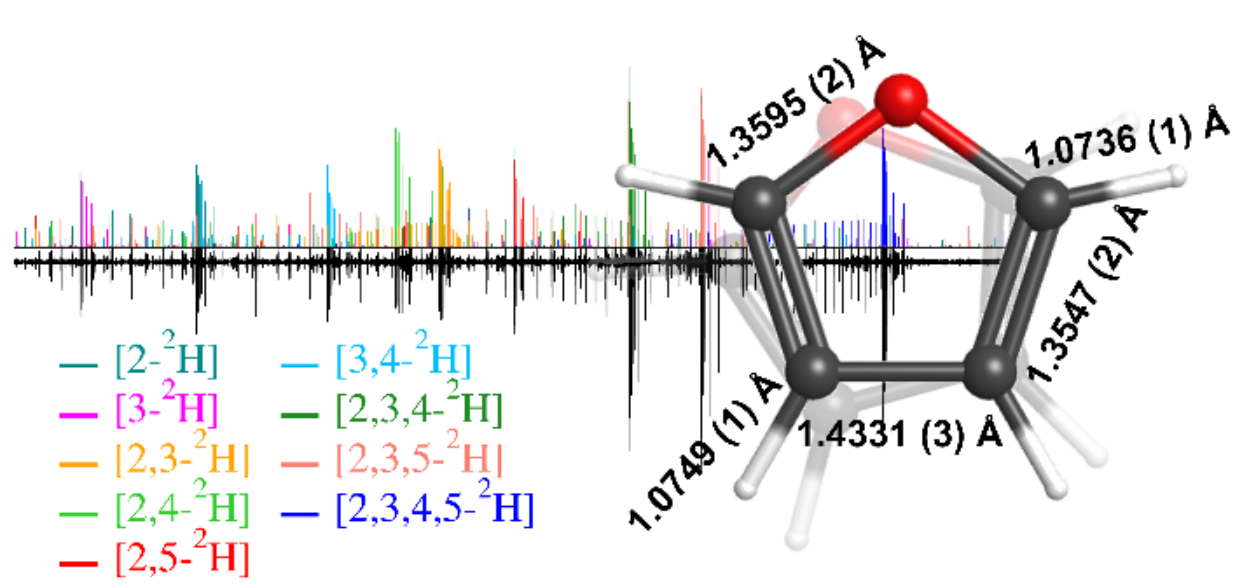

Table of Contents		Page
**Eq. S1–S6**	Equations for calculating the determinable rotational constants	S3
**Eq. S7–S15**	Equations for calculating the best theoretical estimate	S4
**Table S1.**	Corrections (ΔR) applied to CCSD(T)/cc-pCV5Z computed structural parameters to afford the Best Theoretical Estimate (BTE) for furan	S6
**Table S2.**	Numbering scheme for isotopologues and estimated dipole moments	S7
**Table S3.**	Spectroscopic constants of furan isotopologues (A-reduced Hamiltonian, I^r representation)	S8
**Table S4.**	Spectroscopic constants of furan isotopologues (S-reduced Hamiltonian, III^r representation)	S17
**Table S5.**	Determinable constants calculated from A- and S-reduction rotational constants and agreement between them for furan isotopologues	S26
**Figures S1-S44.**	Data distribution plots of furan isotopologues	S31
r_e vs. r_e^{SE} principal axis rotation analysis		S75
**Table S6.**	Rotational constant residuals ($B_e^{xrefit} - B_e^{\mathrm{SE}}$)	S76
Relation of the observable ground vibrational state and equilibrium rotational constants for an asymmetric-top molecule		S77
**Table S7.**	Rotational constant residuals ($B_e^{xrefit} - B_e^{\mathrm{SE}}$) vs ($B_e^{\mathrm{CCSD(T)}} - B_e^{\mathrm{SE}}$)	S78
**Figure S45.**	*xrefiteration* plots of structural parameter uncertainty values (δr_e^{SE}) as a function of number of isotopologues	S79
Furan-d_x synthetic details		S80
**Figure S46.**	Photograph of vessel used for synthesis	S81
**References**		S82

Additional Files Provided (zipped file)	
Computational outputs	Separate files
Isotopologue Fitting Files (*furan_###_A_I.res*, *furan_###_S_III.res)*	
r_e^{SE}(initial) structure outputs (*furan_reSE*.out*)	Separate files
r_e^{SE}(final) structure outputs (*furan_reSE_*final.out*)	Separate files
Xrefit output without computational corrections (provides $\Delta_{i\,0}$) (*furan_r0.out*)	Separate file
Xrefit outputs without electron-mass distribution correction (provides $\Delta_{i\,\mathrm{e}}$ using vibration-rotation interaction correction only) (*furan_reSE_*sans_e.out*)	Separate files
Files containing calculation of center-of-mass coordinates (*furan_pac_*_pac.out*)	Separate files
xrefiteration results (results for multiple structures included as separate sheets)	Separate files

Calculating the Determinable Rotational Constants

The ground-state rotational constants for both the S- and A-reduced Hamiltonians were converted to the "determinable constants" (A_0'', B_0'', C_0'') to account for the influence of centrifugal distortion using eq. (S1) – (S6).[1] The constants from each Hamiltonian reduction were then averaged to obtain the final determinable constants.

$$A_0'' = A_0^{(A)} + 2\Delta_J \quad \text{(S1)}$$

$$B_0'' = B_0^{(A)} + 2\Delta_J + \Delta_{JK} - 2\delta_J - 2\delta_K \quad \text{(S2)}$$

$$C_0'' = C_0^{(A)} + 2\Delta_J + \Delta_{JK} + 2\delta_J + 2\delta_K \quad \text{(S3)}$$

$$A_0'' = A_0^{(S)} + 2D_J + 6d_2 \quad \text{(S4)}$$

$$B_0'' = B_0^{(S)} + 2D_J + D_{JK} + 2d_1 + 4d_2 \quad \text{(S5)}$$

$$C_0'' = C_0^{(S)} + 2D_J + D_{JK} - 2d_1 + 4d_2 \quad \text{(S6)}$$

Ideally, the B_0'' values as determined from $B_0^{(A)}$ and $B_0^{(S)}$ are equal. The magnitude of the difference, if any, informs us as to the quality of the determinable constants. The differences between the B_0'' values for most isotopologues are well within two times the statistical uncertainties of the respective $B_0^{(A)}$ and $B_0^{(S)}$ values, and all of them are comparable in magnitude. This agreement provides confidence that both models employed in the least-squares fits are adequately treating the datasets and that the values obtained are suitable for use in the r_e^{SE} structure determination. See Table S5, below.

Calculating the Best Theoretical Estimate (BTE)

A "best theoretical estimate" (BTE) for the structure of furan uses the CCSD(T)/cc-pCV5Z structure as a basis, with four corrections that are evaluated using conventional methodology:[2,3]

1. Residual basis set effects beyond cc-pCV5Z: To estimate the correction needed to approach the infinite basis set limit, equilibrium structural parameters obtained with the cc-pCVXZ (X = T, Q, and 5) basis sets were extrapolated using the empirical exponential expression in Eq. (S7).[2-4]

$$R(x) = R(\infty) + Ae^{-Bx} \tag{S7}$$

R(x) are the values of the parameters obtained using the various basis sets (x = 3, 4, and 5), and R(∞) is the desired basis set limit estimate. Using three basis sets (x = 3, 4, and 5), the system of equations using Eq. (S7) can be solved, yielding Eq. (S8).[2]

$$R(\infty) = -\frac{R(4)^2 - R(3)R(5)}{R(3) + R(5) - 2R(4)} \tag{S8}$$

An alternate two-point extrapolation can be determined with Eq. S9 and Eq. S10.[5-8]

$$R(x) = R(\infty) + A\left(x + \tfrac{1}{2}\right)^{-4} \tag{S9}$$

$$R(\infty) = \frac{5.5^4 R(5) - 4.5^4 R(4)}{5.5^4 - 4.5^4} \tag{S10}$$

Three-point extrapolation (S8) was used for all parameters except $\theta_{C2-C3-H}$. Two-point extrapolation (S10) was used for $\theta_{C2-C3-H}$ because the computed values for this parameter displayed anomalous convergence behavior for the series cc-pCVXZ, X = T, Q, 5.

The correction to the structure due to a finite basis set is estimated by Eq. (S11).

$$\Delta R(\text{basis}) = R(\infty) - R(\text{CCSD(T)/cc-pCV5Z}) \tag{S11}$$

2. Residual electron correlation effects beyond the CCSD(T) treatment: Residual correlation effects are assessed by doing geometry optimizations at the CCSDT(Q) level[9] and then estimating the correlation correction in Eq. (S12).

$$\Delta R(\text{corr}) = R(\text{CCSDT(Q)}) - R(\text{CCSD(T)}) \tag{S12}$$

As calculations at the CCSDT(Q) level of theory are computationally demanding, these two calculations are obtained with the cc-pVDZ basis, in the frozen-core approximation.

3. <u>Effects of scalar (mass-velocity and Darwin) relativistic effects</u>: The relativistic corrections are obtained by subtraction of the equilibrium parameters obtained with a standard non-relativistic calculation from those obtained with the X2C-1e variant of coupled-cluster theory,[10-12] as shown in Eq. (S13).

$$\Delta R(\text{rel}) = R(\text{CCSD(T)/cc-pCVTZ})_{\text{SFX2C-1e}} - R(\text{CCSD(T)/cc-pCVTZ})_{NR} \qquad \text{(S13)}$$

The relativistic and non-relativistic calculations were performed with the cc-pCVTZ basis set. All electrons were correlated in both calculations. A re-contracted basis set was not used for the relativistic calculation.[13,14] (Keyword: RELATIVISTIC=X2C1E)

4. <u>The diagonal Born-Oppenheimer correction (DBOC)</u>: The diagonal Born-Oppenheimer correction (DBOC)[15,16] is obtained from Eq. (S14).

$$\Delta R(\text{DBOC}) = R(\text{SCF/cc-pVTZ})_{DBOC} - R(\text{SCF/cc-pVTZ})_{NR} \qquad \text{(S14)}$$

Here, the first value is obtained by minimizing the DBOC-corrected SCF energy with respect to nuclear positions, and the latter is again the traditional calculation. (Keyword: DBOC=ON)

(For more detail concerning corrections for non-Born-Oppenheimer behavior, see the following references: [2,17,18])

The sum of the above-described corrections is used to obtain the best equilibrium structural parameters, given by Eq. (S15).

$$\Delta R(\text{total}) = \Delta R(\text{basis}) + \Delta R(\text{corr}) + \Delta R(\text{rel}) + \Delta R(\text{DBOC}) \qquad \text{(S15)}$$

The sum of the corrections, *ΔR*(total), is applied to the CCSD(T)/cc-pCV5Z structural parameters to afford the BTE structure.

Table S1. Corrections (ΔR) applied to CCSD(T)/cc-pCV5Z computed structural parameters to afford the Best Theoretical Estimate (BTE) for furan. [a]

	ΔR(basis)	ΔR(corr)	ΔR(rel)	ΔR(DBOC)	ΔR(total)
$R_{O\text{-}C2}$ (Å)	−0.00007	0.00137	0.00005	0.00005	0.00139
$R_{C2\text{-}C3}$ (Å)	−0.00025	0.00076	−0.00029	0.00002	0.00025
$R_{C2\text{-}H}$ (Å)	−0.00004	0.00007	−0.00012	0.00015	0.00006
$R_{C3\text{-}H}$ (Å)	−0.00005	0.00012	−0.00012	0.00014	0.00009
$\theta_{O\text{-}C2\text{-}C3}$ (°)	−0.0120	0.0065	0.0081	−0.0001	0.0026
$\theta_{O\text{-}C2\text{-}H}$ (°)	0.0004	−0.0307	−0.0204	0.0004	−0.0503
$\theta_{C2\text{-}C3\text{-}H}$ (°)	−0.0152	−0.0058	−0.0021	−0.0009	−0.0240
$\theta_{C2\text{-}O\text{-}C5}$ (°)	0.0069	−0.0445	−0.0223	0.0004	−0.0594

Distance (R) in Ångstrom (Å); angle (θ) in degree (°).

[a] Exact values were used for all mathematical functions, but values in table are rounded to one decimal more than provided in the structural determination for legibility, resulting in apparent discrepancies between sum of rounded corrections shown and rounded ΔR(total).

Table S2. Numbering scheme for furan isotopologues and estimated dipole moments. [a]

Isotopologue	Isotopologue #	μ_a (D)	μ_b (D)
furan	001	0.70	0.00
[^{18}O]	002	0.70	0.00
[2-^{13}C]	003	0.56	0.42
[3-^{13}C]	004	0.66	0.23
[2-^{2}H]	005	0.33	0.62
[2-^{2}H, 2-^{13}C]	007	0.23	0.66
[2-^{2}H, 3-^{13}C]	008	0.29	0.64
[2-^{2}H, 4-^{13}C]	009	0.40	0.57
[2-^{2}H, 5-^{13}C]	010	0.30	0.63
[3-^{2}H]	011	0.60	0.36
[3-^{2}H, 2-^{13}C]	013	0.58	0.39
[3-^{2}H, 3-^{13}C]	014	0.64	0.29
[3-^{2}H, 4-^{13}C]	015	0.60	0.37
[3-^{2}H, 5-^{13}C]	016	0.56	0.42
[2,3-^{2}H]	017	0.47	0.52
[2,3-^{2}H, 2-^{13}C]	019	0.43	0.55
[2,3-^{2}H, 3-^{13}C]	020	0.48	0.51
[2,3-^{2}H, 4-^{13}C]	021	0.49	0.50
[2,3-^{2}H, 5-^{13}C]	022	0.44	0.54
[2,4-^{2}H]	023	0.40	0.58
[2,4-^{2}H, 2-^{13}C]	025	0.31	0.63
[2,4-^{2}H, 3-^{13}C]	026	0.48	0.51
[2,4-^{2}H, 4-^{13}C]	027	0.19	0.67
[2,4-^{2}H, 5-^{13}C]	028	0.19	0.67
[2,5-^{2}H]	029	0.00	0.70
[2,5-^{2}H, 2-^{13}C]	031	0.03	0.70
[2,5-^{2}H, 3-^{13}C]	032	0.09	0.69
[3,4-^{2}H]	033	0.70	0.00
[3,4-^{2}H, 2-^{13}C]	035	0.69	0.09
[3,4-^{2}H, 3-^{13}C]	036	0.69	0.10
[2,3,4-^{2}H]	037	0.54	0.45
[2,3,4-^{2}H, 2-^{13}C]	039	0.45	0.53
[2,3,4-^{2}H, 3-^{13}C]	040	0.59	0.37
[2,3,4-^{2}H, 4-^{13}C]	041	0.54	0.44
[2,3,4-^{2}H, 5-^{13}C]	042	0.48	0.51
[2,3,5-^{2}H]	043	0.30	0.63
[2,3,5-^{2}H, 2-^{13}C]	045	0.24	0.66
[2,3,5-^{2}H, 3-^{13}C]	046	0.26	0.65
[2,3,5-^{2}H, 4-^{13}C]	047	0.35	0.61
[2,3,5-^{2}H, 5-^{13}C]	048	0.28	0.64
[2,3,4,5-^{2}H]	049	0.00	0.70
[2,3,4,5-^{2}H, ^{18}O]	050	0.00	0.70
[2,3,4,5-^{2}H, 2-^{13}C]	051	0.07	0.70
[2,3,4,5-^{2}H, 3-^{13}C]	052	0.17	0.68

[a] Dipole moments determined from calculation of center-of-mass coordinates of isotopologues.

Table S3. Spectroscopic constants of furan isotopologues (A-reduced Hamiltonian, I^r representation).

	Furan [a]	[^{18}O] [a]	[2-^{13}C] [a]	[3-^{13}C] [a]	[2-^{2}H]
A_0 (MHz)	9447.120 653 (29)	9447.826 0 (15)	9295.555 621 (87)	9403.888 03 (12)	9280.340 810 (74)
B_0 (MHz)	9246.745 723 (28)	8841.878 5 (12)	9178.383 265 (87)	9043.829 03 (10)	8638.626 183 (61)
C_0 (MHz)	4670.824 577 (30)	4565.352 17 (16)	4616.222 278 (71)	4608.097 329 (72)	4472.126 904 (52)
Δ_J (kHz)	1.749 299 (13)	1.652 2 (21)	1.818 749 (55)	1.727 225 (73)	1.538 760 (38)
Δ_{JK} (kHz)	−0.249 163 (27)	−0.174 (10)	−1.064 64 (14)	−0.539 64 (21)	−0.465 22 (12)
Δ_K (kHz)	1.850 066 (34)	1.848 (23)	2.627 85 (20)	2.200 42 (33)	2.221 49 (15)
δ_J (kHz)	0.698 106 0 (29)	0.654 9 (10)	0.736 929 (26)	0.691 426 (35)	0.611 623 (18)
δ_K (kHz)	1.319 101 (14)	1.342 1 (65)	1.027 143 (75)	1.210 22 (11)	1.121 676 (54)
Φ_J (Hz)	0.000 740 8 (18)	[0.000 678]	0.000 677 (15)	0.000 644 (24)	0.000 538 4 (96)
Φ_{JK} (Hz)	0.000 292 1 (88)	[0.000 293 2]	[0.000 887 2]	[0.000 984 6]	[−0.000 029 5]
Φ_{KJ} (Hz)	−0.005 339 (22)	[−0.005 128]	−0.007 43 (25)	−0.007 57 (41)	−0.003 92 (13)
Φ_K (Hz)	0.005 386 (22)	[0.005 209 7]	0.007 09 (34)	0.007 55 (58)	0.005 22 (15)
φ_J (Hz)	0.000 372 12 (57)	[0.000 339 8]	0.000 338 1 (74)	0.000 314 (12)	0.000 276 6 (48)
φ_{JK} (Hz)	0.000 794 1 (49)	[0.000 772 8]	0.000 862 (34)	0.000 812 (49)	0.000 377 (24)
φ_K (Hz)	0.002 910 4 (75)	[0.002 98]	0.002 36 (12)	0.002 33 (20)	0.002 777 (56)
N_{lines} (rot) [b]	4344	296	1723	1546	2077
σ_{fit} (MHz)	0.027	0.042	0.035	0.035	0.029
κ [c]	0.916 096	0.751 787	0.949 919	0.849 844	0.733 076
Δ_i (μÅ^2) [d]	0.048 742 (1)	0.049 809 (12)	0.049 228 (2)	0.049 364 (2)	0.047 222 (1)

[a] Includes transitions from previous works.[19-23]

[b] Number of independent transition frequencies.

[c] $\kappa = (2B - A - C)/(A - C)$ calculated from B_0 values using PLANM.[24]

[d] Inertial defect ($\Delta_i = I_c - I_a - I_b$) calculated from B_0 values using PLANM.

	[2-^{2}H, 2-^{13}C]	[2-^{2}H, 3-^{13}C]	[2-^{2}H, 4-^{13}C]	[2-^{2}H, 5-^{13}C]	[3-^{2}H]
A_0 (MHz)	9274.729 (19)	9044.365 (24)	9242.974 (98)	9194.688 (20)	9383.705 859 (80)
B_0 (MHz)	8468.626 (17)	8633.649 (23)	8448.152 (87)	8517.703 (19)	8490.525 365 (67)
C_0 (MHz)	4424.838 87 (41)	4415.284 37 (58)	4412.016 02 (59)	4419.787 90 (53)	4455.482 434 (57)
Δ_J (kHz)	1.475 58 (56)	1.511 71 (18)	1.479 09 (18)	1.467 34 (63)	1.493 492 (45)
Δ_{JK} (kHz)	[−0.414 137]	[−0.525 11]	−0.546 7 (29)	[−0.251 404]	−0.327 90 (16)
Δ_K (kHz)	2.293 8 (80)	2.193 7 (84)	2.427 (11)	2.009 (10)	2.262 31 (24)
δ_J (kHz)	0.582 95 (30)	[0.601 667]	[0.585 798]	0.579 45 (34)	0.589 487 (20)
δ_K (kHz)	[1.102 9]	[1.024 81]	[1.048 73]	[1.145 73]	1.179 524 (77)
Φ_J (Hz)	[0.000 532 5]	[0.000 518 9]	[0.000 575 6]	[0.000 458 4]	0.000 531 (12)
Φ_{JK} (Hz)	[0.000 014 2]	[0.000 153]	[−0.000 308 4]	[0.000 624 3]	[0.000 316 8]
Φ_{KJ} (Hz)	[−0.003 797 8]	[−0.004 134 5]	[−0.003 472 2]	[−0.004 467 4]	−0.005 26 (25)
Φ_K (Hz)	[0.004 882 3]	[0.005 111 3]	[0.004 857]	[0.004 926 3]	0.006 37 (37)
φ_J (Hz)	[0.000 266 8]	[0.000 259 8]	[0.000 288 1]	[0.000 229 8]	0.000 269 4 (57)
φ_{JK} (Hz)	[0.000 453]	[0.000 474 7]	[0.000 351]	[0.000 645 1]	0.000 539 (39)
φ_K (Hz)	[0.002 714 7]	[0.002 263 9]	[0.002 728 5]	[0.002 514 4]	0.002 75 (11)
N_{lines} (rot) [b]	64	56	52	48	1980
σ_{fit} (MHz)	0.041	0.048	0.042	0.039	0.030
κ [c]	0.667 579	0.822 550	0.670 946	0.716 491	0.637 524
Δ_i (μÅ^2) [d]	0.047 59 (16)	0.047 51 (22)	0.047 70 (85)	0.047 61 (18)	0.048 789 (2)

	[3-^{2}H, 2-^{13}C]	[3-^{2}H, 3-^{13}C]	[3-^{2}H, 4-^{13}C]	[3-^{2}H, 5-^{13}C]	[2,3-^{2}H]
A_0 (MHz)	9183.740 (23)	9382.620 (27)	9177.899 (17)	9294.812 (29)	8686.964 389 (64)
B_0 (MHz)	8478.916 (20)	8297.391 (21)	8456.106 (15)	8374.315 (22)	8426.331 870 (61)
C_0 (MHz)	4406.739 55 (46)	4401.464 77 (58)	4399.235 91 (44)	4403.407 68 (90)	4275.616 577 (55)
Δ_J (kHz)	1.472 18 (16)	1.432 27 (74)	1.437 73 (15)	1.444 30 (41)	1.467 879 (44)
Δ_{JK} (kHz)	−0.453 7 (23)	[−0.315 304]	−0.201 6 (20)	−0.381 9 (52)	−0.701 92 (15)
Δ_K (kHz)	2.406 0 (76)	2.374 (14)	2.032 3 (69)	2.437 (20)	2.039 98 (18)
δ_J (kHz)	[0.582 177]	0.561 86 (40)	[0.565 375]	[0.568 205]	0.592 978 (20)
δ_K (kHz)	[1.070 32]	[1.154 68]	[1.142 47]	[1.114 63]	0.928 956 (51)
Φ_J (Hz)	[0.000 579 6]	[0.000 570 1]	[0.000 549 8]	[0.000 575]	0.000 546 (14)
Φ_{JK} (Hz)	[0.000 004 7]	[0.000 194 4]	[0.000 619]	[0.000 035 7]	−0.000 368 (55)
Φ_{KJ} (Hz)	[−0.004 257 9]	[−0.004 837 1]	[−0.005 562 4]	[−0.004 274 4]	−0.002 51 (19)
Φ_K (Hz)	[0.005 227 7]	[0.005 620 5]	[0.005 788 8]	[0.005 184 5]	0.003 73 (24)
φ_J (Hz)	[0.000 29]	[0.000 285]	[0.000 274 9]	[0.000 287 6]	0.000 273 8 (67)
φ_{JK} (Hz)	[0.000 521 8]	[0.000 635]	[0.000 809 1]	[0.000 551 2]	[0.000 169 5]
φ_K (Hz)	[0.002 619 8]	[0.002 857 6]	[0.002 409 3]	[0.002 825 3]	0.002 412 (78)
N_{lines} (rot) [b]	54	60	49	30	2238
σ_{fit} (MHz)	0.039	0.048	0.042	0.041	0.029
κ [c]	0.704 909	0.564 266	0.697 910	0.623 627	0.881 835
Δ_i (μÅ^2) [d]	0.049 24 (20)	0.049 14 (22)	0.049 05 (15)	0.049 12 (23)	0.047 396 (2)

	[2,3-^{2}H, 2-^{13}C]	[2,3-^{2}H, 3-^{13}C]	[2,3-^{2}H, 4-^{13}C]	[2,3-^{2}H, 5-^{13}C]	[2,4-^{2}H]
A_0 (MHz)	8611.043 (15)	8669.456 (10)	8475.901 (16)	8677.607 1 (64)	9204.500 197 (62)
B_0 (MHz)	8333.093 (14)	8253.198 0 (98)	8416.839 (17)	8242.793 8 (61)	7970.081 437 (52)
C_0 (MHz)	4233.198 33 (34)	4226.414 87 (30)	4221.439 88 (35)	4225.614 87 (30)	4269.766 158 (45)
Δ_J (kHz)	1.394 52 (10)	1.412 245 (86)	1.430 06 (37)	1.390 77 (32)	1.287 128 (27)
Δ_{JK} (kHz)	−0.413 8 (13)	−0.722 5 (13)	[−0.628 447]	[−0.584 687]	−0.229 083 (54)
Δ_K (kHz)	1.773 3 (37)	2.187 3 (36)	1.914 8 (37)	2.058 0 (46)	2.239 574 (68)
δ_J (kHz)	[0.559 027]	[0.567 842]	0.577 52 (21)	0.557 66 (17)	0.502 596 (11)
δ_K (kHz)	[0.980 192]	[0.888 624]	[0.888 314]	[0.931 011]	1.086 211 (36)
Φ_J (Hz)	[0.000 420 1]	[0.000 5819]	[0.000 344 6]	[0.000 509 7]	0.000 452 6 (47)
Φ_{JK} (Hz)	[0.000 719]	[−0.000 748]	[0.002 454 8]	[−0.000 169 7]	[−0.000 047 9]
Φ_{KJ} (Hz)	[−0.004 611 1]	[−0.002 111 3]	[−0.010 620 6]	[−0.003 129 3]	−0.003 344 (29)
Φ_K (Hz)	[0.004 812 8]	[0.003 576 9]	[0.009 67]	[0.004 175 4]	0.004 552 (34)
φ_J (Hz)	[0.000 210 2]	[0.000 291]	[0.000 172 3]	[0.000 255]	0.000 229 2 (23)
φ_{JK} (Hz)	[0.000 640 3]	[0.000 137 5]	[0.001 371 1]	[0.000 330 4]	0.000 417 (12)
φ_K (Hz)	[0.001 705 5]	[0.002 328 9]	[−0.000 187 6]	[0.002 079 2]	0.002 807 (14)
N_{lines} (rot) [b]	107	120	106	102	2790
σ_{fit} (MHz)	0.044	0.042	0.043	0.040	0.030
κ [c]	0.873 020	0.812 625	0.972 235	0.804 666	0.499 702
Δ_i (μÅ^2) [d]	0.047 80 (15)	0.047 764 (99)	0.048 01 (17)	0.047 868 (63)	0.047 062 (1)

	[2,4-^{2}H, 2-^{13}C]	[2,4-^{2}H, 3-^{13}C]	[2,4-2H, 4-^{13}C]	[2,4-^{2}H, 5-^{13}C]	[2,5-^{2}H]
A_0 (MHz)	9171.551 4 (32)	8973.228 (19)	9196.875 (18)	9053.390 (17)	9033.599 404 (82)
B_0 (MHz)	7837.825 4 (29)	7968.142 (15)	7796.561 (14)	7920.873 (14)	8160.782 273 (71)
C_0 (MHz)	4224.522 95 (33)	4218.758 06 (38)	4217.836 98 (40)	4222.999 19 (40)	4285.859 694 (74)
Δ_J (kHz)	1.239 159 (99)	1.267 12 (11)	1.232 93 (12)	1.255 16 (14)	1.318 789 (51)
Δ_{JK} (kHz)	−0.221 6 (16)	−0.317 2 (16)	−0.220 7 (18)	−0.252 4 (19)	0.002 39 (13)
Δ_K (kHz)	2.370 3 (68)	2.292 3 (59)	2.386 7 (82)	2.275 9 (64)	1.611 80 (10)
δ_J (kHz)	[0.481 242]	[0.495 927]	[0.478 35]	[0.489 692]	0.518 007 (18)
δ_K (kHz)	[1.051 61]	[0.984 414]	[1.055 48]	[1.023 05]	1.170 579 (49)
Φ_J (Hz)	[0.000 457 9]	[0.000 473 3]	[0.000 461 7]	[0.000 445]	0.000 312 (12)
Φ_{JK} (Hz)	[−0.000 063 5]	[−0.000 183 4]	[−0.000 064 4]	[−0.000 005 2]	0.001 377 (57)
Φ_{KJ} (Hz)	[−0.003 239 3]	[−0.003 014 8]	[−0.003 358 9]	[−0.003 189 4]	−0.005 404 (80)
Φ_K (Hz)	[0.004 463 5]	[0.004 309 8]	[0.004 597 4]	[0.004 373 4]	0.005 147 (44)
φ_J (Hz)	[0.000 229]	[0.000 236 7]	[0.000 230 7]	[0.000 222 6]	0.000 171 5 (46)
φ_{JK} (Hz)	[0.000 420 4]	[0.000 362 4]	[0.000 429 1]	[0.000 412 5]	0.000 760 (20)
φ_K (Hz)	[0.002 767 6]	[0.002 528 8]	[0.002 786 9]	[0.002 599 7]	0.002 330 (17)
N_{lines} (rot) ^{b}	100	100	108	90	1934
σ_{fit} (MHz)	0.044	0.047	0.044	0.042	0.029
κ ^{c}	0.460 797	0.577 204	0.437 516	0.531 087	0.632 323
Δ_i (μÅ^2) ^{d}	0.047 456 (32)	0.047 61 (17)	0.047 55 (16)	0.047 48 (15)	0.045 635 (2)

	[2,5-^{2}H, 2-^{13}C]	[2,5-^{2}H, 3-^{13}C]	[3,4-^{2}H] [a]	[3,4-^{2}H, 2-^{13}C]	[3,4-^{2}H, 3-^{13}C]
A_0 (MHz)	9022.522 9 (32)	8887.941 6 (16)	8819.595 085 (84)	8649.280 0 (38)	8757.181 (15)
B_0 (MHz)	8006.203 0 (31)	8082.197 4 (17)	8248.871 851 (75)	8223.700 6 (33)	8121.273 (14)
C_0 (MHz)	4240.377 04 (32)	4231.332 73 (30)	4260.585 197 (57)	4213.823 44 (30)	4211.883 97 (33)
Δ_J (kHz)	1.260 81 (11)	1.295 59 (65)	1.351 748 (44)	1.315 49 (10)	1.292 404 (95)
Δ_{JK} (kHz)	0.061 8 (13)	[0.006 342 32]	−0.136 96 (14)	−0.122 1 (13)	−0.041 0 (11)
Δ_K (kHz)	1.688 6 (51)	1.511 0 (41)	1.727 48 (25)	1.702 1 (47)	1.718 4 (45)
δ_J (kHz)	[0.491 745]	0.509 80 (33)	0.535 340 (19)	[0.520 43]	[0.508 565]
δ_K (kHz)	[1.151 9]	1.146 5 (14)	1.097 766 (72)	[1.041 67]	[1.083 1]
Φ_J (Hz)	[0.000 329 4]	[0.000 333 4]	0.000 563 (10)	[0.000 531]	[0.000 511 4]
Φ_{JK} (Hz)	[0.001 225 8]	[0.001 182 5]	[0.000 236 6]	[0.000 245 1]	[0.000 382 5]
Φ_{KJ} (Hz)	[−0.004 935 6]	[−0.004 761 6]	−0.004 26 (23)	[−0.003 914 7]	[−0.004 174 5]
Φ_K (Hz)	[0.004 719 6]	[0.004 524 3]	0.004 39 (34)	[0.004 143]	[0.004 302 7]
φ_J (Hz)	[0.000 165 1]	[0.000 167]	0.000 284 6 (48)	[0.000 265 4]	[0.000 255 5]
φ_{JK} (Hz)	[0.000 831]	[0.000 807]	0.000 628 (32)	[0.000 634 6]	[0.000 689 8]
φ_K (Hz)	[0.002 214 7]	[0.002 088 4]	0.002 41 (11)	[0.002 139 6]	[0.002 273 6]
N_{lines} (rot) [b]	120	142	1686	94	111
σ_{fit} (MHz)	0.039	0.043	0.029	0.038	0.039
κ [c]	0.574 952	0.653 935	0.749 628	0.808 101	0.720 191
Δ_i (μÅ^2) [d]	0.046 084 (33)	0.046 212 (19)	0.048 998 (2)	0.049 451 (37)	0.049 55 (15)

	[2,3,4-^{2}H]	[2,3,4-^{2}H, 2-^{13}C]	[2,3,4-^{2}H, 3-^{13}C]	[2,3,4-^{2}H, 4-^{13}C]	[2,3,4-^{2}H, 5-^{13}C]
A_0 (MHz)	8429.256 052 (46)	8393.786 4 (66)	8257.885 5 (93)	8429.349 (13)	8280.612 (12)
B_0 (MHz)	7957.234 010 (41)	7835.020 7 (59)	7943.533 4 (91)	7781.200 (12)	7920.483 (12)
C_0 (MHz)	4091.653 900 (41)	4050.842 92 (22)	4047.278 24 (21)	4044.612 11 (27)	4046.724 84 (22)
Δ_J (kHz)	1.262 509 (21)	1.220 376 (59)	1.226 698 (58)	1.206 259 (72)	1.239 224 (59)
Δ_{JK} (kHz)	−0.430 651 (68)	−0.431 28 (74)	−0.394 27 (73)	−0.397 30 (94)	−0.488 30 (71)
Δ_K (kHz)	1.828 08 (11)	1.911 3 (28)	1.801 6 (26)	1.920 6 (34)	1.872 9 (26)
δ_J (kHz)	0.504 979 2 (98)	[0.486 211]	[0.489 648]	[0.479 294]	[0.496 099]
δ_K (kHz)	0.894 839 (33)	[0.862 804]	[0.842 819]	[0.876 551]	[0.827 882]
Φ_J (Hz)	0.000 464 9 (39)	[0.000 460 9]	[0.000 473 2]	[0.000 466 3]	[0.000 451]
Φ_{JK} (Hz)	[−0.000 083 9]	[−0.000 128 8]	[−0.000 015]	[−0.000 108 6]	[−0.000 128 8]
Φ_{KJ} (Hz)	−0.002 994 (82)	[−0.002 803 5]	[−0.003 215]	[−0.003 016 5]	[−0.002 682 1]
Φ_K (Hz)	0.003 83 (12)	[0.003 631 1]	[0.003 839 4]	[0.003 827 8]	[0.003 534 6]
φ_J (Hz)	0.000 238 5 (19)	[0.000 230 4]	[0.000 236 4]	[0.000 232 9]	[0.000 225 4]
φ_{JK} (Hz)	0.000 353 (11)	[0.000 357 3]	[0.000 430 5]	[0.000 385 4]	[0.000 327 1]
φ_K (Hz)	0.001 979 (39)	[0.001 925 5]	[0.001 676 3]	[0.001 927 8]	[0.001 798 4]
N_{lines} (rot) ^{b}	2907	159	181	160	158
σ_{fit} (MHz)	0.027	0.037	0.038	0.040	0.035
κ ^{c}	0.782 358	0.742 679	0.850 686	0.704 361	0.829 883
Δ_i (μÅ^2) ^{d}	0.047 357 (1)	0.047 679 (68)	0.047 85 (10)	0.047 75 (14)	0.047 74 (13)

	[2,3,5-^{2}H]	[2,3,5-^{2}H, 2-^{13}C]	[2,3,5-^{2}H, 3-^{13}C]	[2,3,5-^{2}H, 4-^{13}C]	[2,3,5-^{2}H, 5-^{13}C]
A_0 (MHz)	8679.374 054 (47)	8608.269 5 (49)	8633.562 (12)	8474.081 6 (48)	8676.306 3 (28)
B_0 (MHz)	7779.608 852 (39)	7689.885 1 (44)	7646.630 (11)	7769.500 2 (40)	7626.677 4 (26)
C_0 (MHz)	4100.930 366 (39)	4060.100 70 (26)	4053.593 38 (22)	4051.753 22 (20)	4057.352 49 (24)
Δ_J (kHz)	1.200 283 (19)	1.153 35 (21)	1.158 744 (60)	1.177 492 (53)	1.148 765 (68)
Δ_{JK} (kHz)	−0.229 982 (55)	[−0.132 903]	−0.255 11 (93)	−0.261 30 (65)	-0.196 23 (90)
Δ_K (kHz)	1.802 963 (51)	1.760 9 (33)	1.930 2 (40)	1.802 0 (26)	1.904 2 (35)
δ_J (kHz)	0.473 473 4 (81)	0.452 39 (12)	[0.455 229]	[0.465 175]	[0.450 048]
δ_K (kHz)	0.979 327 (23)	[0.971 391]	[0.935 788]	[0.909 359]	[0.958 315]
Φ_J (Hz)	0.000 375 3 (34)	[0.000 341 2]	[0.000 389 7]	[0.000 366 8]	[0.000 373]
Φ_{JK} (Hz)	0.000 264 (20)	[0.000 478 2]	[0.000 053 9]	[0.000 260 3]	[0.000 197]
Φ_{KJ} (Hz)	−0.003 212 (30)	[−0.003 346 4]	[−0.002 771 2]	[−0.003 076 9]	[−0.003 0349]
Φ_K (Hz)	0.003 930 (18)	[0.003 852 8]	[0.003 670 6]	[0.003 768 6]	[0.003 84]
φ_J (Hz)	0.000 191 5 (16)	[0.000 170 7]	[0.000 194 8]	[0.000 183 4]	[0.000 186 6]
φ_{JK} (Hz)	0.000 424 0 (69)	[0.000 513 6]	[0.000 376 1]	[0.000 429 8]	[0.000 428 1]
φ_K (Hz)	0.002 225 0 (74)	[0.001 998 6]	[0.002 137 7]	[0.001 865 9]	[0.002 119 4]
N_{lines} (rot) [b]	3313	156	156	169	163
σ_{fit} (MHz)	0.027	0.036	0.034	0.035	0.037
κ [c]	0.606 956	0.596 152	0.569 022	0.681 353	0.545 512
Δ_i (μÅ^2) [d]	0.045 614 (1)	0.045 984 (51)	0.046 03 (13)	0.046 213 (48)	0.046 001 (30)

	[2,3,4,5-^{2}H]	[2,3,4,5-^{2}H, ^{18}O]	[2,3,4,5-^{2}H, 2-^{13}C]	[2,3,4,5-^{2}H, 3-^{13}C]
A_0 (MHz)	8056.503 508 (28)	7731.763 9 (13)	8046.051 17 (15)	7962.539 63 (15)
B_0 (MHz)	7688.059 234 (26)	7688.613 2 (16)	7548.379 25 (11)	7609.735 84 (15)
C_0 (MHz)	3932.587 022 (29)	3853.694 52 (20)	3893.256 785 (94)	3889.689 368 (93)
Δ_J (kHz)	1.153 438 9 (98)	1.133 555 (68)	1.117 403 (60)	1.136 03 (12)
Δ_{JK} (kHz)	−0.304 761 (22)	−0.393 16 (72)	−0.266 41 (25)	−0.323 81 (48)
Δ_K (kHz)	1.422 298 (26)	1.432 8 (22)	1.411 41 (26)	1.404 51 (46)
δ_J (kHz)	0.462 932 9 (42)	[0.456 306]	0.447 005 (18)	0.456 519 (56)
δ_K (kHz)	0.856 607 (10)	[0.743 732]	0.860 003 (88)	0.833 56 (15)
Φ_J (Hz)	0.000 324 5 (12)	[0.000 327 1]	0.000 326 (11)	0.000 357 (38)
Φ_{JK} (Hz)	0.000 485 0 (50)	[0.000 254 6]	0.000 484 (92)	[0.000 386 5]
Φ_{KJ} (Hz)	−0.003 162 3 (87)	[−0.002 553 9]	−0.003 39 (23)	−0.003 03 (51)
Φ_K (Hz)	0.003 399 7 (78)	[0.002 895 5]	0.003 54 (22)	0.003 12 (47)
φ_J (Hz)	0.000 166 07 (60)	[0.000 163 6]	[0.000 158]	0.000 187 (19)
φ_{JK} (Hz)	0.000 456 4 (24)	[0.000 366 3]	[0.000 473 8]	0.000 384 (68)
φ_K (Hz)	0.001 540 6 (26)	[0.001 265]	0.001 714 (86)	0.001 65 (18)
N_{lines} (rot) [b]	4694	148	1206	1203
σ_{fit} (MHz)	0.025	0.038	0.033	0.033
κ [c]	0.821 313	0.977 746	0.760 319	0.826 753
Δ_i (μÅ^2) [d]	0.045 673 (1)	0.046 590 (19)	0.046 003 (3)	0.046 121 (4)

Table S4. Spectroscopic constants of furan isotopologues (S-reduced Hamiltonian, III^r representation). [a]

	Furan [b]	[^{18}O] [b]	[2-^{13}C] [b]	[3-^{13}C] [b]	[2-^{2}H]
A_0 (MHz)	9447.122 669 (29)	9447.828 0 (15)	9295.558 050 (81)	9403.890 21 (11)	9280.342 933 (77)
B_0 (MHz)	9246.743 252 (28)	8841.875 9 (12)	9178.381 068 (81)	9043.826 653 (95)	8638.623 875 (62)
C_0 (MHz)	4670.824 931 (30)	4565.352 69 (16)	4616.222 083 (70)	4608.097 529 (72)	4472.127 111 (52)
D_J (kHz)	3.334 341 (16)	3.210 92 (88)	3.258 172 (73)	3.254 003 (97)	2.991 337 (51)
D_{JK} (kHz)	−5.263 773 (23)	−5.051 6 (14)	−5.145 65 (14)	−5.134 90 (19)	−4.711 439 (83)
D_K (kHz)	2.282 523 (18)	2.183 02 (62)	2.232 368 (84)	2.225 27 (11)	2.035 619 (54)
d_1 (kHz)	−0.051 173 4 (33)	−0.091 2 (42)	−0.022 378 (17)	−0.069 400 (32)	−0.133 251 (37)
d_2 (kHz)	0.043 246 8 (30)	0.033 4 (23)	−0.039 632 9 (93)	0.002 527 (15)	−0.018 603 (19)
H_J (Hz)	0.001 464 8 (23)	[0.001 341 1]	0.001 389 (19)	0.001 391 (28)	0.001 298 (14)
H_{JK} (Hz)	−0.006 098 7 (56)	[−0.005 566 5]	−0.005 954 (54)	−0.005 741 (87)	−0.005 253 (33)
H_{KJ} (Hz)	0.007 792 5 (75)	[0.007 093 9]	0.007 745 (65)	0.007 49 (10)	0.006 638 (40)
H_K (Hz)	−0.003 162 2 (37)	[−0.002 870 2]	−0.003 179 (30)	−0.003 119 (45)	−0.002 698 (21)
h_1 (Hz)	−0.000 087 0 (20)	[−0.000 060 4]	[−0.000 014 7]	[−0.000 043 3]	0.000 177 (18)
h_2 (Hz)	−0.000 094 3 (21)	[−0.000 067 9]	[0.000 015 5]	[−0.000 037 8]	0.000 085 (14)
h_3 (Hz)	−0.000 016 19 (57)	[−0.000 015 8]	0.000 035 2 (49)	0.000 049 9 (84)	[0.000 002 9]
N_{lines} (rot) [c]	4344	296	1723	1546	2073
σ_{fit} (MHz)	0.027	0.042	0.035	0.035	0.029
κ [d]	0.916 094	0.751 785	0.949 917	0.849 842	0.733 074
Δ_i (μÅ^2) [e]	0.048 730 (1)	0.049 791 (12)	0.049 234 (2)	0.049 357 (2)	0.047 214 (1)

[a] Left-handed outputs converted to right-handed for presentation.

[b] Includes transitions from previous works.[19-23]

[c] Number of independent transition frequencies.

[d] $\kappa = (2B - A - C)/(A - C)$ calculated from B_0 values using PLANM.[24]

[e] Inertial defect ($\Delta_i = I_c - I_a - I_b$) calculated from B_0 values using PLANM.

	[2-^{2}H, 2-^{13}C]	[2-^{2}H, 3-^{13}C]	[2-^{2}H, 4-^{13}C]	[2-^{2}H, 5-^{13}C]	[3-^{2}H]
A_0 (MHz)	9274.729 (19)	9044.370 (24)	9242.976 (98)	9194.689 (20)	9383.707 896 (69)
B_0 (MHz)	8468.625 (17)	8633.643 (24)	8448.151 (87)	8517.701 (19)	8490.523 008 (61)
C_0 (MHz)	4424.839 10 (42)	4415.284 45 (59)	4412.016 15 (60)	4419.788 22 (53)	4455.482 759 (57)
D_J (kHz)	2.929 2 (21)	2.903 1 (30)	2.906 9 (28)	2.914 1 (28)	3.004 632 (50)
D_{JK} (kHz)	−4.601 7 (45)	−4.576 1 (65)	−4.565 4 (60)	−4.584 7 (60)	−4.725 015 (86)
D_K (kHz)	1.982 2 (24)	1.981 4 (35)	1.966 0 (33)	1.979 1 (32)	2.034 901 (65)
d_1 (kHz)	[−0.147 716]	[−0.094 585 1]	[−0.150 891]	[−0.120 952]	−0.188 681 (21)
d_2 (kHz)	[−0.021 896 2]	[−0.015 055 9]	[−0.039 226 1]	[0.005 651 42]	−0.022 685 (11)
H_J (Hz)	[0.001 242 1]	[0.001 242 8]	[0.001 234 6]	[0.001 245 3]	0.001 246 (11)
H_{JK} (Hz)	[−0.004 958]	[−0.004 981 7]	[−0.004 930 5]	[−0.004 962 8]	−0.005 128 (24)
H_{KJ} (Hz)	[0.006 214 4]	[0.006 245 4]	[0.006 181 6]	[0.006 210 2]	0.006 560 (36)
H_K (Hz)	[−0.002 499 6]	[−0.002 507 3]	[−0.002 486 3]	[−0.002 493 8]	−0.002 686 (24)
h_1 (Hz)	[0.000 140 1]	[0.000 137 9]	[0.000 120 4]	[0.000 155 2]	[0.000 078 2]
h_2 (Hz)	[0.000 053 3]	[0.000 050 4]	[0.000 083 7]	[−0.000 007 7]	[0.000 034 5]
h_3 (Hz)	[0.000 001 2]	[0.000 014 6]	[0.000 004 6]	[0.000 000 7]	[0.000 015 4]
N_{lines} (rot) [c]	64	56	52	48	1980
σ_{fit} (MHz)	0.041	0.048	0.042	0.040	0.030
κ [d]	0.667 578	0.822 545	0.670 945	0.716 439	0.637 523
Δ_i (μÅ^2) [e]	0.047 57 (16)	0.047 50 (22)	0.047 70 (85)	0.047 60 (18)	0.048 776 (2)

	[3-^{2}H, 2-^{13}C]	[3-^{2}H, 3-^{13}C]	[3-^{2}H, 4-^{13}C]	[3-^{2}H, 5-^{13}C]	[2,3-^{2}H]
A_0 (MHz)	9183.742 (23)	9382.620 (27)	9177.901 (17)	9294.813 (29)	8686.966 352 (64)
B_0 (MHz)	8478.914 (20)	8297.390 (21)	8456.104 (15)	8374.314 (22)	8426.329 933 (61)
C_0 (MHz)	4406.739 68 (46)	4401.465 10 (58)	4399.236 21 (45)	4403.407 93 (91)	4275.616 585 (55)
D_J (kHz)	2.943 8 (18)	2.936 2 (34)	2.904 2 (20)	2.941 9 (57)	2.692 299 (43)
D_{JK} (kHz)	−4.647 7 (42)	−4.601 7 (72)	−4.566 8 (42)	−4.636 (12)	−4.262 396 (66)
D_K (kHz)	2.011 8 (24)	1.974 1 (38)	1.969 5 (22)	2.001 5 (61)	1.852 024 (47)
d_1 (kHz)	[−0.156 779]	[−0.209 358]	[−0.166 089]	[−0.187 188]	−0.038 045 (38)
d_2 (kHz)	[−0.029 343 6]	[−0.033 225 4]	[−0.005 651 3]	[−0.031 692 3]	−0.018 816 (11)
H_J (Hz)	[0.001 252]	[0.001 233 5]	[0.001 241 2]	[0.001 240 6]	0.001 170 9 (94)
H_{JK} (Hz)	[−0.005 029 2]	[−0.004 954 1]	[−0.005 006 4]	[−0.004 981]	−0.004 637 (18)
H_{KJ} (Hz)	[0.006 322]	[0.006 243]	[0.006 306 8]	[0.006 267 7]	0.005 840 (25)
H_K (Hz)	[−0.002 545 2]	[−0.002 522 3]	[−0.002 541 6]	[−0.002 527 5]	−0.002 375 (15)
h_1 (Hz)	[0.000 087 4]	[0.000 087 3]	[0.000 052 4]	[0.000 086 3]	0.000 101 (14)
h_2 (Hz)	[0.000 052 4]	[0.000 055 2]	[0.000 003 1]	[0.000 047 5]	[0.000 058 4]
h_3 (Hz)	[0.000 011 2]	[0.000 014 7]	[0.000 021 5]	[0.000 006 4]	[−0.000 005 7]
N_{lines} (rot) [c]	54	60	49	30	2238
σ_{fit} (MHz)	0.039	0.048	0.042	0.041	0.029
κ [d]	0.704 908	0.564 266	0.697 909	0.623 626	0.881 834
Δ_i (μÅ^2) [e]	0.049 24 (20)	0.049 13 (22)	0.049 04 (15)	0.049 11 (23)	0.047 396 (2)

	[2,3-^{2}H, 2-^{13}C]	[2,3-^{2}H, 3-^{13}C]	[2,3-^{2}H, 4-^{13}C]	[2,3-^{2}H, 5-^{13}C]	[2,4-^{2}H]
A_0 (MHz)	8611.035 (15)	8669.449 (10)	8475.893 (16)	8677.605 8 (65)	9204.502 358 (65)
B_0 (MHz)	8333.100 (14)	8253.205 0 (96)	8416.846 (17)	8242.794 1 (62)	7970.079 081 (52)
C_0 (MHz)	4233.198 43 (35)	4226.414 80 (30)	4221.439 87 (35)	4225.614 97 (30)	4269.766 563 (46)
D_J (kHz)	2.639 3 (10)	2.633 74 (92)	2.629 7 (12)	2.631 0 (13)	2.707 777 (40)
D_{JK} (kHz)	−4.176 3 (23)	−4.166 0 (21)	−4.167 0 (25)	−4.164 2 (28)	−4.229 172 (67)
D_K (kHz)	1.813 5 (13)	1.808 8 (12)	1.812 3 (14)	1.808 7 (15)	1.803 349 (44)
d_1 (kHz)	[−0.032 185 1]	[−0.055 669 5]	[−0.003 437 25]	[−0.057 796 1]	−0.251 370 (17)
d_2 (kHz)	[0.011 739 7]	[−0.031 367 8]	[−0.002 233 12]	[−0.016 049 8]	−0.043 616 7 (63)
H_J (Hz)	[0.001 108 7]	[0.001 100 4]	[0.001 098 8]	[0.001 102 8]	0.001 104 6 (79)
H_{JK} (Hz)	[−0.004 401 2]	[−0.004 377 6]	[−0.004 374 5]	[−0.004 368 5]	−0.004 356 (18)
H_{KJ} (Hz)	[0.005 480 7]	[0.005 458 5]	[0.005 453 3]	[0.005 436 1]	0.005 480 (24)
H_K (Hz)	[−0.002 188 5]	[−0.002 181 4]	[−0.002 177 6]	[−0.002 170 8]	−0.002 235 (13)
h_1 (Hz)	[0.000 104 4]	[0.000 042 1]	[0.000 132 1]	[0.000 085 9]	0.000 186 8 (42)
h_2 (Hz)	[−0.000 009]	[0.000 065 6]	[0.000 085 1]	[0.000 05]	0.000 085 1 (28)
h_3 (Hz)	[0.000 020 7]	[−0.000 008 2]	[0.000 157 8]	[0.000 005 7]	[0.000 004 7]
N_{lines} (rot) [c]	107	120	106	102	2790
σ_{fit} (MHz)	0.044	0.041	0.043	0.040	0.030
κ [d]	0.873 026	0.812 631	0.972 242	0.804 666	0.499 700
Δ_i (μÅ^2) [e]	0.047 80 (15)	0.047 771 (98)	0.048 00 (17)	0.047 859 (64)	0.047 045 (1)

	[2,4-^{2}H, 2-^{13}C]	[2,4-^{2}H, 3-^{13}C]	[2,4-2H, 4-^{13}C]	[2,4-^{2}H, 5-^{13}C]	[2,5-^{2}H]
A_0 (MHz)	9171.552 3 (32)	8973.224 (18)	9196.876 (18)	9053.386 (17)	9033.601 096 (82)
B_0 (MHz)	7837.823 8 (29)	7968.146 (15)	7796.560 (14)	7920.876 (14)	8160.780 014 (71)
C_0 (MHz)	4224.523 29 (34)	4218.758 20 (38)	4217.837 34 (40)	4222.999 45 (40)	4285.860 219 (74)
D_J (kHz)	2.658 2 (14)	2.638 6 (13)	2.652 2 (17)	2.644 5 (16)	2.685 037 (58)
D_{JK} (kHz)	−4.145 9 (32)	−4.137 0 (30)	−4.127 0 (38)	−4.139 8 (33)	−4.219 815 (84)
D_K (kHz)	1.764 3 (18)	1.773 7 (17)	1.751 1 (21)	1.771 1 (18)	1.817 557 (59)
d_1 (kHz)	[−0.255 751]	[−0.216 161]	[−0.264 36]	[−0.225 983]	−0.144 544 (24)
d_2 (kHz)	[−0.048 905 5]	[−0.044 300 4]	[−0.053 331 9]	[−0.040 751 6]	0.020 573 8 (96)
H_J (Hz)	[0.001 082 7]	[0.001 087 9]	[0.001 077 4]	[0.001 093 4]	0.001 115 (13)
H_{JK} (Hz)	[−0.004 201 9]	[−0.004 252 9]	[−0.004 176 3]	[−0.004 242 6]	−0.004 445 (24)
H_{KJ} (Hz)	[0.005 229 4]	[0.005 294 8]	[0.005 199 6]	[0.005 268 2]	0.005 620 (30)
H_K (Hz)	[−0.002 110 3]	[−0.002 129 8]	[−0.002 100 6]	[−0.002 119 1]	−0.002 320 (17)
h_1 (Hz)	[0.000 172]	[0.000 155 1]	[0.000 171]	[0.000 179 9]	0.000 185 0 (66)
h_2 (Hz)	[0.000 092 2]	[0.000 089]	[0.000 101]	[0.000 081 7]	−0.000 035 8 (37)
h_3 (Hz)	[0.000 003 6]	[0.000 004 5]	[0.000 007 1]	[0.000 003 5]	0.000 008 80 (82)
N_{lines} (rot) ^{c}	100	100	108	90	1934
σ_{fit} (MHz)	0.044	0.047	0.044	0.042	0.029
κ ^{d}	0.460 796	0.577 207	0.437 516	0.531 089	0.632 322
Δ_i (μÅ^2) ^{e}	0.047 438 (32)	0.047 61 (17)	0.047 54 (16)	0.047 48 (15)	0.045 614 (2)

	[2,5-^{2}H, 2-^{13}C]	[2,5-^{2}H, 3-^{13}C]	[3,4-^{2}H] [b]	[3,4-^{2}H, 2-^{13}C]	[3,4-^{2}H, 3-^{13}C]
A_0 (MHz)	9022.523 9 (32)	8887.943 0 (16)	8819.597 010 (75)	8649.280 7 (37)	8757.176 (15)
B_0 (MHz)	8006.201 3 (31)	8082.195 3 (17)	8248.869 616 (69)	8223.699 1 (33)	8121.277 (14)
C_0 (MHz)	4240.377 56 (32)	4231.333 26 (30)	4260.585 542 (58)	4213.823 72 (29)	4211.884 31 (33)
D_J (kHz)	2.634 0 (12)	2.616 4 (10)	2.708 169 (56)	2.646 1 (11)	2.653 8 (11)
D_{JK} (kHz)	−4.130 6 (27)	−4.114 8 (21)	−4.283 419 (92)	−4.191 8 (26)	-4.193 0 (25)
D_K (kHz)	1.774 0 (15)	1.774 4 (12)	1.856 329 (66)	1.820 3 (16)	1.814 4 (14)
d_1 (kHz)	[−0.154 205]	−0.123 2 (14)	−0.130 088 (18)	[−0.107 652]	[−0.137 79]
d_2 (kHz)	[0.019 727 3]	[0.024 583 1]	0.012 868 7 (94)	[0.016 873 7]	[0.014 551 2]
H_J (Hz)	[0.001 086 2]	[0.001 083]	0.001 135 (13)	[0.001 106]	[0.001 099 2]
H_{JK} (Hz)	[−0.004 225 3]	[−0.004 235]	−0.004 609 (25)	[−0.004 424 3]	[−0.004 405 6]
H_{KJ} (Hz)	[0.005 231 5]	[0.005 249 5]	0.005 862 (32)	[0.005 534 9]	[0.005 523 3]
H_K (Hz)	[−0.002 093 3]	[−0.002 098 1]	−0.002 393 (20)	[−0.002 216 4]	[−0.002 216 5]
h_1 (Hz)	[0.000 163 5]	[0.000 148 3]	[−0.000 001 1]	[−0.000 010 7]	[0.000 003 5]
h_2 (Hz)	[−0.000 043 4]	[−0.000 055 1]	[−0.000 035 1]	[−0.000 036 5]	[−0.000 038 5]
h_3 (Hz)	[0.000 006 4]	[0.000 004 5]	[−0.000 007 7]	[−0.000 003 6]	[−0.000 003 6]
N_{lines} (rot) [c]	120	142	1686	94	111
σ_{fit} (MHz)	0.039	0.043	0.029	0.037	0.039
κ [d]	0.574 951	0.653 934	0.749 627	0.808 100	0.720 194
Δ_i (μÅ^2) [e]	0.046 063 (33)	0.046 190 (19)	0.048 984 (2)	0.049 437 (36)	0.049 34 (15)

	[2,3,4-^{2}H]	[2,3,4-^{2}H, 2-^{13}C]	[2,3,4-^{2}H, 3-^{13}C]	[2,3,4-^{2}H, 4-^{13}C]	[2,3,4-^{2}H, 5-^{13}C]
A_0 (MHz)	8429.257 827 (46)	8393.786 3 (65)	8257.879 3 (94)	8429.345 (13)	8280.610 (12)
B_0 (MHz)	7957.232 157 (41)	7835.020 5 (58)	7943.539 0 (92)	7781.203 (12)	7920.486 (12)
C_0 (MHz)	4091.654 018 (41)	4050.843 01 (22)	4047.278 28 (22)	4044.612 22 (27)	4046.724 85 (22)
D_J (kHz)	2.435 378 (29)	2.387 49 (73)	2.386 12 (73)	2.384 31 (83)	2.377 94 (71)
D_{JK} (kHz)	−3.856 774 (50)	−3.777 6 (16)	−3.782 7 (16)	−3.768 0 (19)	−3.769 5 (16)
D_K (kHz)	1.673 948 (32)	1.638 03 (90)	1.644 02 (91)	1.631 4 (11)	1.638 53 (88)
d_1 (kHz)	0.096 867 (16)	[−0.100 463]	[−0.082 733 3]	[−0.115 669]	[−0.072 858 7]
d_2 (kHz)	0.015 408 2 (48)	[−0.020 737 6]	[−0.010 678 8]	[−0.022 903 2]	[−0.017 122 8]
H_J (Hz)	0.001 004 2 (52)	[0.000 971 1]	[0.000 969 3]	[0.000 964 8]	[0.000 972 8]
H_{JK} (Hz)	−0.003 983 (11)	[−0.003 814 5]	[−0.003 824 1]	[−0.003 792 7]	[−0.003 818 8]
H_{KJ} (Hz)	0.004 964 (14)	[0.004 728]	[0.004 746 8]	[0.004 706 2]	[0.004 727 9]
H_K (Hz)	−0.001 996 8 (77)	[−0.001 884 4]	[−0.001 891 7]	[−0.001 877 9]	[−0.001 881 7]
h_1 (Hz)	0.000 056 3 (31)	[0.000 056 1]	[0.000 022 9]	[0.000 051 6]	[0.000 064 2]
h_2 (Hz)	[0.000 031 9]	[0.000 034 8]	[0.000 022 5]	[0.000 042 8]	[0.000 032 8]
h_3 (Hz)	[0.000 007 3]	[0.000 003 4]	[0.000 011 2]	[0.000 007 6]	[0.000 004]
N_{lines} (rot) [c]	2907	159	181	160	158
σ_{fit} (MHz)	0.027	0.036	0.038	0.040	0.035
κ [d]	0.782 356	0.742 679	0.850 691	0.704 364	0.829 885
Δ_i (μÅ^2) [e]	0.047 352 (1)	0.047 690 (67)	0.047 85 (10)	0.047 75 (14)	0.047 75 (13)

	[2,3,5-^{2}H]	[2,3,5-^{2}H, 2-^{13}C]	[2,3,5-^{2}H, 3-^{13}C]	[2,3,5-^{2}H, 4-^{13}C]	[2,3,5-^{2}H, 5-^{13}C]
A_0 (MHz)	8679.375 824 (47)	8608.269 8 (49)	8633.561 (12)	8474.081 1 (48)	8676.306 9 (28)
B_0 (MHz)	7779.606 811 (39)	7689.884 2 (44)	7646.631 (11)	7769.500 0 (40)	7626.676 4 (26)
C_0 (MHz)	4100.930 682 (40)	4060.101 03 (26)	4053.593 64 (22)	4051.753 44 (21)	4057.352 79 (24)
D_J (kHz)	2.428 479 (27)	2.381 49 (83)	2.377 17 (93)	2.369 37 (68)	2.382 37 (83)
D_{JK} (kHz)	−3.819 219 (43)	−3.745 7 (18)	−3.733 3 (21)	−3.737 4 (15)	−3.737 4 (18)
D_K (kHz)	1.644 080 (29)	1.612 74 (96)	1.604 4 (11)	1.615 16 (82)	1.603 72 (98)
d_1 (kHz)	−0.156 536 3 (99)	[−0.149 199]	[−0.160 056]	[−0.123 086]	[−0.168 351]
d_2 (kHz)	−0.015 896 3 (43)	[−0.005 324 45]	[−0.023 662]	[−0.010 265 3]	[−0.020 222 5]
H_J (Hz)	0.000 991 5 (46)	[0.000 965 9]	[0.000 957]	[0.000 959 5]	[0.000 960 1]
H_{JK} (Hz)	−0.003 897 1 (98)	[−0.003 713]	[−0.003 686 8]	[−0.003 714 7]	[−0.003 684 8]
H_{KJ} (Hz)	0.004 866 (13)	[0.004 568 9]	[0.004 544 8]	[0.004 579 7]	[0.004 536 4]
H_K (Hz)	−0.001 967 9 (73)	[−0.001 822]	[−0.001 815]	[−0.001 824 5]	[−0.001 811 8]
h_1 (Hz)	0.000 156 9 (22)	[0.000 157 3]	[0.000 140 2]	[0.000 140 3]	[0.000 153 5]
h_2 (Hz)	0.000 036 8 (16)	[0.000 019 2]	[0.000 052]	[0.000 033 4]	[0.000 050 3]
h_3 (Hz)	[0.000 002 1]	[0.000 003 6]	[0.000 000 7]	[0.000 006]	[0.000 003 7]
N_{lines} (rot) [c]	3313	156	156	169	163
σ_{fit} (MHz)	0.027	0.037	0.034	0.036	0.037
κ [d]	0.606 954	0.596 151	0.569 023	0.681 353	0.545 517
Δ_i (μÅ^2) [e]	0.045 599 (1)	0.045 968 (51)	0.046 03 (13)	0.046 201 (48)	0.046 18 (39)

	[2,3,4,5-^{2}H]	[2,3,4,5-^{2}H, ^{18}O]	[2,3,4,5-^{2}H, 2-^{13}C]	[2,3,4,5-^{2}H, 3-^{13}C]
A_0 (MHz)	8056.504 961 (28)	7731.764 6 (13)	8046.052 67 (12)	7962.541 15 (13)
B_0 (MHz)	7688.057 555 (26)	7688.611 3 (17)	7548.377 56 (12)	7609.734 17 (13)
C_0 (MHz)	3932.587 224 (29)	3853.694 60 (20)	3893.257 013 (94)	3889.689 563 (92)
D_J (kHz)	2.193 173 (13)	2.113 18 (68)	2.150 58 (10)	2.148 366 (91)
D_{JK} (kHz)	−3.478 074 (23)	−3.347 7 (14)	−3.406 78 (19)	−3.407 11 (16)
D_K (kHz)	1.512 477 (15)	1.455 50 (79)	1.479 59 (12)	1.481 75 (11)
d_1 (kHz)	−0.047 918 3 (45)	[−0.008 543 06]	−0.062 793 (22)	−0.042 038 (30)
d_2 (kHz)	0.009 017 2 (21)	[0.008 732 44]	0.006 806 (12)	0.007 685 (28)
H_J (Hz)	0.000 878 1 (18)	[0.000 812]	0.000 850 (26)	0.000 852 (22)
H_{JK} (Hz)	−0.003 445 1 (44)	[−0.003 175 9]	−0.003 499 (69)	−0.003 358 (59)
H_{KJ} (Hz)	0.004 266 4 (53)	[0.003 915 9]	0.004 472 (86)	0.004 227 (80)
H_K (Hz)	−0.001 707 1 (26)	[−0.001 552]	−0.001 813 (40)	−0.001 737 (40)
h_1 (Hz)	0.000 092 5 (11)	[0.000 063 1]	[0.000 092 7]	[0.000 075 6]
h_2 (Hz)	−0.000 013 76 (80)	[−0.000 011 6]	[−0.000 014 6]	[−0.000 016 8]
h_3 (Hz)	0.000 004 68 (16)	[0.000 004 2]	[0.000 003 6]	[0.000 002 7]
N_{lines} (rot) ^{c}	4694	148	1206	1203
σ_{fit} (MHz)	0.025	0.038	0.033	0.033
κ ^{d}	0.821 312	0.977 745	0.760 318	0.826 752
Δ_i (μÅ^2) ^{e}	0.045 663 (1)	0.046 577 (19)	0.045 992 (3)	0.046 112 (3)

Table S5. Determinable constants calculated from A- and S-reduction rotational constants and agreement between them for furan isotopologues. [a]

Isotopologue	Furan	[^{18}O]	[2-^{13}C]	[3-^{13}C]	[2-^{2}H]
$A_0''^{(A)}$ (MHz)	9447.1241516	9447.8293044	9295.5592585	9403.8914845	9280.3438875
$B_0''^{(A)}$ (MHz)	9246.7449380	8841.8776364	9178.3823097	9043.8281415	8638.6253287
$C_0''^{(A)}$ (MHz)	4670.8318608	4565.3592944	4616.2283790	4608.1040471	4472.1329829
$A_0''^{(S)}$ (MHz)	9447.1295972	9447.8346222	9295.5643285	9403.8967332	9280.3488041
$B_0''^{(S)}$ (MHz)	9246.7447275	8841.8772214	9178.3822354	9043.8278974	8638.6248053
$C_0''^{(S)}$ (MHz)	4670.8266112	4565.3543762	4616.2233399	4608.0990510	4472.1285743
A_0'' Mean (MHz)	9447.1269	9447.8320	9295.5618	9403.8941	9280.3463
B_0'' Mean (MHz)	9246.74483	8841.87743	9178.382273	9043.82802	8638.62507
C_0'' Mean (MHz)	4670.8292	4565.3568	4616.2259	4608.1015	4472.1308
$A_0''^{(A)} - A_0''^{(S)}$ (MHz)	−0.0054	−0.0053	−0.0051	−0.0052	−0.0049
$B_0''^{(A)} - B_0''^{(S)}$ (MHz)	0.00021	0.00041	0.000074	0.00024	0.00052
$C_0''^{(A)} - C_0''^{(S)}$ (MHz)	0.0052	0.0049	0.0050	0.0050	0.0044

[a] Values were calculated from unrounded values; values in table are rounded for ease of reading.

Isotopologue	[2-^{2}H, 2-^{13}C]	[2-^{2}H, 3-^{13}C]	[2-^{2}H, 4-^{13}C]	[2-^{2}H, 5-^{13}C]	[3-^{2}H]
$A_0''^{(A)}$ (MHz)	9274.7319512	9044.3680234	9242.9769582	9194.6909347	9383.7088460
$B_0''^{(A)}$ (MHz)	8468.6251653	8633.6482454	8448.1511424	8517.7022329	8490.5244861
$C_0''^{(A)}$ (MHz)	4424.8447787	4415.2901213	4412.0217005	4419.7940336	4455.4886311
$A_0''^{(S)}$ (MHz)	9274.7347270	9044.3757159	9242.9815784	9194.6948621	9383.7137681
$B_0''^{(S)}$ (MHz)	8468.6258737	8633.6439807	8448.1517897	8517.7020242	8490.5238101
$C_0''^{(S)}$ (MHz)	4424.8405645	4415.2858090	4412.0175433	4419.7897280	4455.4843399
A_0'' Mean (MHz)	9274.7333	9044.3719	9242.9793	9194.6929	9383.7113
B_0'' Mean (MHz)	8468.62552	8633.6461	8448.15147	8517.70213	8490.52415
C_0'' Mean (MHz)	4424.8427	4415.2880	4412.0196	4419.7919	4455.4865
$A_0''^{(A)} - A_0''^{(S)}$ (MHz)	−0.0028	−0.0077	−0.0046	−0.0039	−0.0049
$B_0''^{(A)} - B_0''^{(S)}$ (MHz)	−0.00071	0.0043	−0.00065	0.00021	0.00068
$C_0''^{(A)} - C_0''^{(S)}$ (MHz)	0.0042	0.0043	0.0042	0.0043	0.0043

Isotopologue	[3-^{2}H, 2-^{13}C]	[3-^{2}H, 3-^{13}C]	[3-^{2}H, 4-^{13}C]	[3-^{2}H, 5-^{13}C]	[2,3-^{2}H]
$A_0''^{(A)}$ (MHz)	9183.7429444	9382.6228645	9177.9018755	9294.8148886	8686.9673248
$B_0''^{(A)}$ (MHz)	8478.9151857	8297.3901162	8456.1052582	8374.3141410	8426.3310600
$C_0''^{(A)}$ (MHz)	4406.7453457	4401.4707523	4399.2419996	4403.4135524	4275.6218547
$A_0''^{(S)}$ (MHz)	9183.7477115	9382.6256730	9177.9067745	9294.8186936	8686.9716237
$B_0''^{(S)}$ (MHz)	8478.9148090	8297.3907191	8456.1048868	8374.3147467	8426.3309038
$C_0''^{(S)}$ (MHz)	4406.7411161	4401.4666565	4399.2377612	4403.4094254	4275.6177080
A_0'' Mean (MHz)	9183.7429444	9382.6228645	9177.9018755	9294.8148886	8686.9673248
B_0'' Mean (MHz)	8478.9151857	8297.3901162	8456.1052582	8374.3141410	8426.3310600
C_0'' Mean (MHz)	4406.7453457	4401.4707523	4399.2419996	4403.4135524	4275.6218547
$A_0''^{(A)} - A_0''^{(S)}$ (MHz)	−0.0048	−0.0028	−0.0049	−0.0038	−0.0043
$B_0''^{(A)} - B_0''^{(S)}$ (MHz)	0.00038	−0.00060	0.00037	−0.00061	0.00016
$C_0''^{(A)} - C_0''^{(S)}$ (MHz)	0.0042	0.0041	0.0042	0.0041	0.0041

Isotopologue	[2,3-^{2}H, 2-^{13}C]	[2,3-^{2}H, 3-^{13}C]	[2,3-^{2}H, 4-^{13}C]	[2,3-^{2}H, 5-^{13}C]	[2,4-^{2}H]
$A_0''^{(A)}$ (MHz)	8611.0457890	8669.4542675	8475.9038601	8677.6098815	9204.5027713
$B_0''^{(A)}$ (MHz)	8333.0922968	8253.2060529	8416.8383000	8242.7930195	7970.0806046
$C_0''^{(A)}$ (MHz)	4233.2037837	4226.4159501	4221.4450433	4225.6200442	4269.7716808
$A_0''^{(S)}$ (MHz)	8611.0403490	8669.4641562	8475.9091900	8677.6109657	9204.5075119
$B_0''^{(S)}$ (MHz)	8333.1010849	8253.2025208	8416.8436299	8242.7950180	7970.0795902
$C_0''^{(S)}$ (MHz)	4233.1996436	4226.4216622	4221.4468200	4225.6161192	4269.7680777
A_0'' Mean (MHz)	8611.0431	8669.4592	8475.9065	8677.6104	9204.5051
B_0'' Mean (MHz)	8333.0967	8253.2043	8416.8410	8242.7940	7970.0801
C_0'' Mean (MHz)	4233.2017	4226.4188	4221.4459	4225.6181	4269.7699
$A_0''^{(A)} - A_0''^{(S)}$ (MHz)	0.0054	−0.0099	−0.0053	−0.0011	−0.0047
$B_0''^{(A)} - B_0''^{(S)}$ (MHz)	−0.0088	0.0035	−0.0053	−0.0020	0.0010
$C_0''^{(A)} - C_0''^{(S)}$ (MHz)	0.0041	−0.0057	−0.0018	0.0039	0.0036

Isotopologue	[2,4-^{2}H, 2-^{13}C]	[2,4-^{2}H, 3-^{13}C]	[2,4-2H, 4-^{13}C]	[2,4-^{2}H, 5-^{13}C]	[2,5-^{2}H]
$A_0''^{(A)}$ (MHz)	9171.5538783	8973.2305342	9196.8774659	9053.3925103	9033.6020416
$B_0''^{(A)}$ (MHz)	7837.8245910	7968.1412564	7796.5601775	7920.8722324	8160.7815358
$C_0''^{(A)}$ (MHz)	4224.5282724	4218.7632377	4217.8422928	4223.0044734	4285.8657111
$A_0''^{(S)}$ (MHz)	9171.5573230	8973.2290114	9196.8809844	9053.3910445	9033.6065895
$B_0''^{(S)}$ (MHz)	7837.8242634	7968.1465307	7796.5604354	7920.8765342	8160.7809575
$C_0''^{(S)}$ (MHz)	4224.5247764	4218.7595953	4217.8388328	4223.0008882	4285.8617406
A_0'' Mean (MHz)	9171.5556	8973.2298	9196.8792	9053.3918	9033.6043
B_0'' Mean (MHz)	7837.82443	7968.1439	7796.56031	7920.8744	8160.78125
C_0'' Mean (MHz)	4224.5265	4218.7614	4217.8406	4223.0027	4285.8637
$A_0''^{(A)} - A_0''^{(S)}$ (MHz)	−0.0034	0.0015	−0.0035	0.0015	−0.0045
$B_0''^{(A)} - B_0''^{(S)}$ (MHz)	0.00033	−0.0053	−0.00026	−0.0043	0.00058
$C_0''^{(A)} - C_0''^{(S)}$ (MHz)	0.0035	0.0036	0.0035	0.0036	0.0040

Isotopologue	[2,5-^{2}H, 2-^{13}C]	[2,5-^{2}H, 3-^{13}C]	[3,4-^{2}H]	[3,4-^{2}H, 2-^{13}C]	[3,4-^{2}H, 3-^{13}C]
$A_0''^{(A)}$ (MHz)	9022.5254216	8887.9441912	8819.5977885	8649.2826310	8757.1835848
$B_0''^{(A)}$ (MHz)	8006.2022961	8082.1966849	8248.8711513	8223.6999847	8121.2723605
$C_0''^{(A)}$ (MHz)	4240.3829107	4231.3386401	4260.5910297	4213.8290731	4211.8896971
$A_0''^{(S)}$ (MHz)	9022.5292864	8887.9483803	8819.6025036	8649.2860934	8757.1813949
$B_0''^{(S)}$ (MHz)	8006.2022079	8082.1962699	8248.8705402	8223.7000526	8121.2778972
$C_0''^{(S)}$ (MHz)	4240.3790847	4231.3347227	4260.5869866	4213.8251032	4211.8857584
A_0'' Mean (MHz)	9022.5274	8887.9463	8819.6001	8649.2844	8757.1825
B_0'' Mean (MHz)	8006.202252	8082.19648	8248.87085	8223.700019	8121.2751
C_0'' Mean (MHz)	4240.3810	4231.3367	4260.5890	4213.8271	4211.8877
$A_0''^{(A)} - A_0''^{(S)}$ (MHz)	−0.0039	−0.0042	−0.0047	−0.0035	0.0022
$B_0''^{(A)} - B_0''^{(S)}$ (MHz)	0.000088	0.00041	0.00061	−0.000068	−0.0055
$C_0''^{(A)} - C_0''^{(S)}$ (MHz)	0.0038	0.0039	0.0040	0.0040	0.0039

Isotopologue	[2,3,4-^{2}H]	[2,3,4-^{2}H, 2-^{13}C]	[2,3,4-^{2}H, 3-^{13}C]	[2,3,4-^{2}H, 4-^{13}C]	[2,3,4-^{2}H, 5-^{13}C]
$A_0''^{(A)}$ (MHz)	8429.2585770	8393.7888408	8257.8879534	8429.3514125	8280.6144784
$B_0''^{(A)}$ (MHz)	7957.2333047	7835.0200114	7943.5327942	7781.1993035	7920.4823422
$C_0''^{(A)}$ (MHz)	4091.6587940	4050.8476275	4047.2829641	4044.6168369	4046.7294781
$A_0''^{(S)}$ (MHz)	8429.2627902	8393.7909506	8257.8822078	8429.3496312	8280.6146531
$B_0''^{(S)}$ (MHz)	7957.2334263	7835.0212135	7943.5411813	7781.2036777	7920.4867722
$C_0''^{(S)}$ (MHz)	4091.6548999	4050.8441254	4047.2794123	4044.6133603	4046.7259136
A_0'' Mean (MHz)	8429.2585770	8393.7888408	8257.8879534	8429.3514125	8280.6144784
B_0'' Mean (MHz)	7957.2333047	7835.0200114	7943.5327942	7781.1993035	7920.4823422
C_0'' Mean (MHz)	4091.6587940	4050.8476275	4047.2829641	4044.6168369	4046.7294781
$A_0''^{(A)} - A_0''^{(S)}$ (MHz)	−0.0042	−0.0021	0.0057	0.0018	−0.00017
$B_0''^{(A)} - B_0''^{(S)}$ (MHz)	−0.00012	−0.0012	−0.0084	−0.0044	−0.0044
$C_0''^{(A)} - C_0''^{(S)}$ (MHz)	0.0039	0.0035	0.0036	0.0035	0.0036

Isotopologue	[2,3,5-^{2}H]	[2,3,5-^{2}H, 2-^{13}C]	[2,3,5-^{2}H, 3-^{13}C]	[2,3,5-^{2}H, 4-^{13}C]	[2,3,5-^{2}H, 5-^{13}C]
$A_0''^{(A)}$ (MHz)	8679.3764546	8608.2718067	8633.5643175	8474.0839550	8676.3085975
$B_0''^{(A)}$ (MHz)	7779.6081170	7689.8844262	7646.6292803	7769.4995446	7626.6766846
$C_0''^{(A)}$ (MHz)	4100.9354422	4060.1057214	4053.5982244	4051.7580628	4057.3574080
$A_0''^{(S)}$ (MHz)	8679.3805856	8608.2745310	8633.5656124	8474.0857771	8676.3115434
$B_0''^{(S)}$ (MHz)	7779.6074721	7689.8848976	7646.6316063	7769.5007141	7626.6770097
$C_0''^{(S)}$ (MHz)	4100.9319692	4060.1023244	4053.5948865	4051.7546465	4057.3540732
A_0'' Mean (MHz)	8679.3785	8608.2732	8633.5650	8474.0849	8676.3101
B_0'' Mean (MHz)	7779.60779	7689.88466	7646.6304	7769.5001	7626.67685
C_0'' Mean (MHz)	4100.9337	4060.1040	4053.5966	4051.7564	4057.3557
$A_0''^{(A)} - A_0''^{(S)}$ (MHz)	−0.0041	−0.0027	−0.0013	−0.0018	−0.0029
$B_0''^{(A)} - B_0''^{(S)}$ (MHz)	0.00064	−0.00047	−0.0023	−0.0012	−0.00033
$C_0''^{(A)} - C_0''^{(S)}$ (MHz)	0.0035	0.0034	0.0033	0.0034	0.0033

Isotopologue	[2,3,4,5-^{2}H]	[2,3,4,5-^{2}H, ^{18}O]	[2,3,4,5-^{2}H, 2-^{13}C]	[2,3,4,5-^{2}H, 3-^{13}C]
$A_0''^{(A)}$ (MHz)	8056.5058149	7731.7661671	8046.0534048	7962.5419021
$B_0''^{(A)}$ (MHz)	7688.0585970	7688.6126739	7548.3786044	7609.7352081
$C_0''^{(A)}$ (MHz)	3932.5916632	3853.6987940	3893.2613674	3889.6938964
$A_0''^{(S)}$ (MHz)	8056.5094014	7731.7688788	8046.0570120	7962.5454928
$B_0''^{(S)}$ (MHz)	7688.0584035	7688.6121965	7548.3783560	7609.7350063
$C_0''^{(S)}$ (MHz)	3932.5882642	3853.6955307	3893.2580602	3889.6905674
A_0'' Mean (MHz)	8056.5076	7731.7675	8046.0552	7962.5437
B_0'' Mean (MHz)	7688.05850	7688.61244	7548.37848	7609.73511
C_0'' Mean (MHz)	3932.5900	3853.6972	3893.2597	3889.6922
$A_0''^{(A)} - A_0''^{(S)}$ (MHz)	−0.0036	−0.0027	−0.0036	−0.0036
$B_0''^{(A)} - B_0''^{(S)}$ (MHz)	0.00019	0.00048	0.00025	0.00020
$C_0''^{(A)} - C_0''^{(S)}$ (MHz)	0.0034	0.0033	0.0033	0.0033

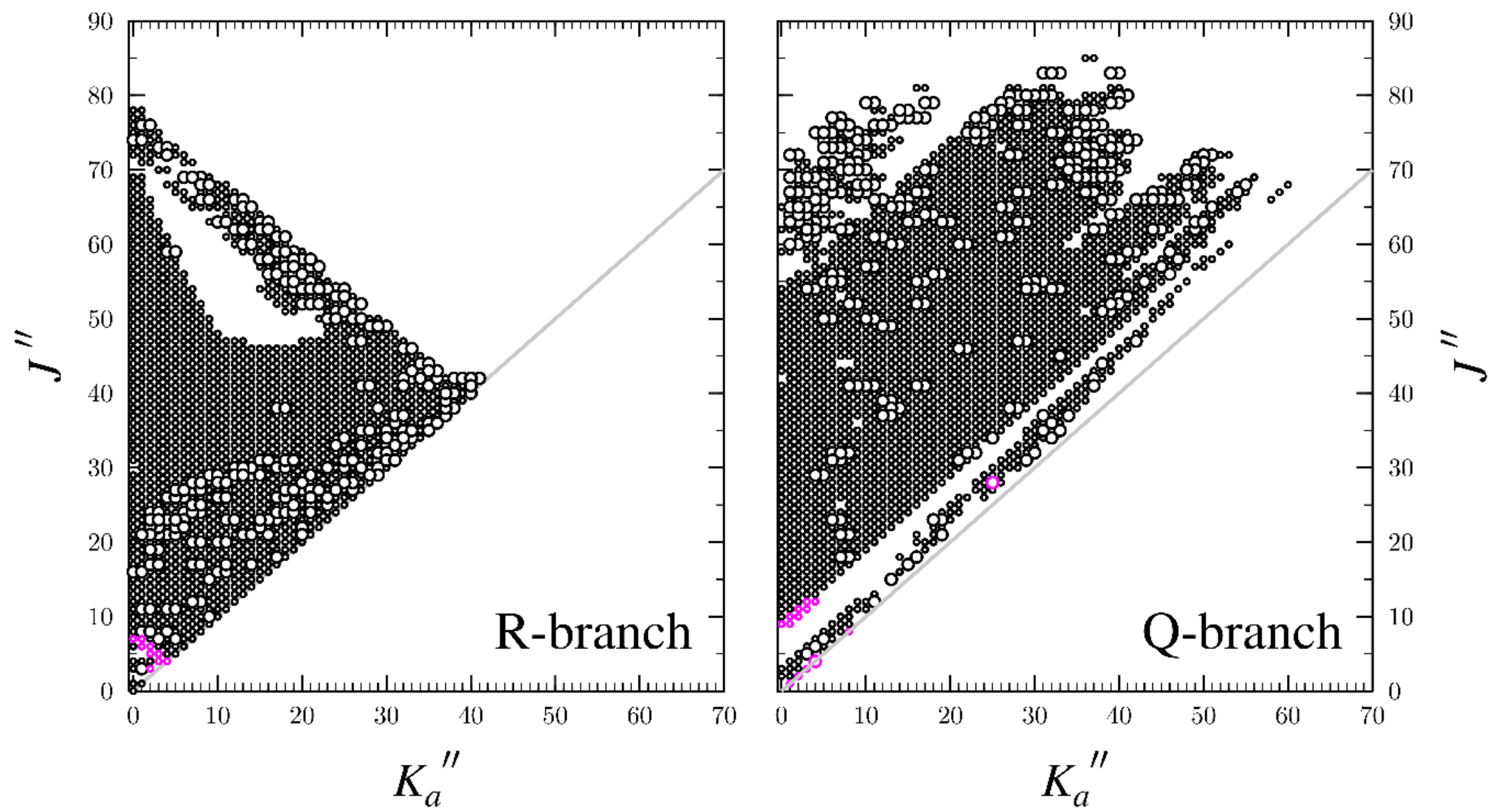

Fig. S1. Data distribution plot for the least-squares fit of spectroscopic data for the vibrational ground state, normal isotopologue of furan. Black circles are measurements from the current work, while pink circles are from prior works.[20,21,23] The size of the outlined circle is proportional to the value of $|(f_{obs.} - f_{calc.})/\delta f|$, where δf is the frequency measurement uncertainty, and all values represented are less than or equal to 3.

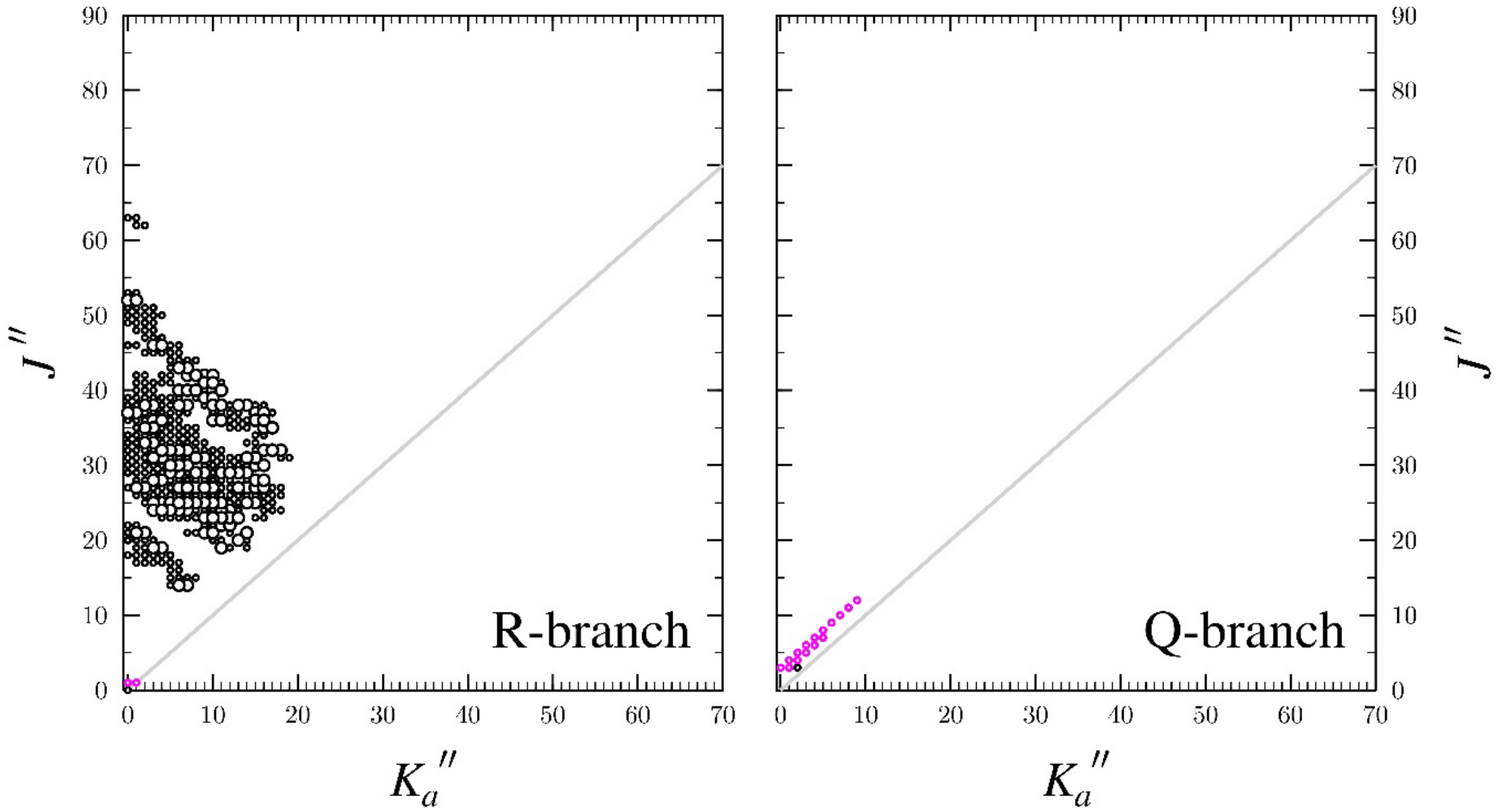

Fig. S2. Data distribution plot for the least-squares fit of spectroscopic data for the vibrational ground state, [^{18}O]-furan isotopologue. Black circles are measurements from the current work, while pink circles are from prior works.[19] The size of the outlined circle is proportional to the value of $|(f_{obs.} - f_{calc.})/\delta f|$, where δf is the frequency measurement uncertainty, and all values represented are less than or equal to 3.

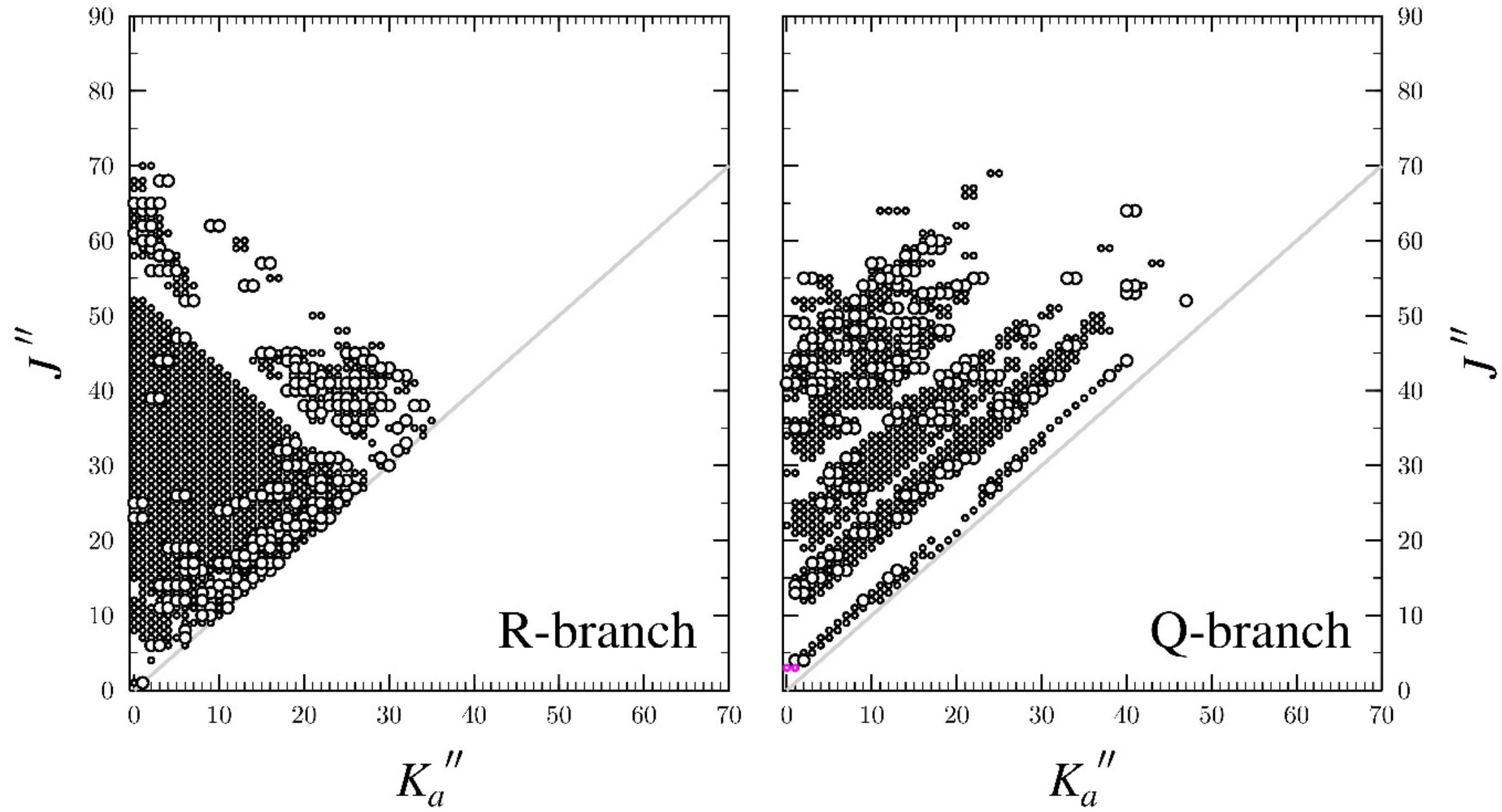

Fig. S3. Data distribution plot for the least-squares fit of spectroscopic data for the vibrational ground state, [2-^{13}C]-furan isotopologue. Black circles are measurements from the current work, while pink circles are from prior works.[19] The size of the outlined circle is proportional to the value of $|(f_{obs.} - f_{calc.})/\delta f|$, where δf is the frequency measurement uncertainty, and all values represented are less than or equal to 3.

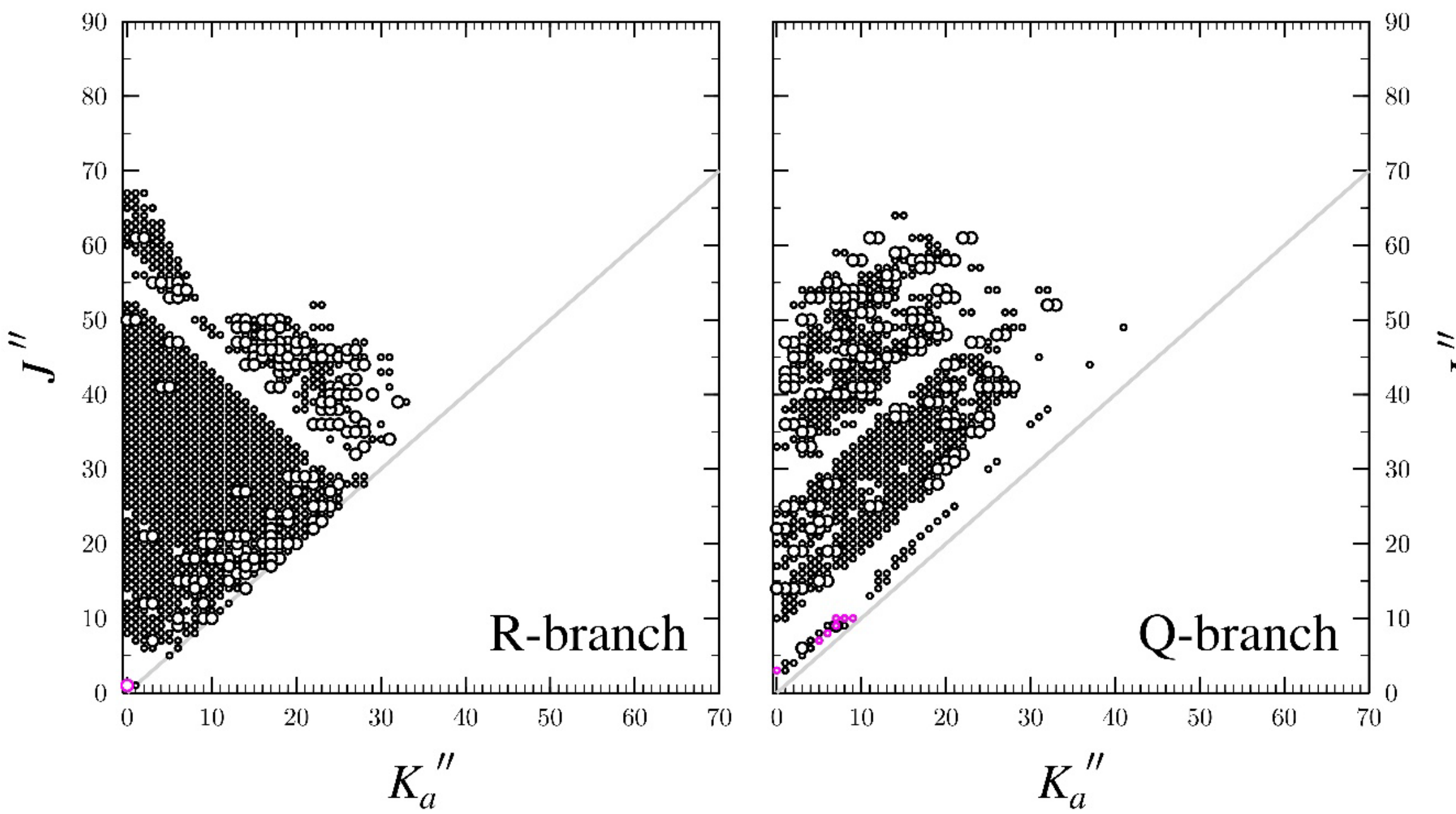

Fig. S4. Data distribution plot for the least-squares fit of spectroscopic data for the vibrational ground state, [3-^{13}C]-furan isotopologue. Black circles are measurements from the current work, while pink circles are from prior works.[19] The size of the outlined circle is proportional to the value of $|(f_{obs.} - f_{calc.})/\delta f|$, where δf is the frequency measurement uncertainty, and all values represented are less than or equal to 3.

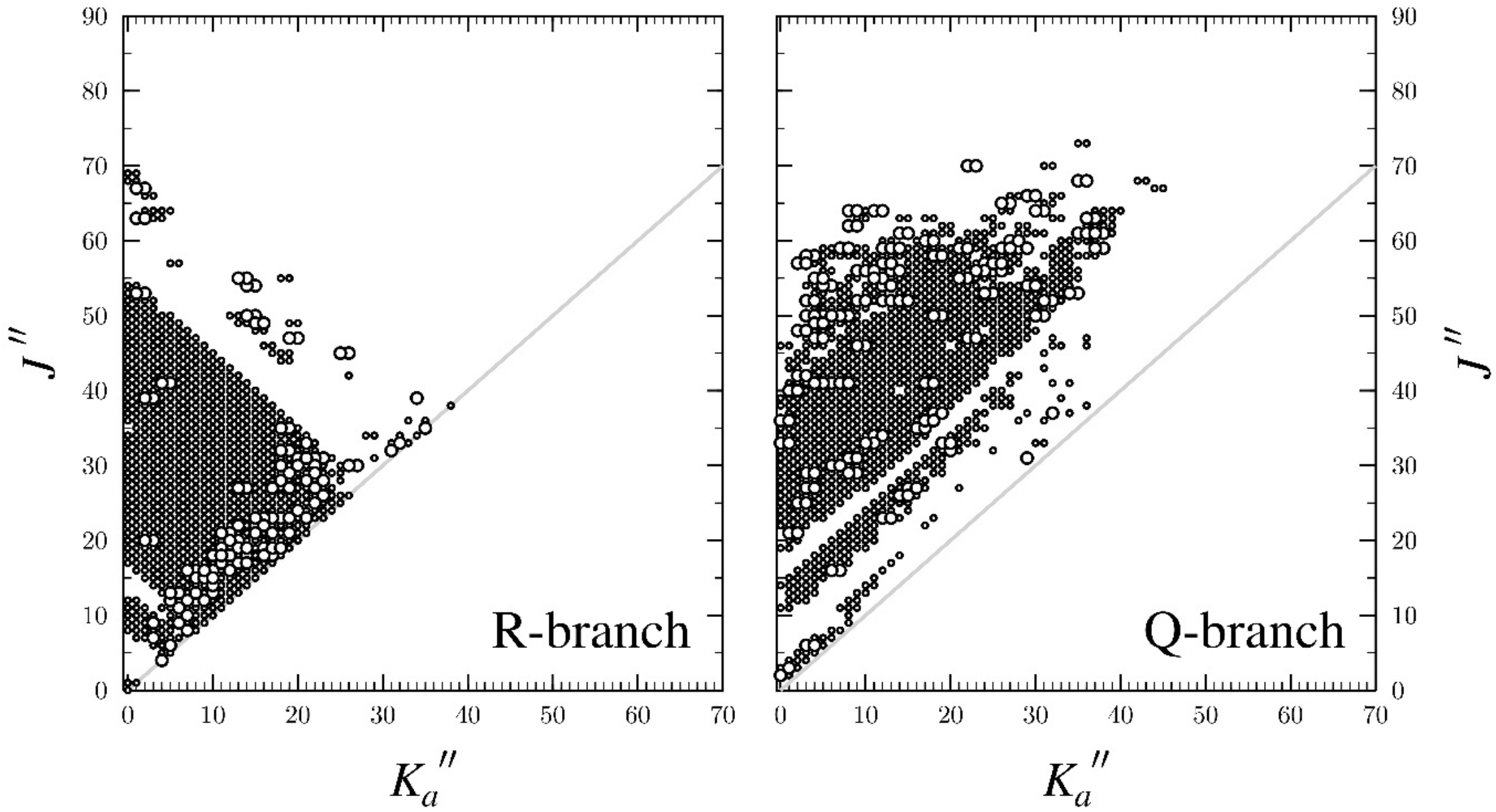

Fig. S5. Data distribution plot for the least-squares fit of spectroscopic data for the vibrational ground state, [2-^{2}H]-furan isotopologue. The size of the outlined circle is proportional to the value of $|(f_{obs.} - f_{calc.})/\delta f|$, where δf is the frequency measurement uncertainty, and all values represented are less than or equal to 3.

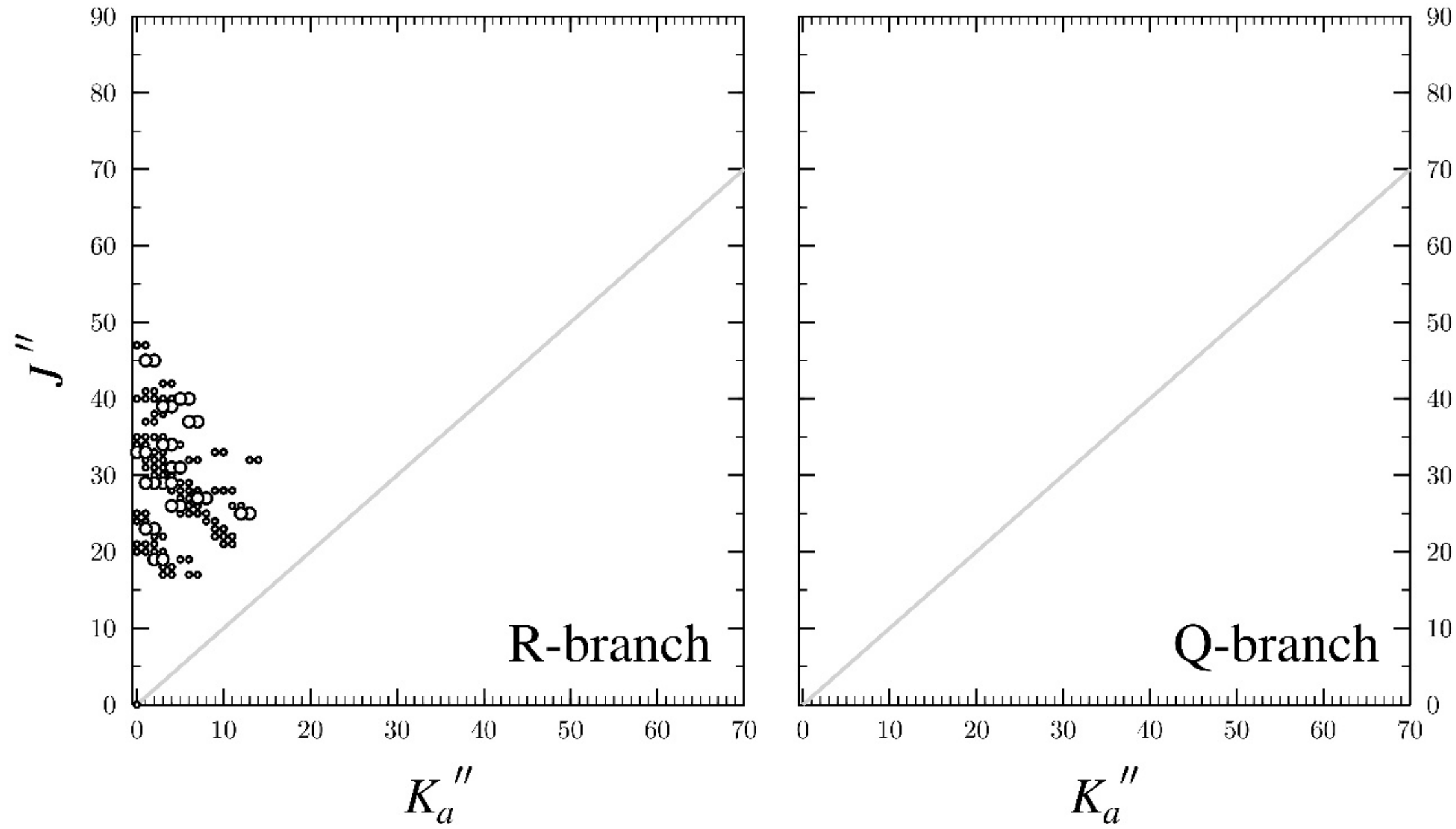

Fig. S6. Data distribution plot for the least-squares fit of spectroscopic data for the vibrational ground state, [2-^{2}H, 2-^{13}C]-furan isotopologue. The size of the outlined circle is proportional to the value of $|(f_{obs.} - f_{calc.})/\delta f|$, where δf is the frequency measurement uncertainty, and all values represented are less than or equal to 3.

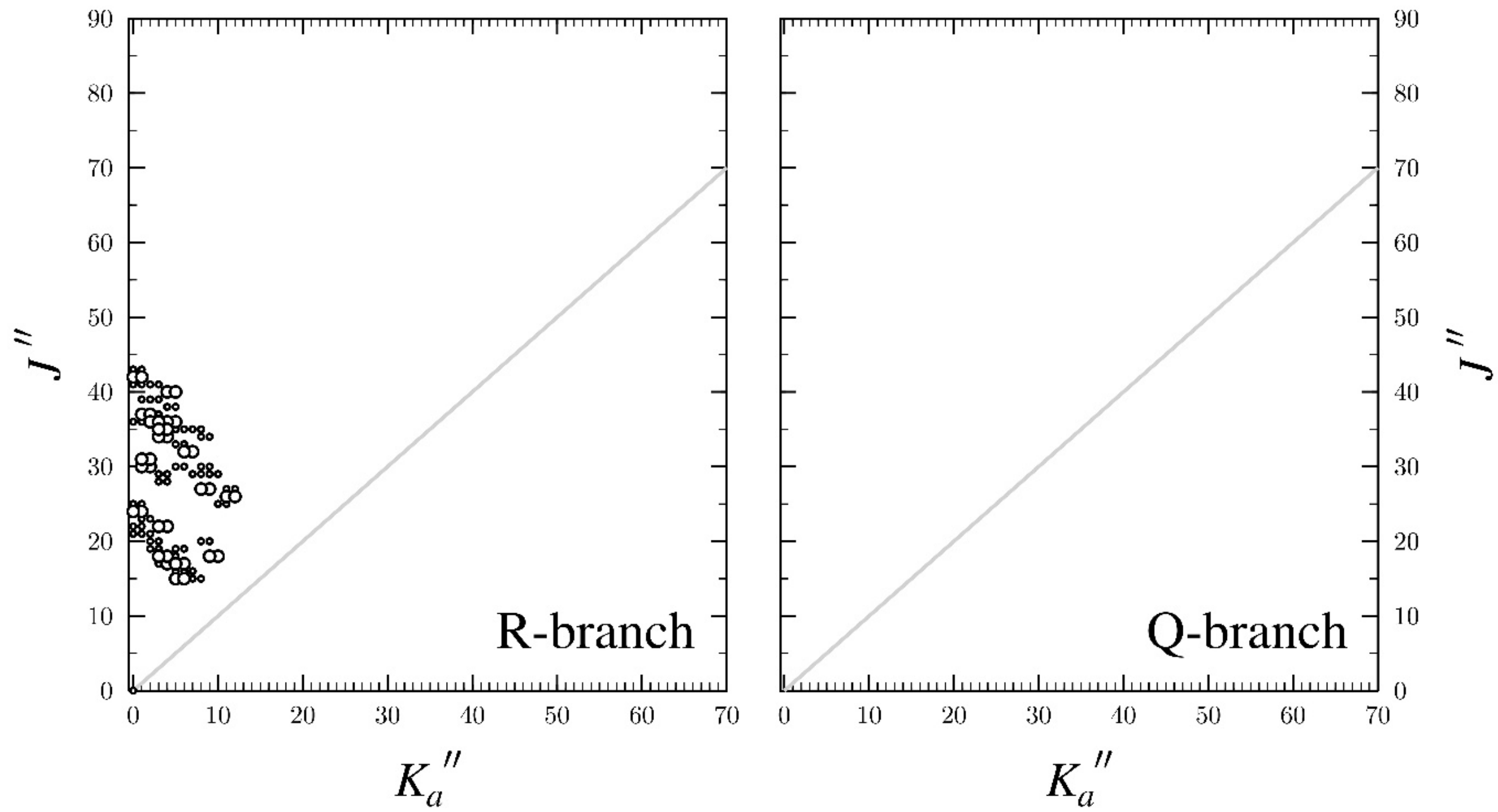

Fig. S7. Data distribution plot for the least-squares fit of spectroscopic data for the vibrational ground state, [2-^{2}H, 3-^{13}C]-furan isotopologue. The size of the outlined circle is proportional to the value of $|(f_{obs.} - f_{calc.})/\delta f|$, where δf is the frequency measurement uncertainty, and all values represented are less than or equal to 3.

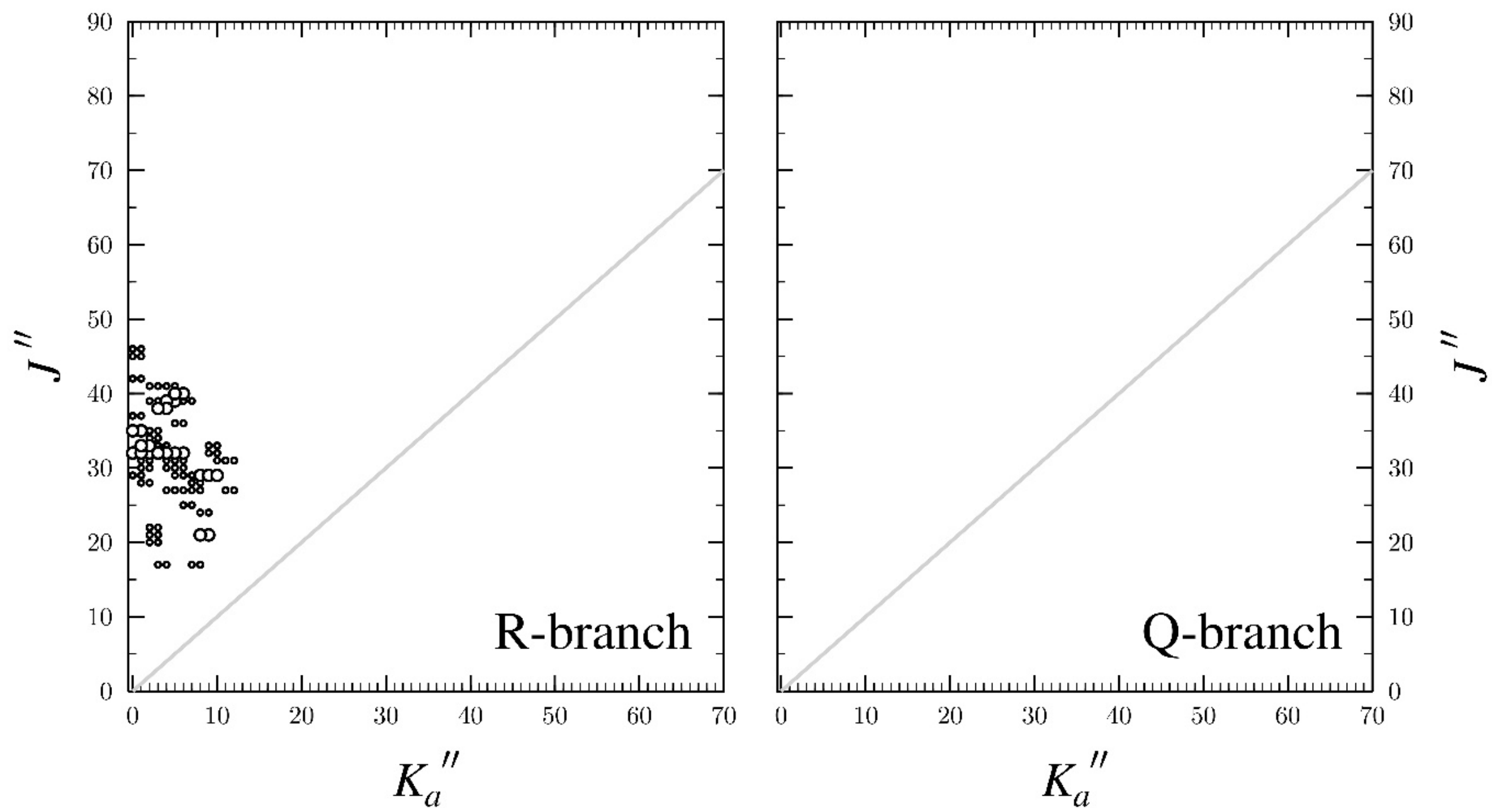

Fig. S8. Data distribution plot for the least-squares fit of spectroscopic data for the vibrational ground state, [2-^{2}H, 4-^{13}C]-furan isotopologue. The size of the outlined circle is proportional to the value of $|(f_{obs.} - f_{calc.})/\delta f|$, where δf is the frequency measurement uncertainty, and all values represented are less than or equal to 3.

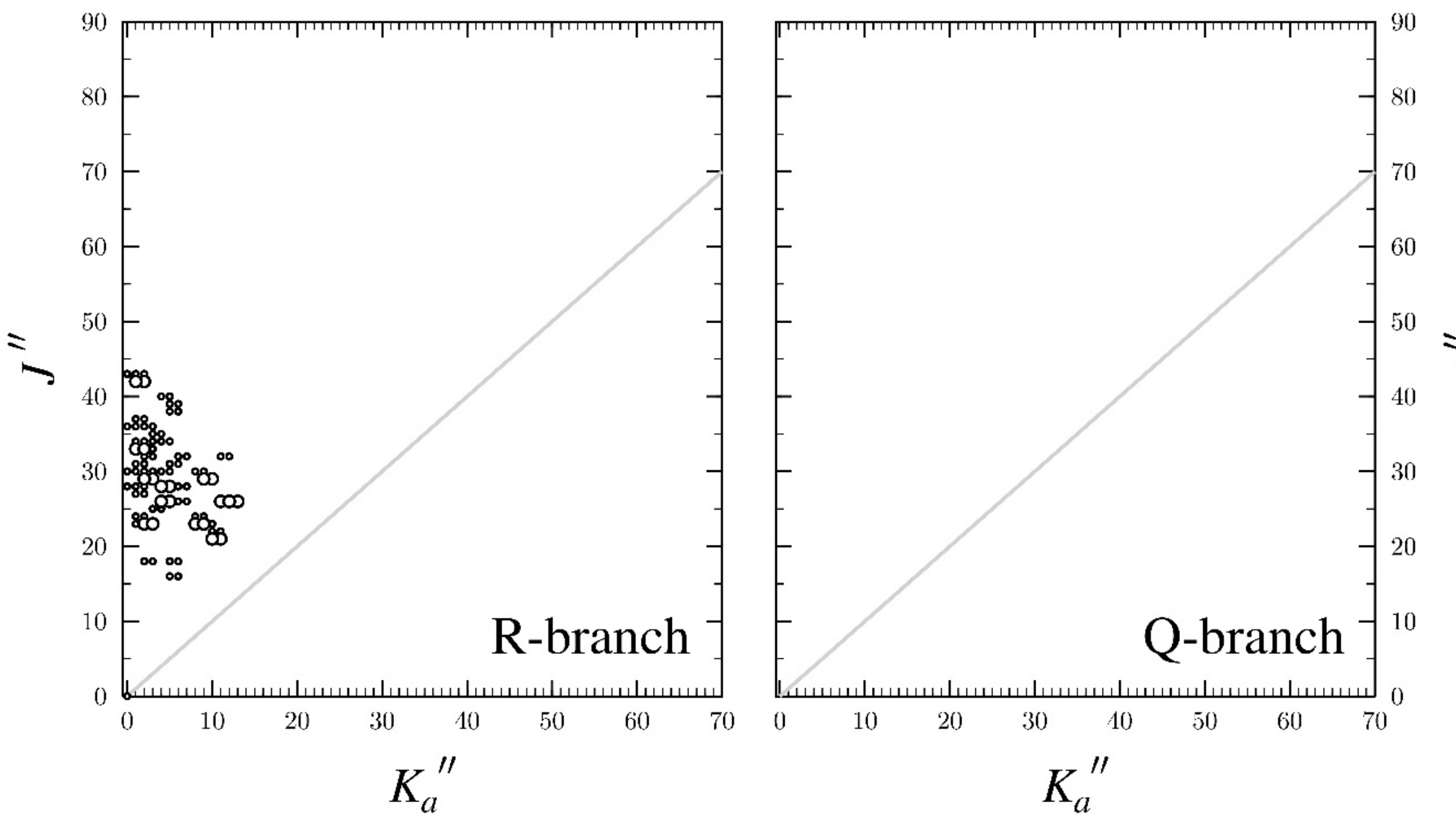

Fig. S9. Data distribution plot for the least-squares fit of spectroscopic data for the vibrational ground state, [2-^{2}H, 5-^{13}C]-furan isotopologue. The size of the outlined circle is proportional to the value of $|(f_{obs.} - f_{calc.})/\delta f|$, where δf is the frequency measurement uncertainty, and all values represented are less than or equal to 3.

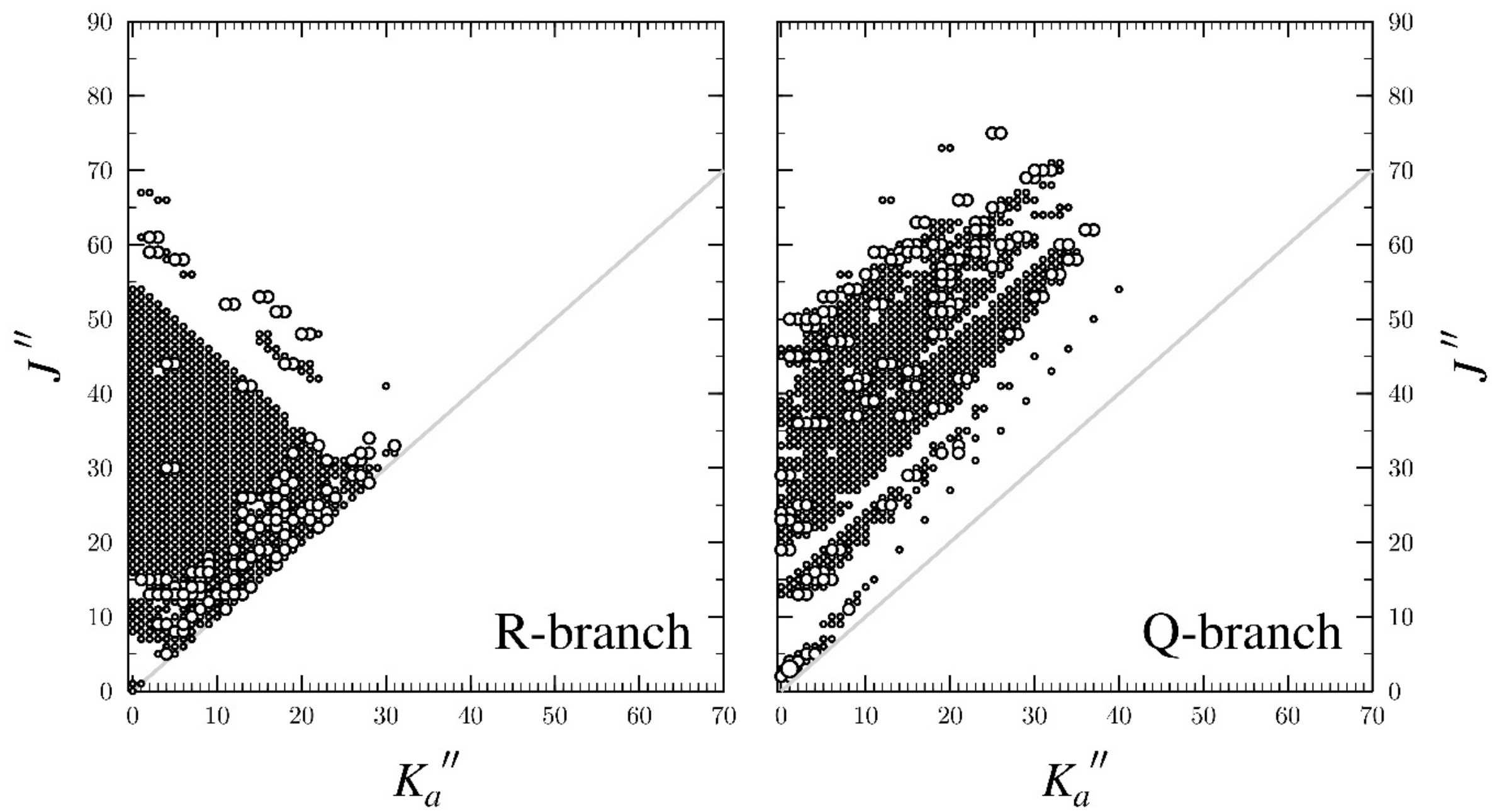

Fig. S10. Data distribution plot for the least-squares fit of spectroscopic data for the vibrational ground state, [3-^{2}H]-furan isotopologue. The size of the outlined circle is proportional to the value of $|(f_{obs.} - f_{calc.})/\delta f|$, where δf is the frequency measurement uncertainty, and all values represented are less than or equal to 3.

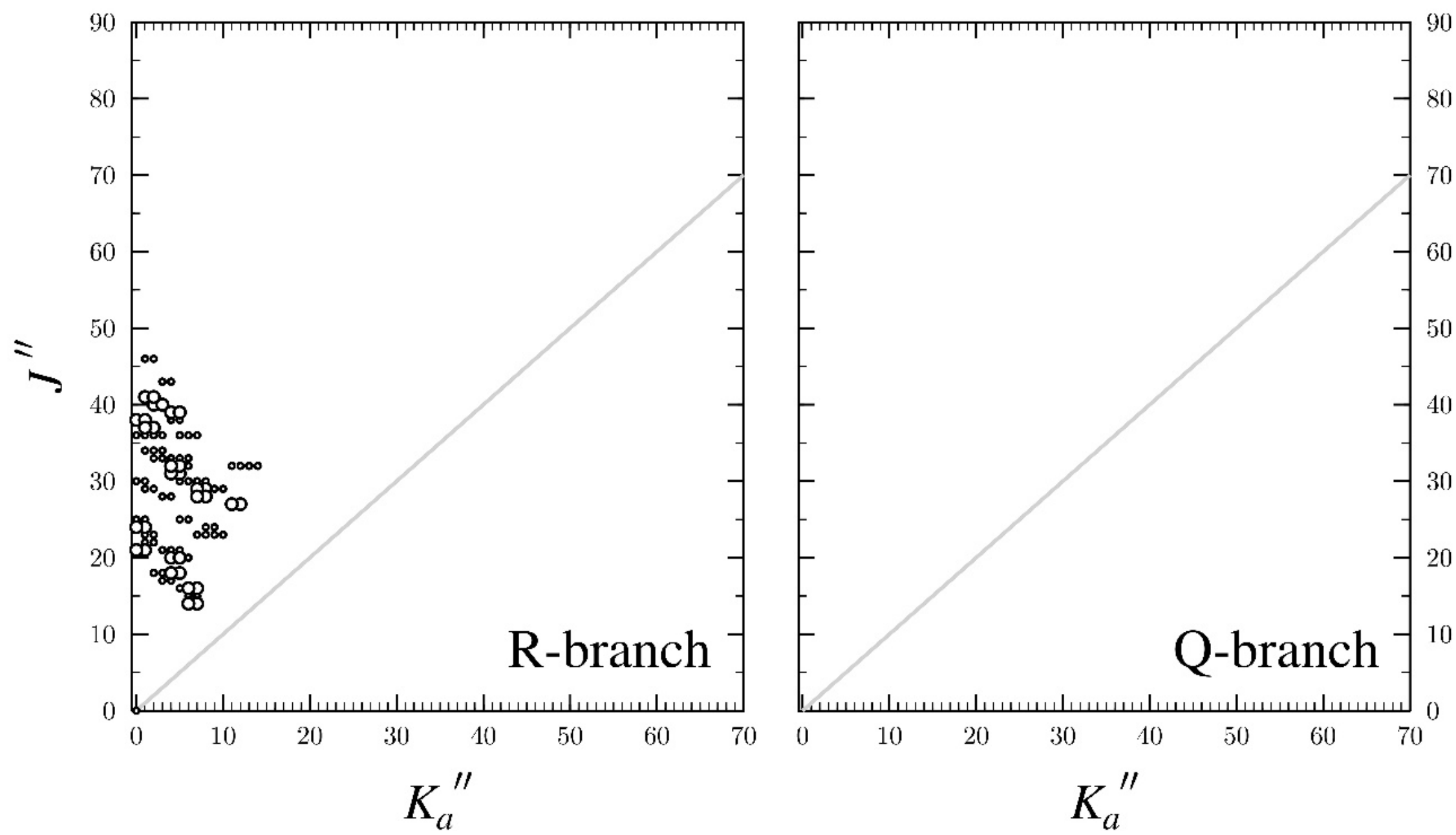

Fig. S11. Data distribution plot for the least-squares fit of spectroscopic data for the vibrational ground state, [3-^{2}H, 2-^{13}C]-furan isotopologue. The size of the outlined circle is proportional to the value of $|(f_{obs.} - f_{calc.})/\delta f|$, where δf is the frequency measurement uncertainty, and all values represented are less than or equal to 3.

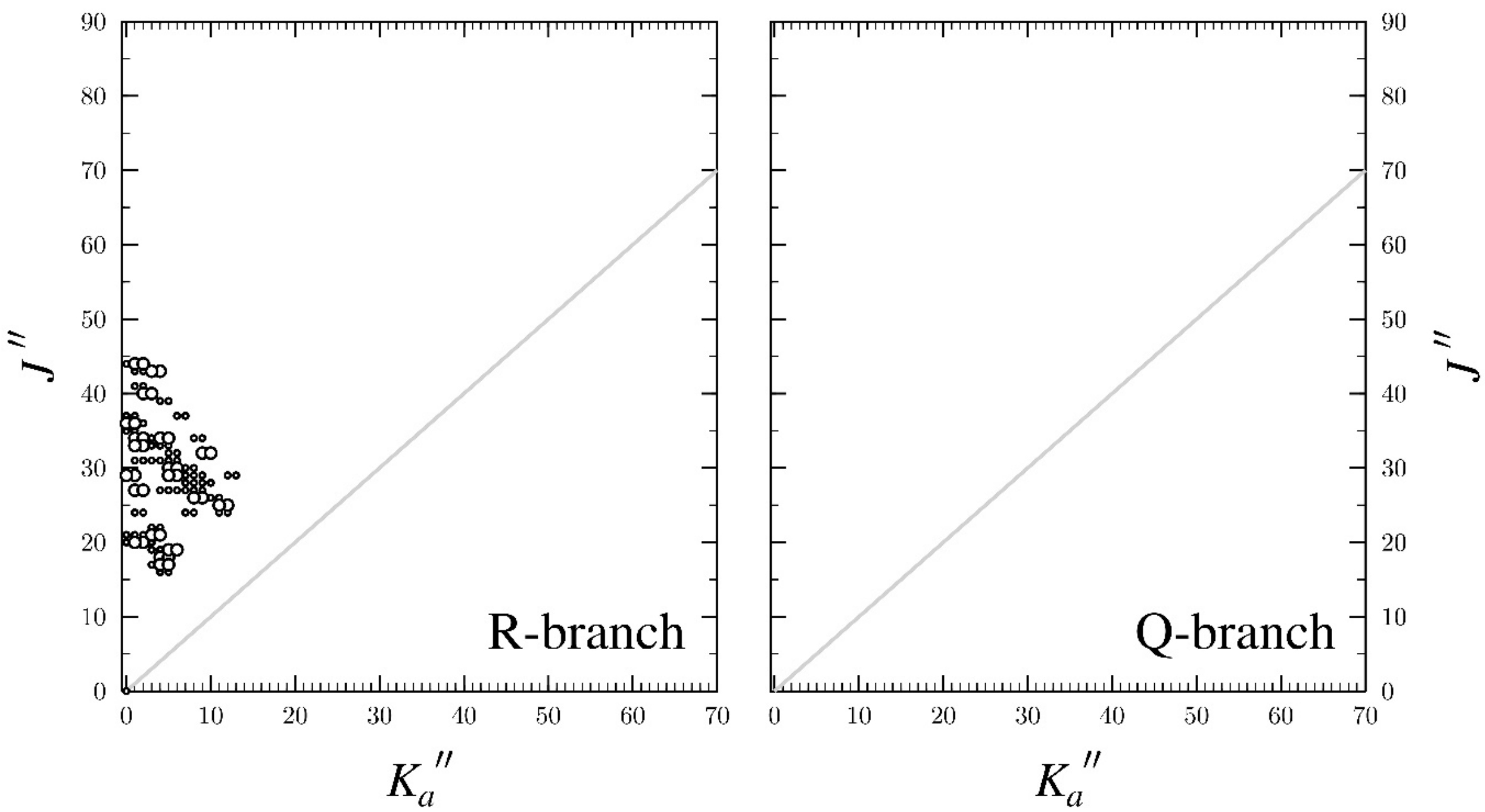

Fig. S12. Data distribution plot for the least-squares fit of spectroscopic data for the vibrational ground state, [3-^{2}H, 3-^{13}C]-furan isotopologue. The size of the outlined circle is proportional to the value of $|(f_{obs.} - f_{calc.})/\delta f|$, where δf is the frequency measurement uncertainty, and all values represented are less than or equal to 3.

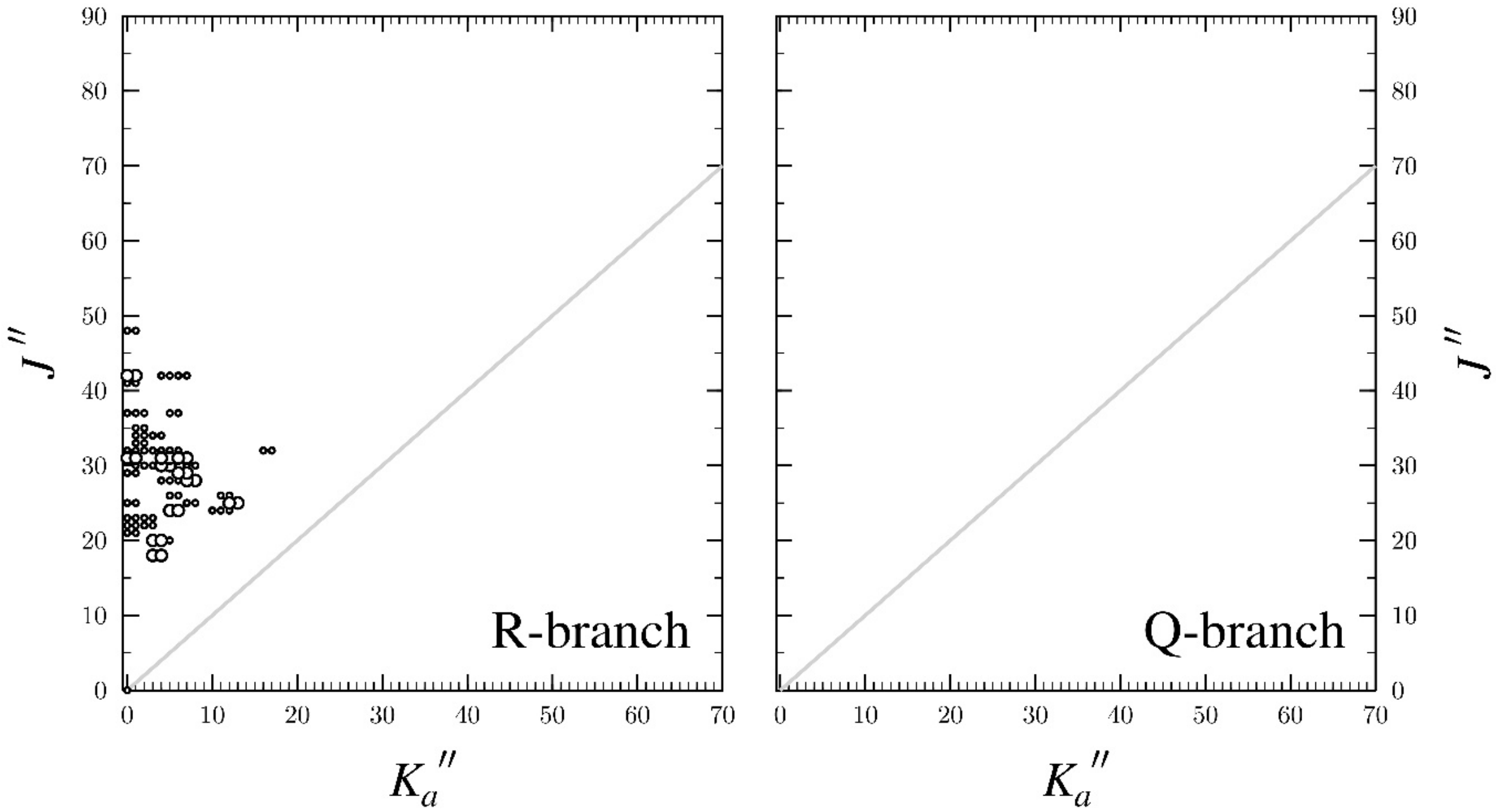

Fig. S13. Data distribution plot for the least-squares fit of spectroscopic data for the vibrational ground state, [3-^{2}H, 4-^{13}C]-furan isotopologue. The size of the outlined circle is proportional to the value of $|(f_{obs.} - f_{calc.})/\delta f|$, where δf is the frequency measurement uncertainty, and all values represented are less than or equal to 3.

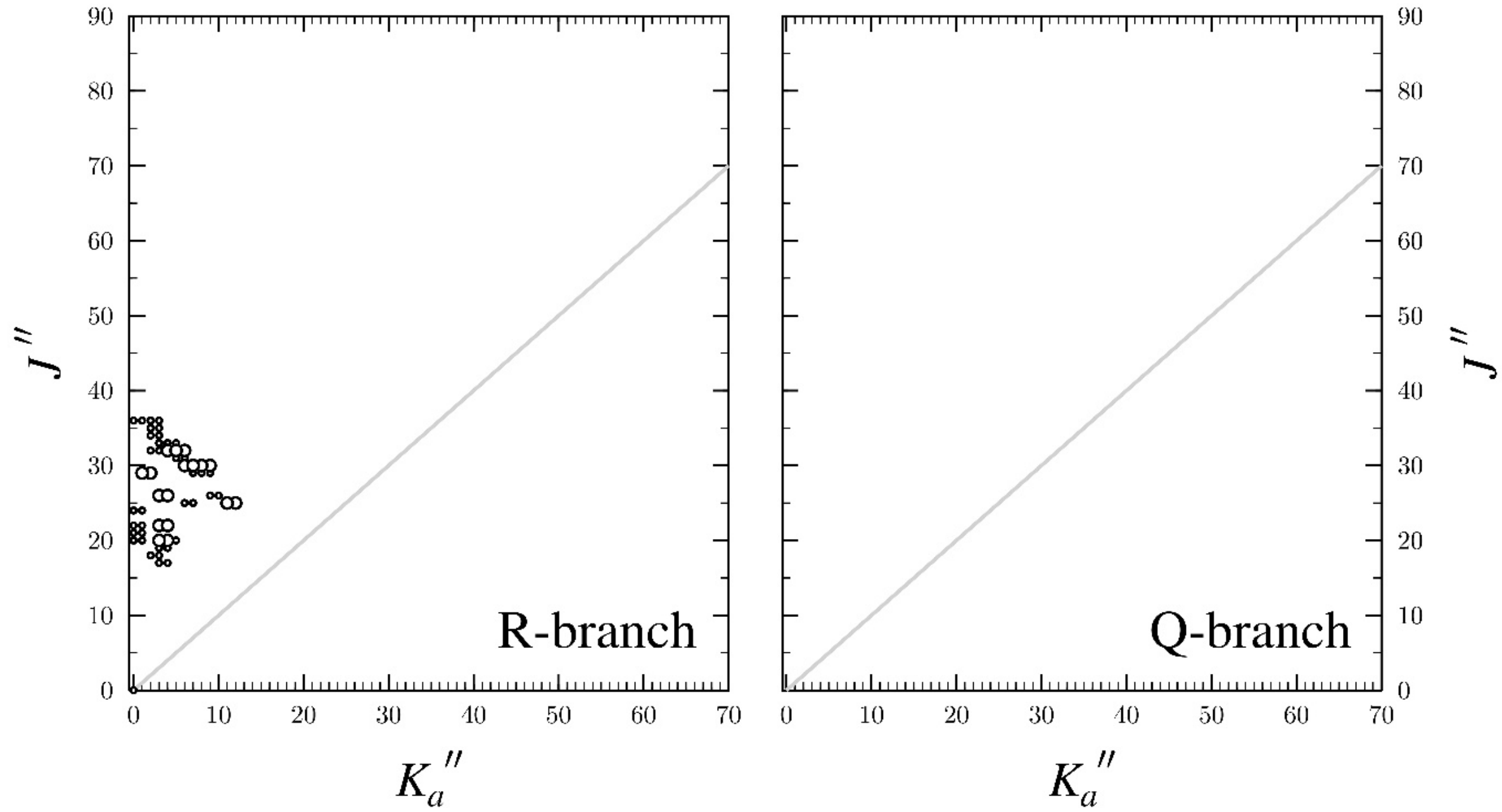

Fig. S14. Data distribution plot for the least-squares fit of spectroscopic data for the vibrational ground state, [3-^{2}H, 5-^{13}C]-furan isotopologue. The size of the outlined circle is proportional to the value of $|(f_{obs.} - f_{calc.})/\delta f|$, where δf is the frequency measurement uncertainty, and all values represented are less than or equal to 3.

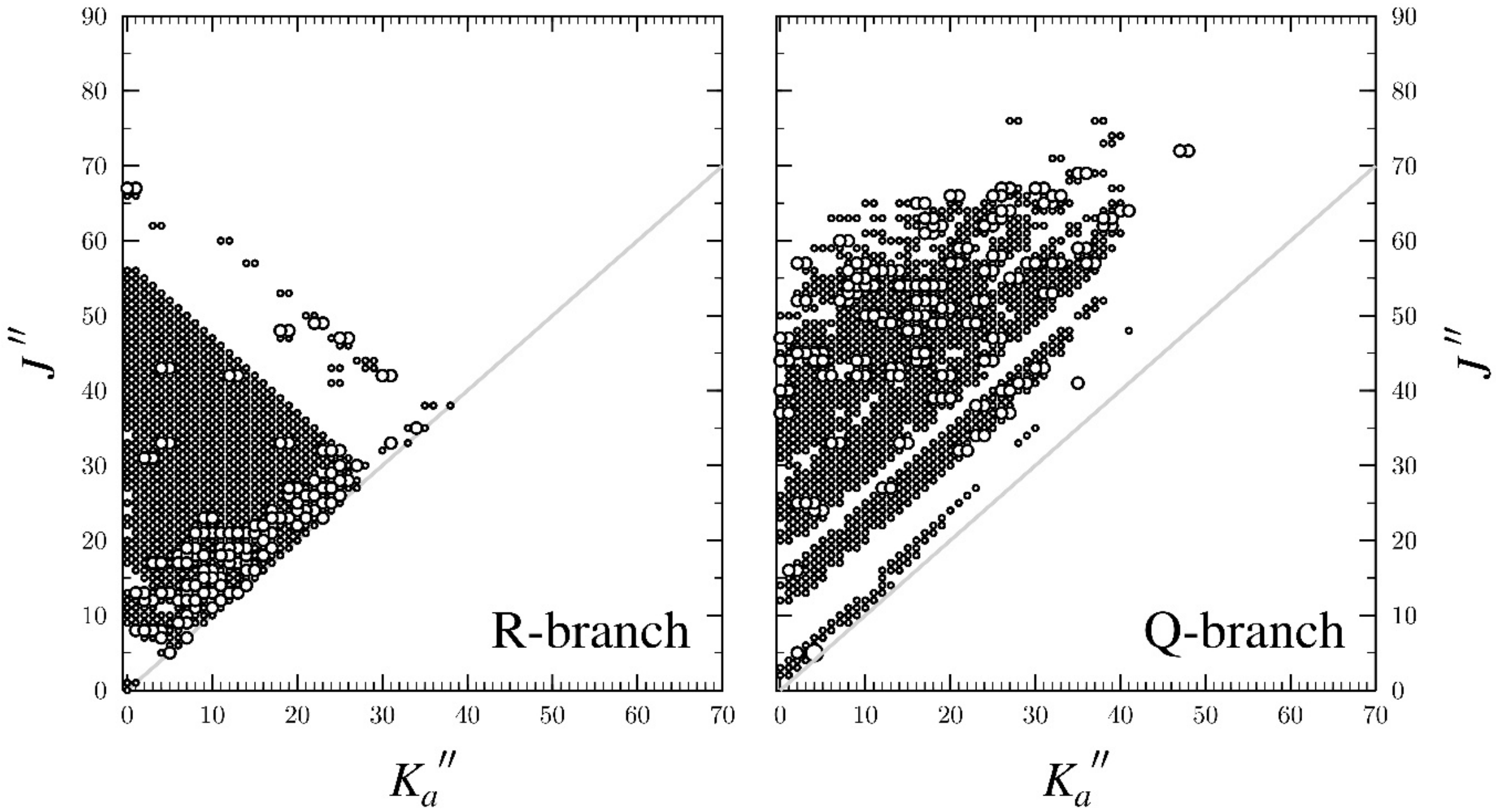

Fig. S15. Data distribution plot for the least-squares fit of spectroscopic data for the vibrational ground state, [2,3-^{2}H]-furan isotopologue. The size of the outlined circle is proportional to the value of $|(f_{obs.} - f_{calc.})/\delta f|$, where δf is the frequency measurement uncertainty, and all values represented are less than or equal to 3.

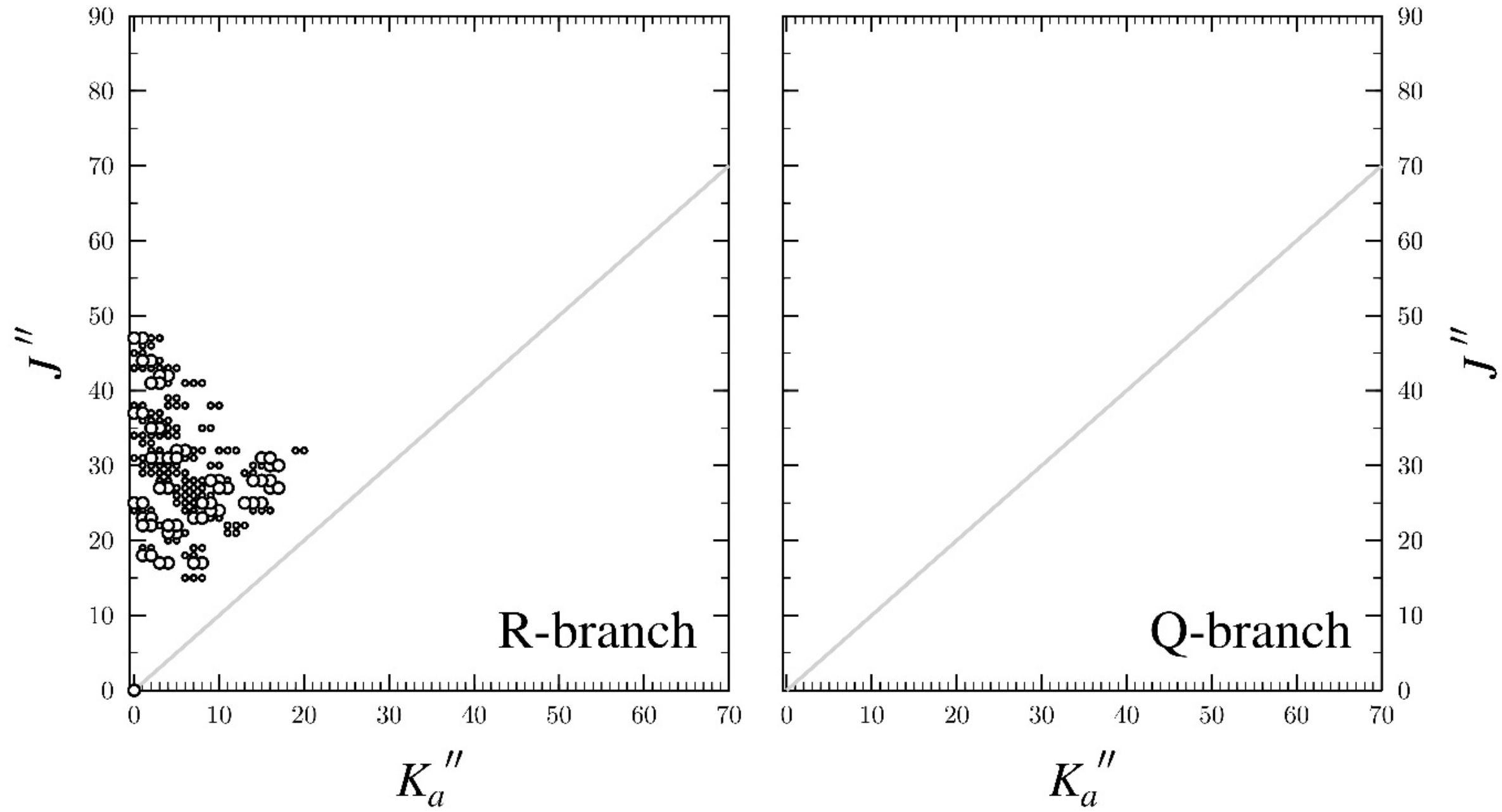

Fig. S16. Data distribution plot for the least-squares fit of spectroscopic data for the vibrational ground state, [2,3-^{2}H, 2-^{13}C]-furan isotopologue. The size of the outlined circle is proportional to the value of $|(f_{obs.} - f_{calc.})/\delta f|$, where δf is the frequency measurement uncertainty, and all values represented are less than or equal to 3.

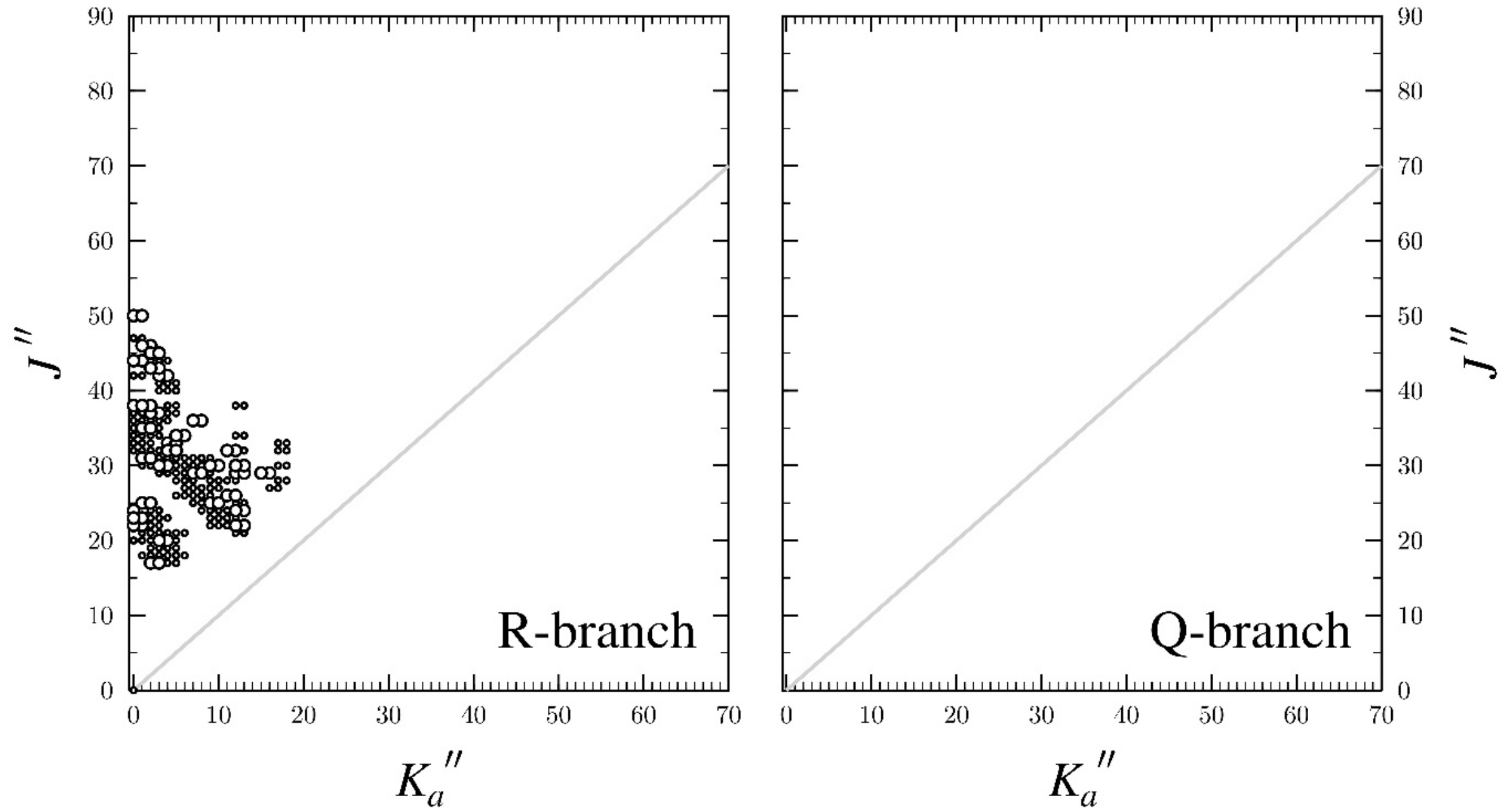

Fig. S17. Data distribution plot for the least-squares fit of spectroscopic data for the vibrational ground state, [2,3-^{2}H, 3-^{13}C]-furan isotopologue. The size of the outlined circle is proportional to the value of $|(f_{obs.} - f_{calc.})/\delta f|$, where δf is the frequency measurement uncertainty, and all values represented are less than or equal to 3.

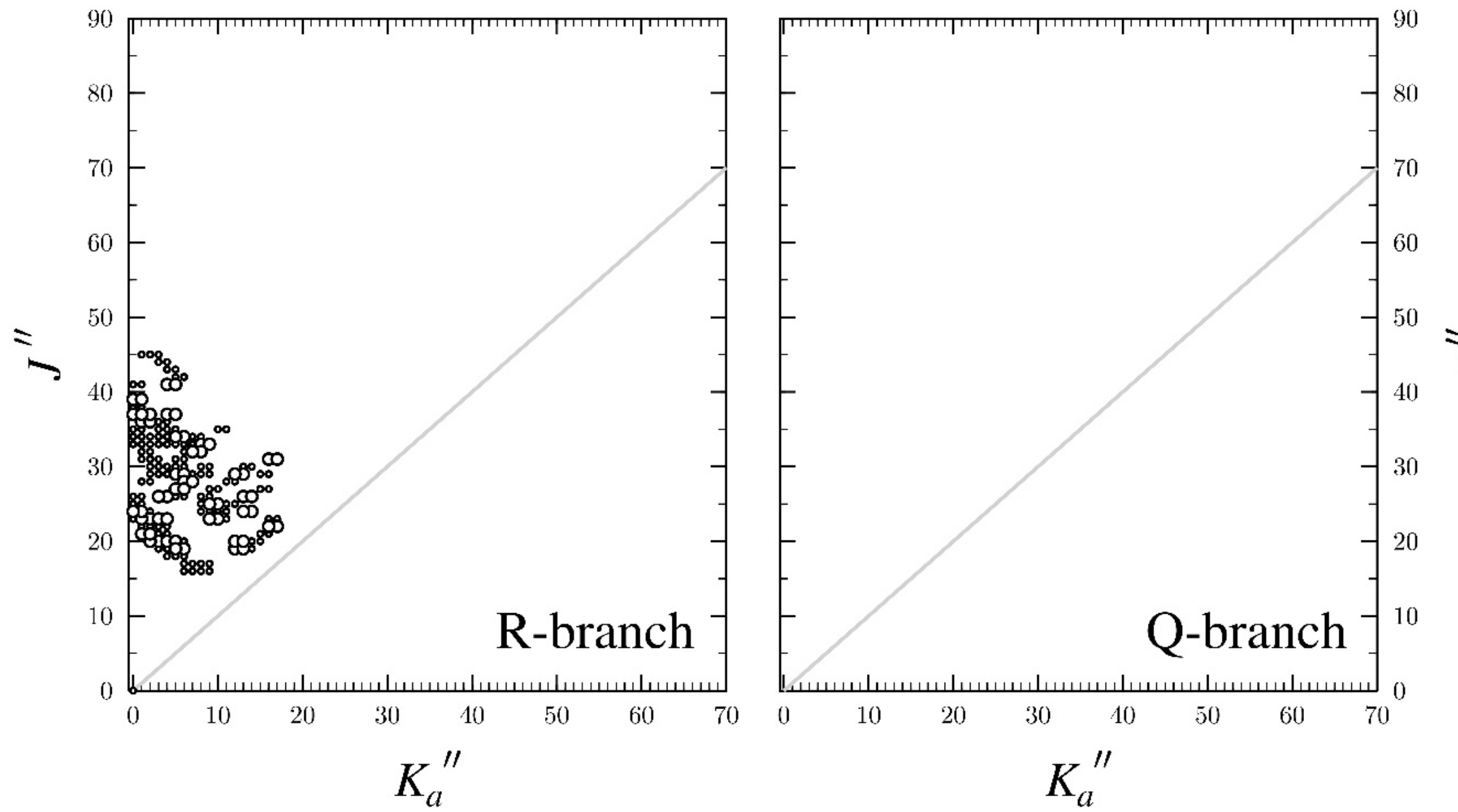

Fig. S18. Data distribution plot for the least-squares fit of spectroscopic data for the vibrational ground state, [2,3-^{2}H, 4-^{13}C]-furan isotopologue. The size of the outlined circle is proportional to the value of $|(f_{obs.} - f_{calc.})/\delta f|$, where δf is the frequency measurement uncertainty, and all values represented are less than or equal to 3.

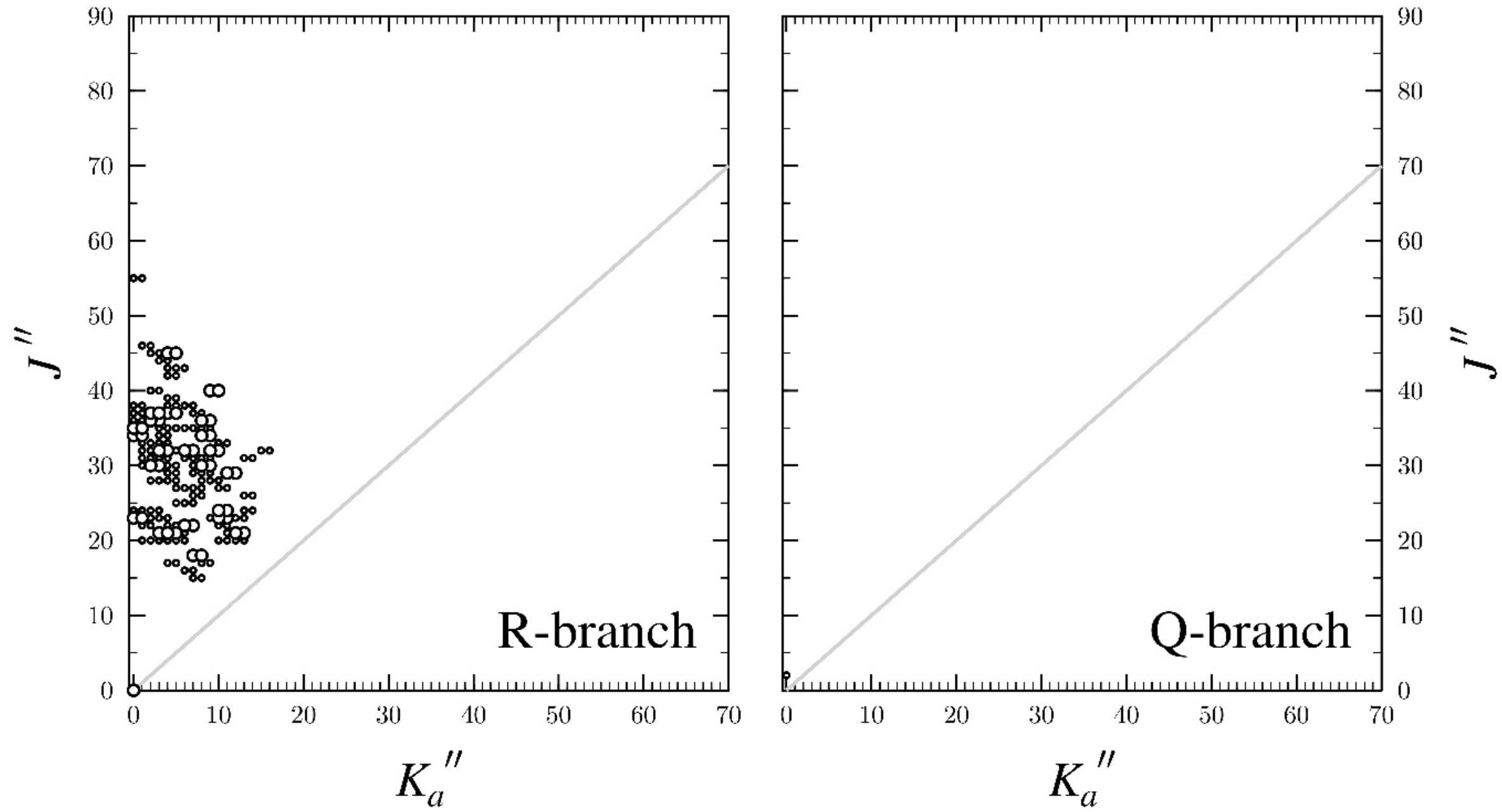

Fig. S19. Data distribution plot for the least-squares fit of spectroscopic data for the vibrational ground state, [2,3-^{2}H, 5-^{13}C]-furan isotopologue. The size of the outlined circle is proportional to the value of $|(f_{obs.} - f_{calc.})/\delta f|$, where δf is the frequency measurement uncertainty, and all values represented are less than or equal to 3.

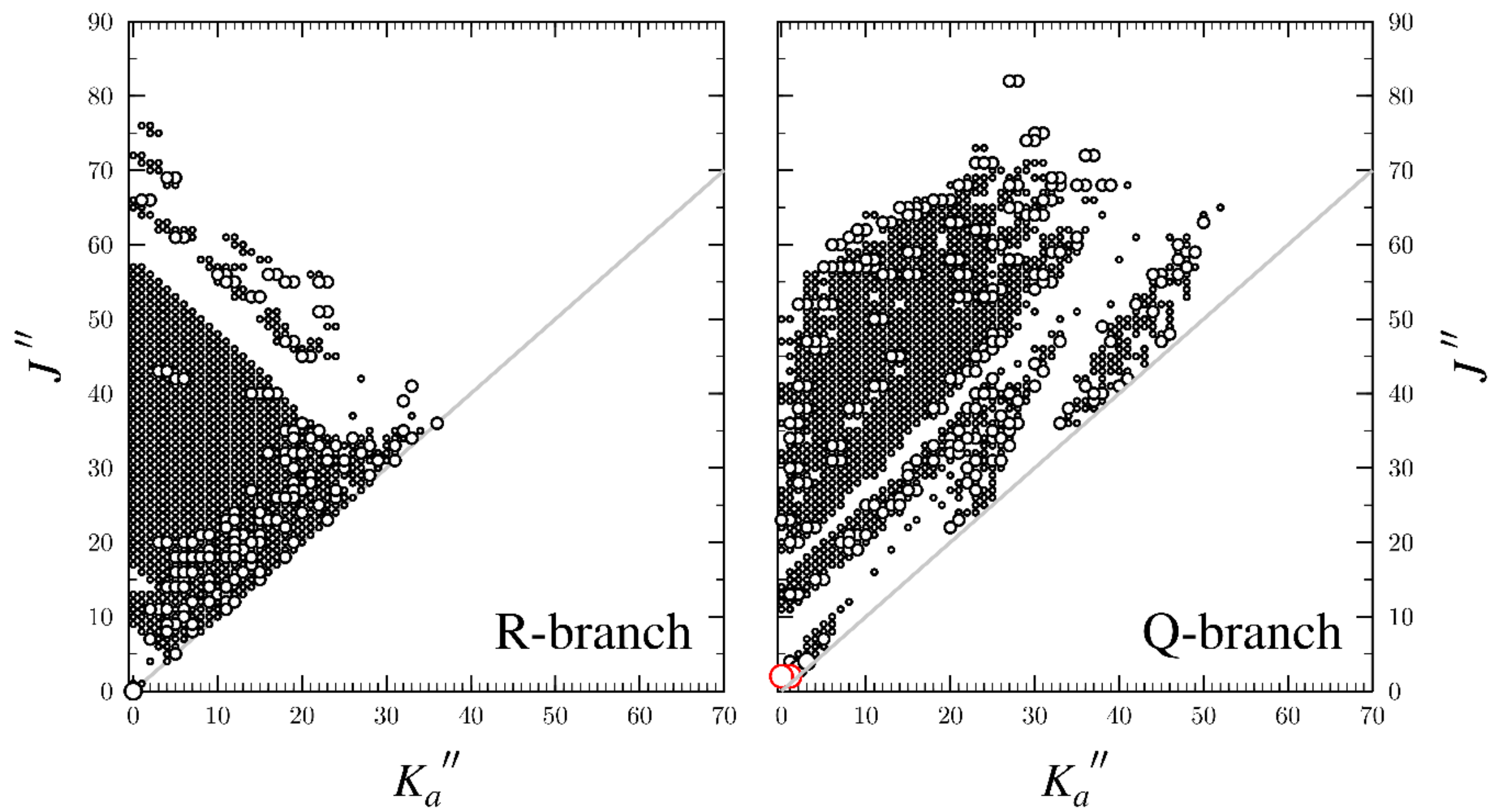

Fig. S20. Data distribution plot for the least-squares fit of spectroscopic data for the vibrational ground state, [2,4-^{2}H]-furan isotopologue. The size of the outlined circle is proportional to the value of $|(f_{obs.} - f_{calc.})/\delta f|$, where δf is the frequency measurement uncertainty, and all values represented are less than or equal to 4. Data points with measurement uncertainties larger than 3 are shown in red.

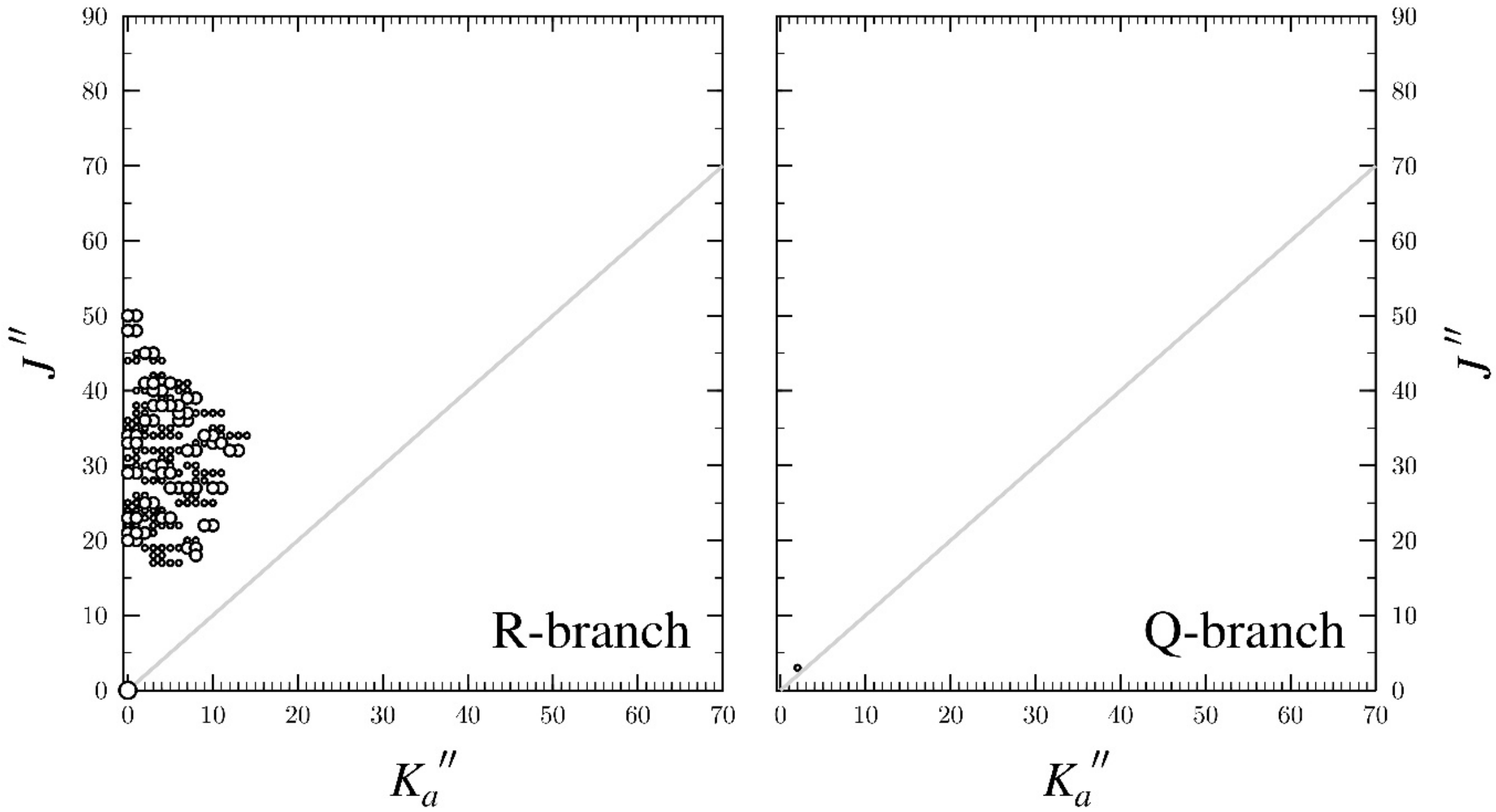

Fig. S21. Data distribution plot for the least-squares fit of spectroscopic data for the vibrational ground state, [2,4-^{2}H, 2-^{13}C]-furan isotopologue. The size of the outlined circle is proportional to the value of $|(f_{obs.} - f_{calc.})/\delta f|$, where δf is the frequency measurement uncertainty, and all values represented are less than or equal to 3.

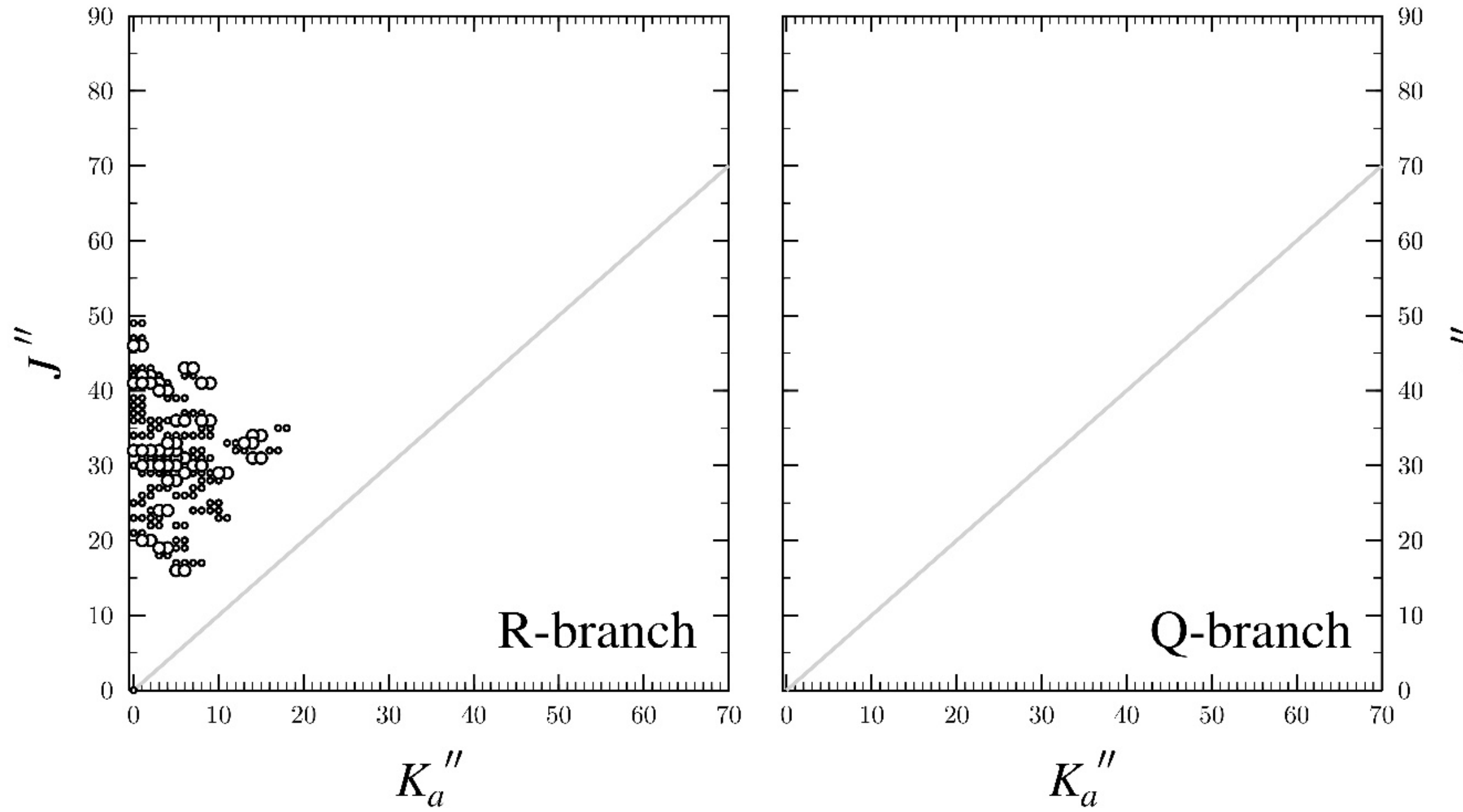

Fig. S22. Data distribution plot for the least-squares fit of spectroscopic data for the vibrational ground state, [2,4-^{2}H, 3-^{13}C]-furan isotopologue. The size of the outlined circle is proportional to the value of $|(f_{obs.} - f_{calc.})/\delta f|$, where δf is the frequency measurement uncertainty, and all values represented are less than or equal to 3.

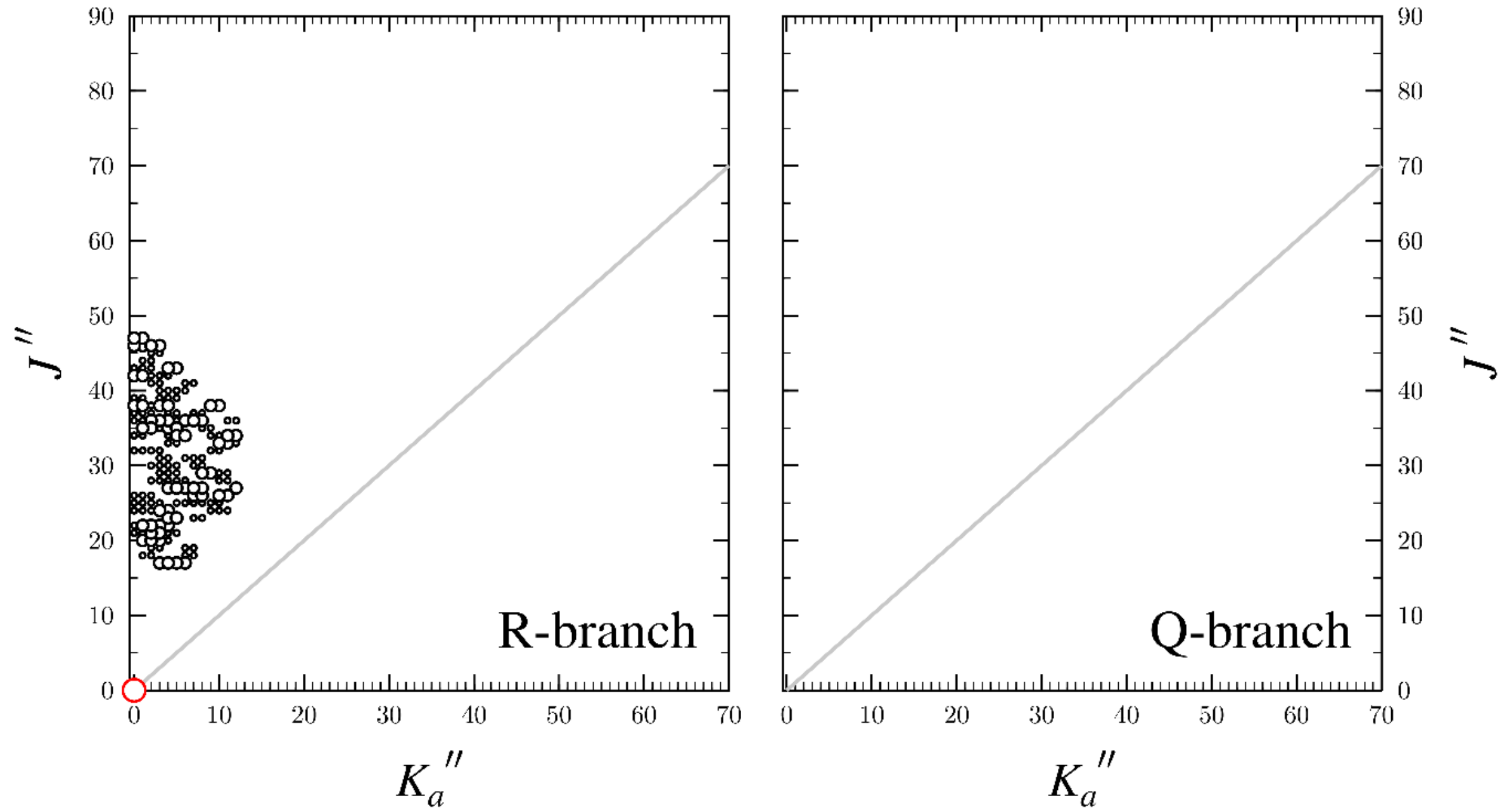

Fig. S23. Data distribution plot for the least-squares fit of spectroscopic data for the vibrational ground state, [2,4-^{2}H, 4-^{13}C]-furan isotopologue. The size of the outlined circle is proportional to the value of $|(f_{obs.} - f_{calc.})/\delta f|$, where δf is the frequency measurement uncertainty, and all values represented are less than or equal to 4. Data points with measurement uncertainties larger than 3 are shown in red.

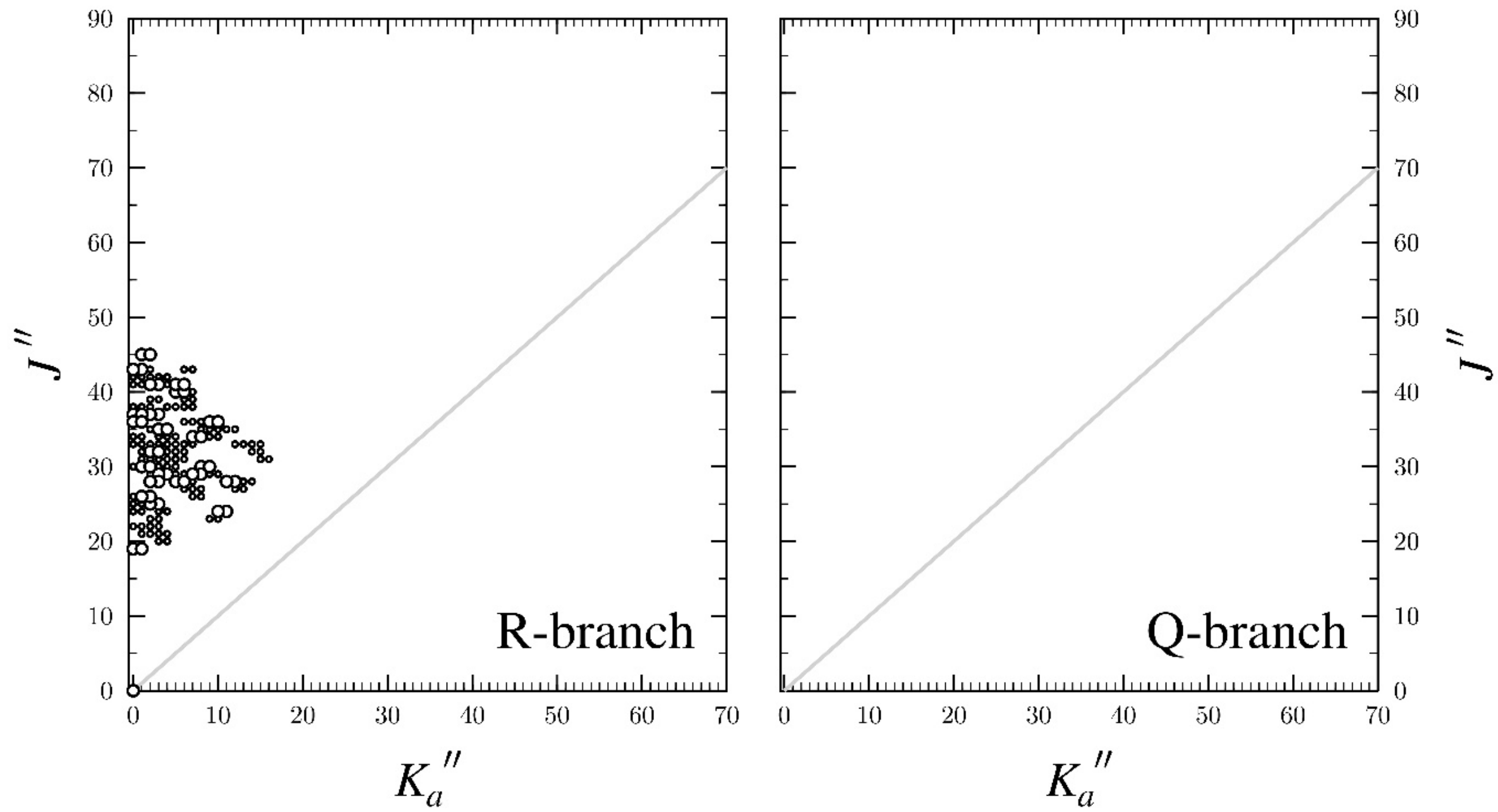

Fig. S24. Data distribution plot for the least-squares fit of spectroscopic data for the vibrational ground state, [2,4-^{2}H, 5-^{13}C]-furan isotopologue. The size of the outlined circle is proportional to the value of $|(f_{obs.} - f_{calc.})/\delta f|$, where δf is the frequency measurement uncertainty, and all values represented are less than or equal to 3.

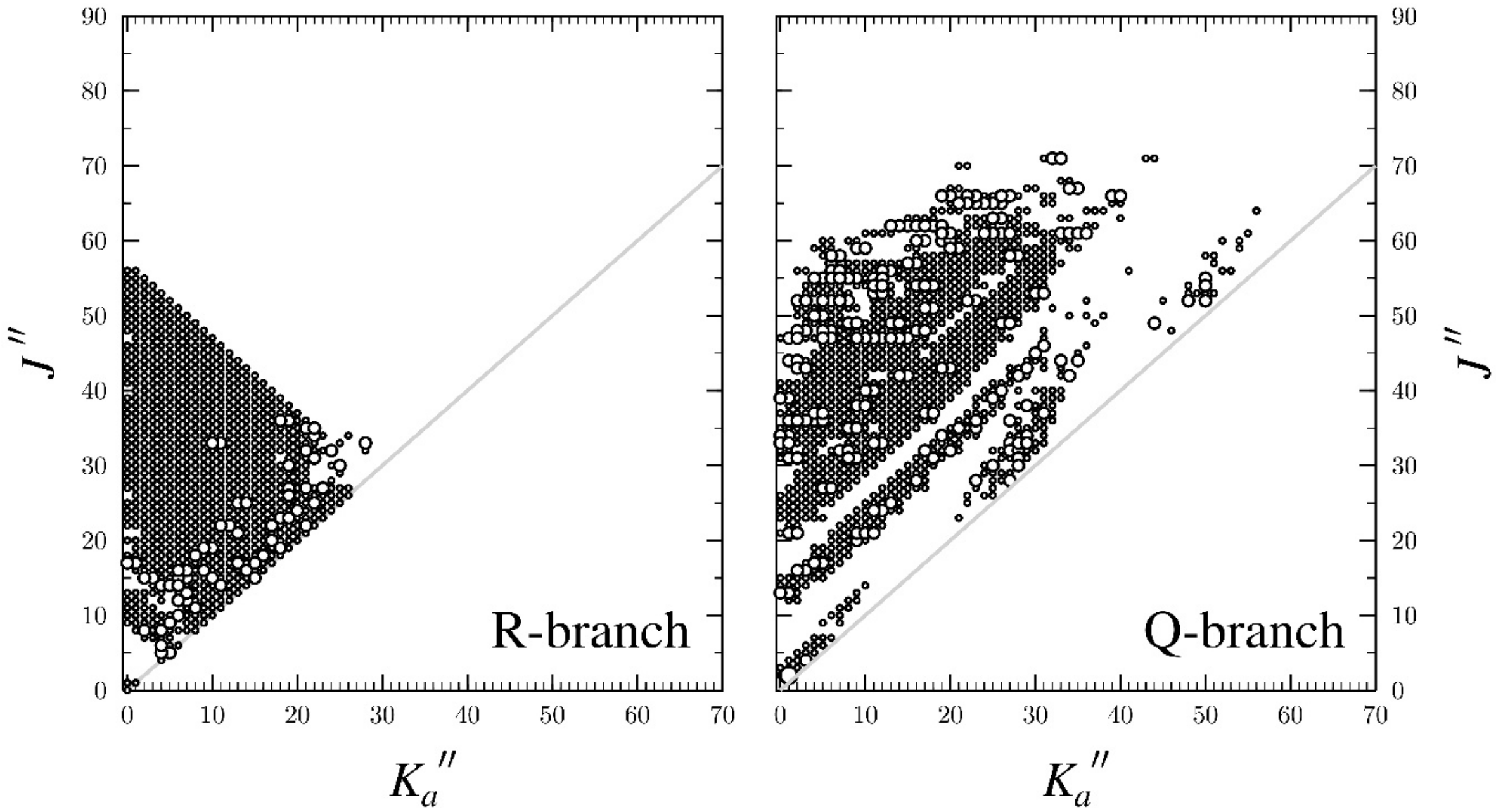

Fig. S25. Data distribution plot for the least-squares fit of spectroscopic data for the vibrational ground state, [2,5-^{2}H]-furan isotopologue. The size of the outlined circle is proportional to the value of $|(f_{obs.} - f_{calc.})/\delta f|$, where δf is the frequency measurement uncertainty, and all values represented are less than or equal to 3.

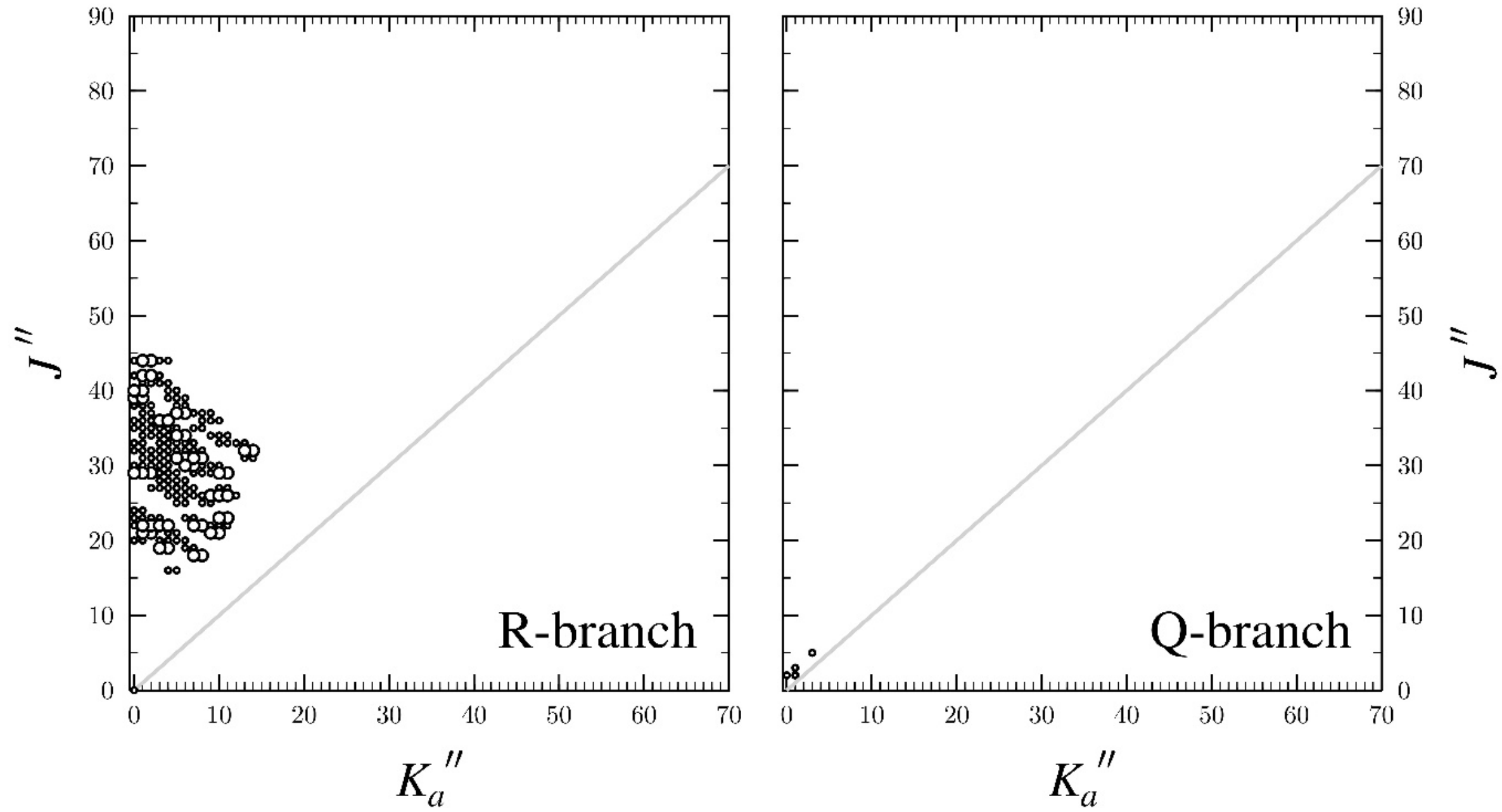

Fig. S26. Data distribution plot for the least-squares fit of spectroscopic data for the vibrational ground state, [2,5-^{2}H, 2-^{13}C]-furan isotopologue. The size of the outlined circle is proportional to the value of $|(f_{obs.} - f_{calc.})/\delta f|$, where δf is the frequency measurement uncertainty, and all values represented are less than or equal to 3.

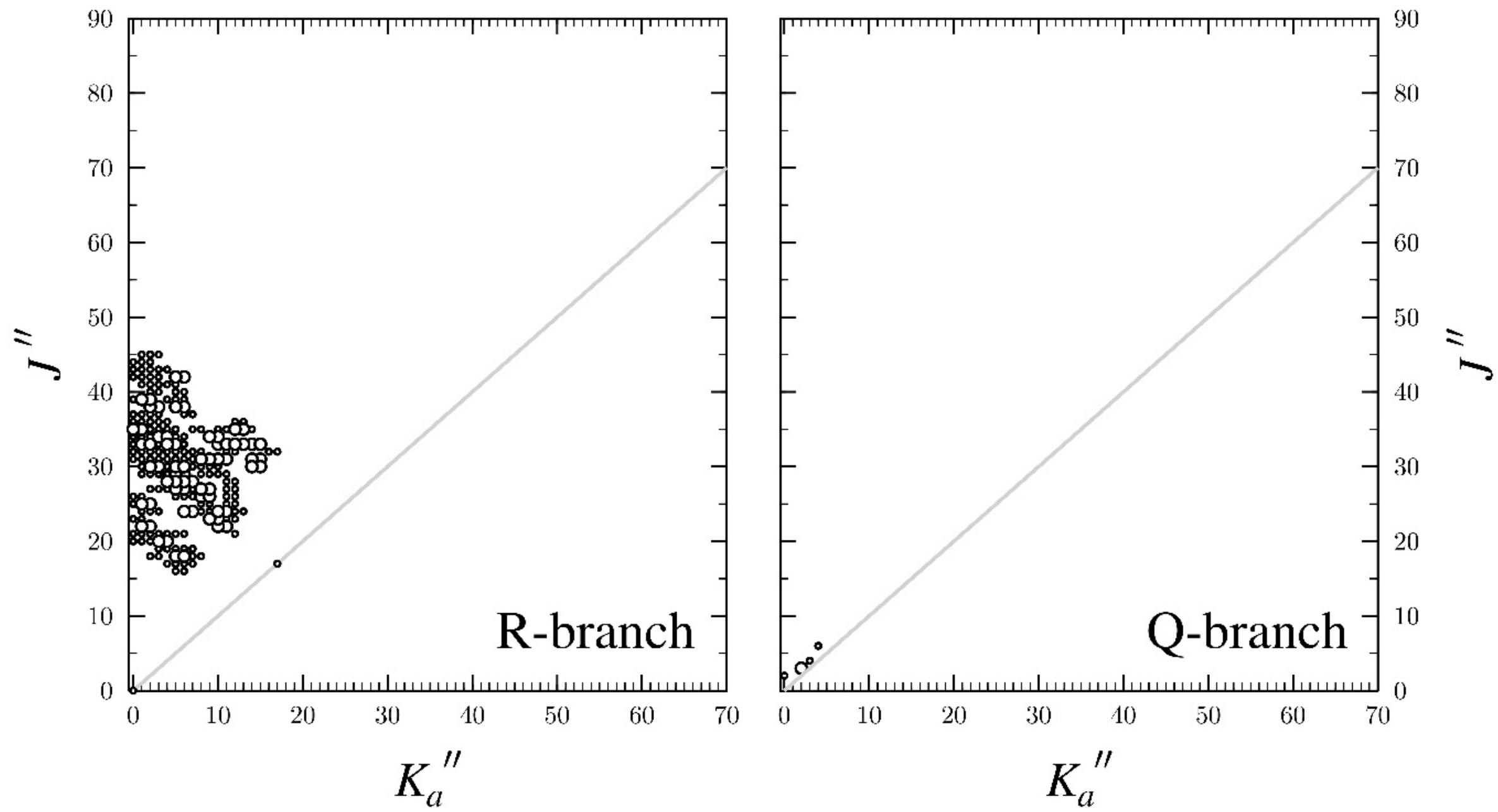

Fig. S27. Data distribution plot for the least-squares fit of spectroscopic data for the vibrational ground state, [2,5-^{2}H, 3-^{13}C]-furan isotopologue. The size of the outlined circle is proportional to the value of $|(f_{obs.} - f_{calc.})/\delta f|$, where δf is the frequency measurement uncertainty, and all values represented are less than or equal to 3.

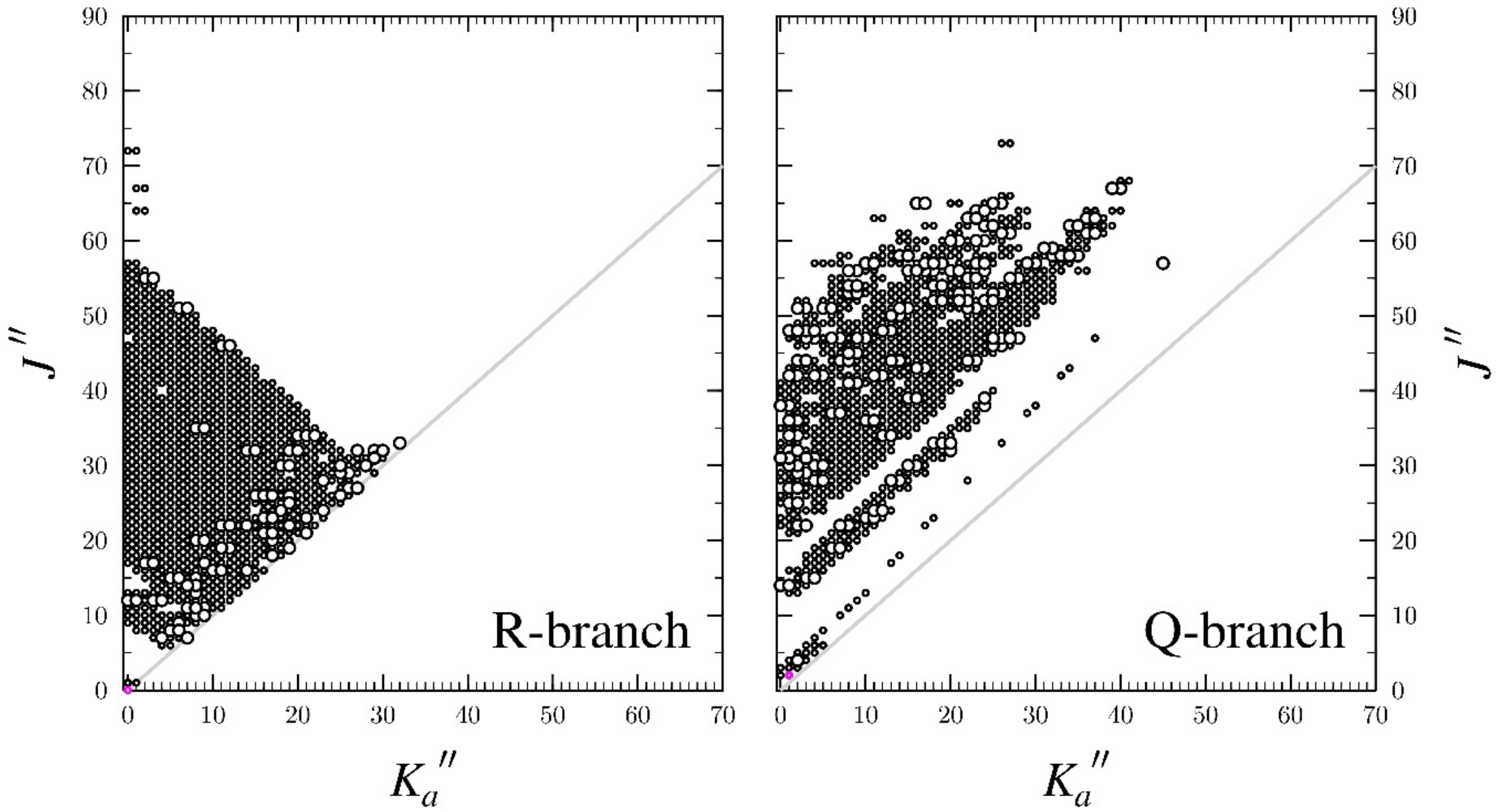

Fig. S28. Data distribution plot for the least-squares fit of spectroscopic data for the vibrational ground state, [3,4-^{2}H]-furan isotopologue. Black circles are measurements from the current work, while pink circles are from prior works.[22] The size of the outlined circle is proportional to the value of $|(f_{obs.} - f_{calc.})/\delta f|$, where δf is the frequency measurement uncertainty, and all values represented are less than or equal to 3.

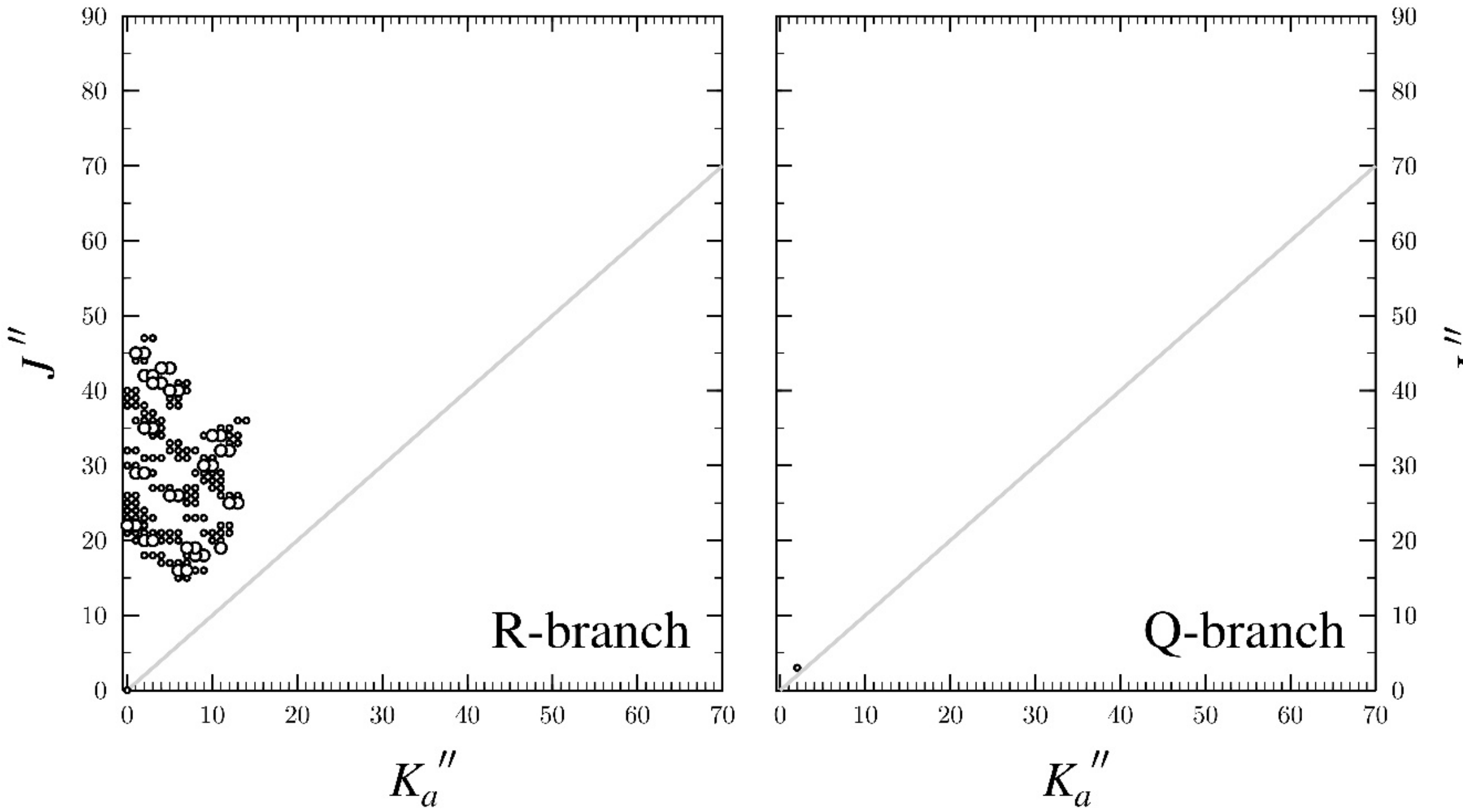

Fig. S29. Data distribution plot for the least-squares fit of spectroscopic data for the vibrational ground state, [3,4-^{2}H, 2-^{13}C]-furan isotopologue. The size of the outlined circle is proportional to the value of $|(f_{obs.} - f_{calc.})/\delta f|$, where δf is the frequency measurement uncertainty, and all values represented are less than or equal to 3.

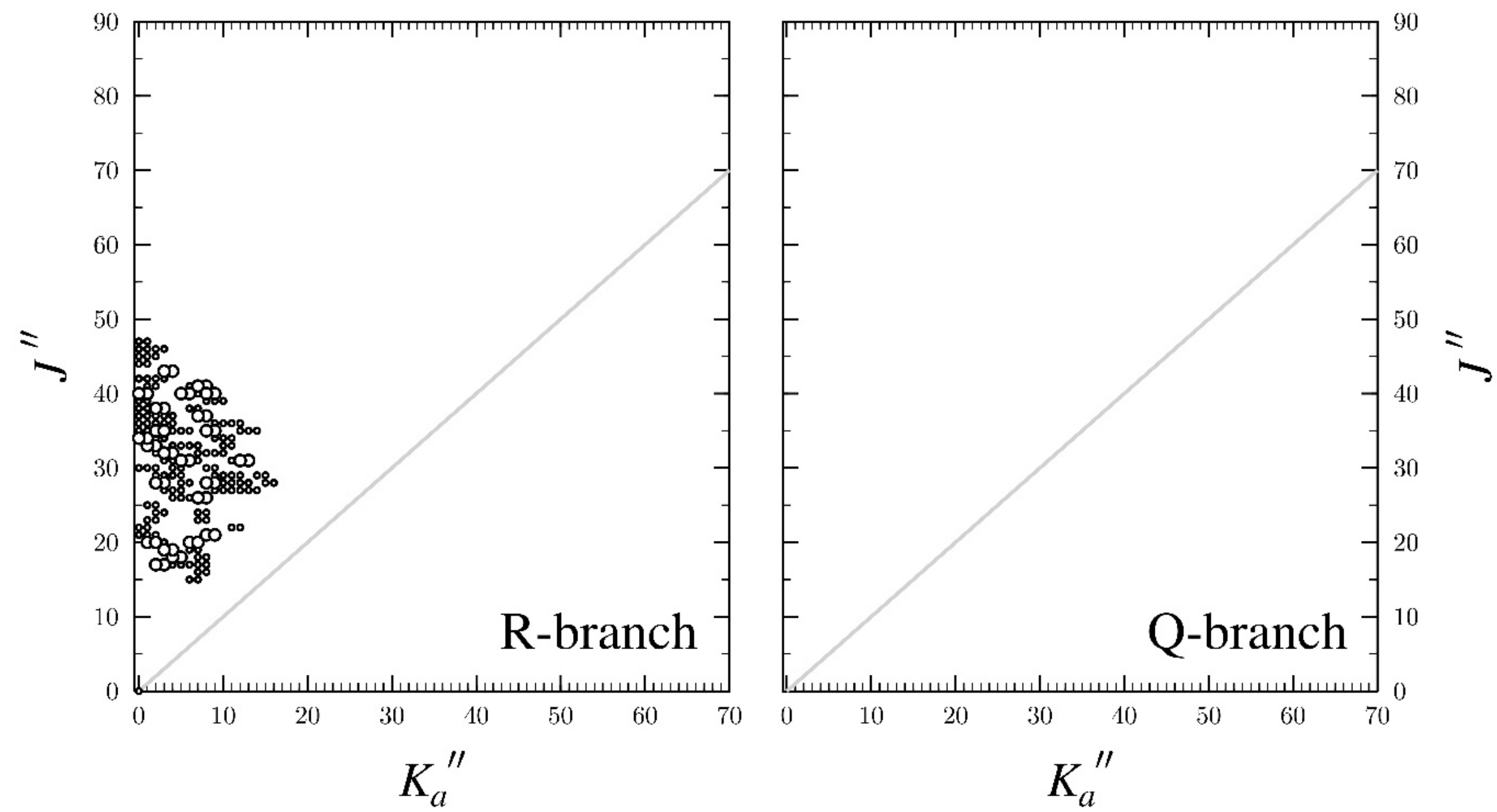

Fig. S30. Data distribution plot for the least-squares fit of spectroscopic data for the vibrational ground state, [3,4-^{2}H, 3-^{13}C]-furan isotopologue. The size of the outlined circle is proportional to the value of $|(f_{obs.} - f_{calc.})/\delta f|$, where δf is the frequency measurement uncertainty, and all values represented are less than or equal to 3.

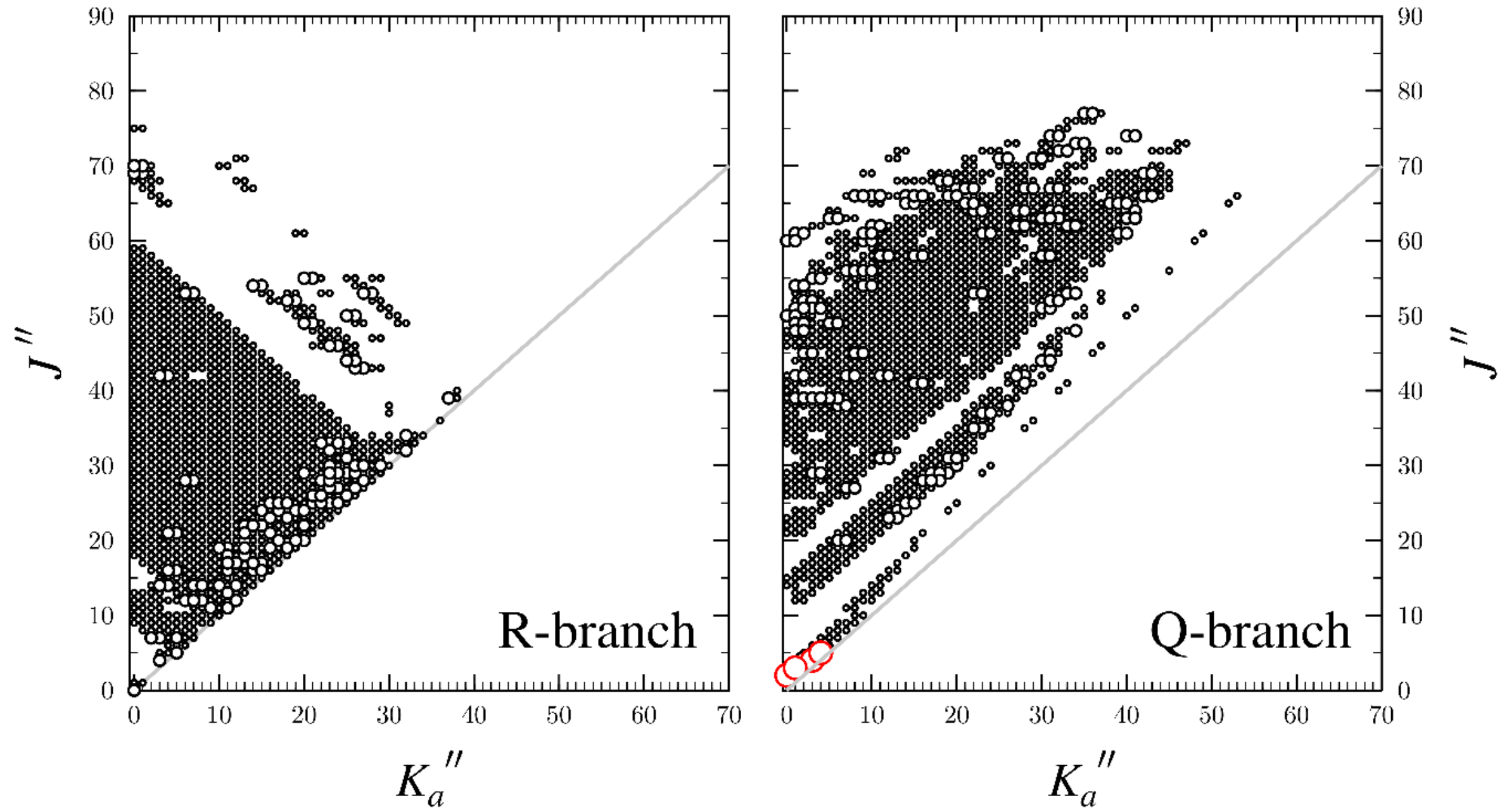

Fig. S31. Data distribution plot for the least-squares fit of spectroscopic data for the vibrational ground state, [2,3,4-^{2}H]-furan isotopologue. The size of the outlined circle is proportional to the value of $|(f_{obs.} - f_{calc.})/\delta f|$, where δf is the frequency measurement uncertainty, and all values represented are less than or equal to 4. Data points with measurement uncertainties larger than 3 are shown in red.

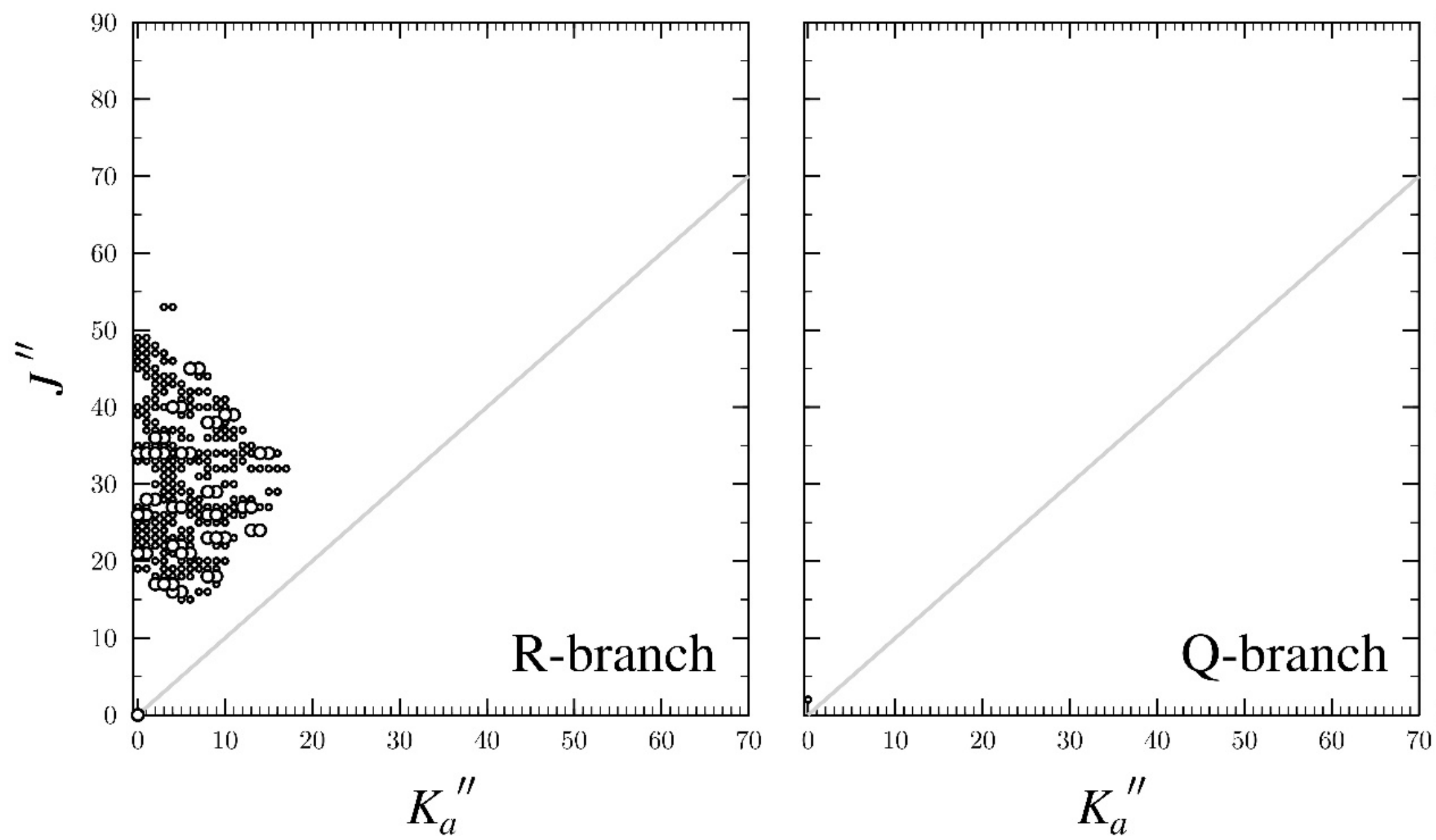

Fig. S32. Data distribution plot for the least-squares fit of spectroscopic data for the vibrational ground state, [2,3,4-^{2}H, 2-^{13}C]-furan isotopologue. The size of the outlined circle is proportional to the value of $|(f_{obs.} - f_{calc.})/\delta f|$, where δf is the frequency measurement uncertainty, and all values represented are less than or equal to 3.

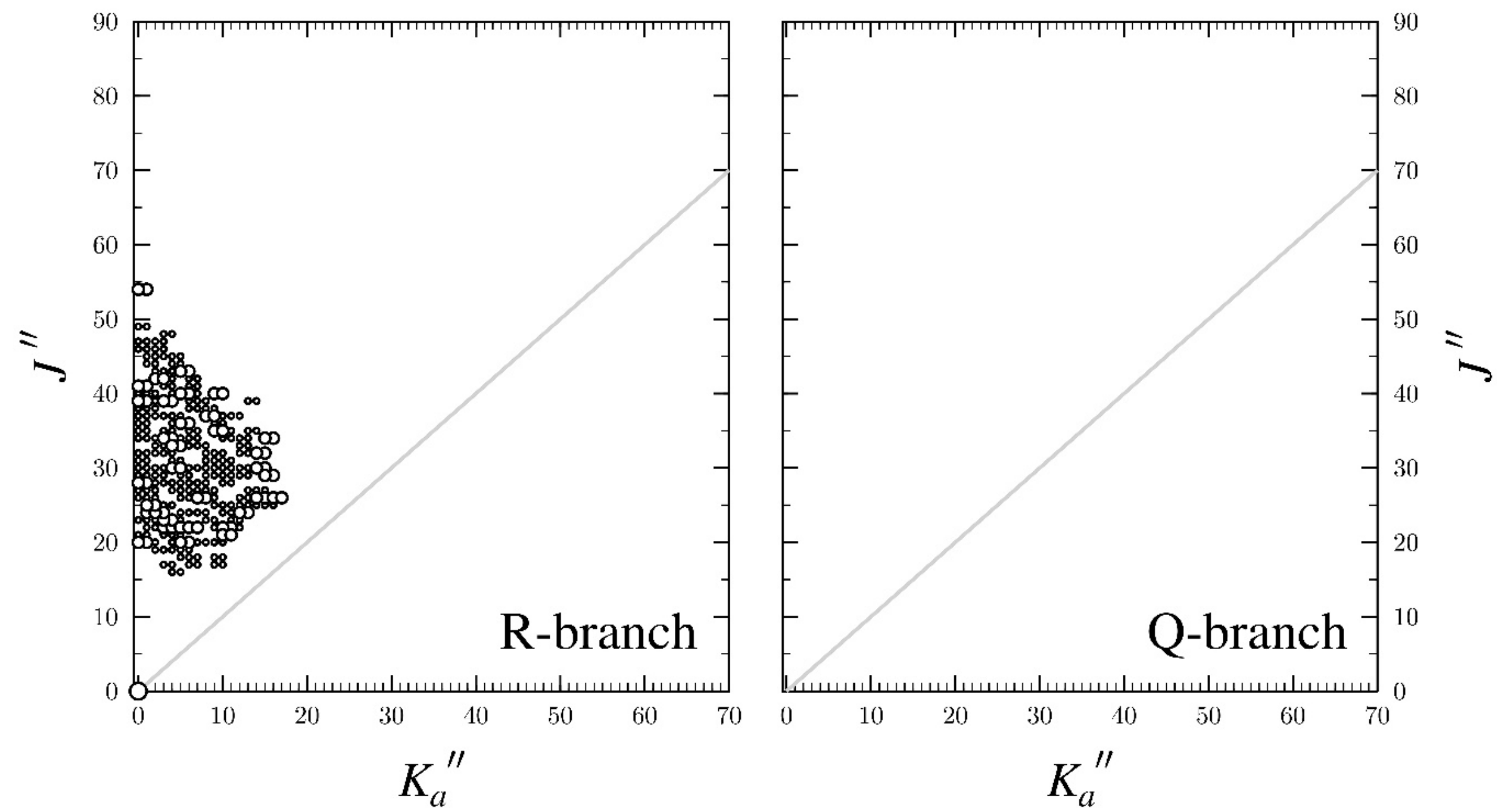

Fig. S33. Data distribution plot for the least-squares fit of spectroscopic data for the vibrational ground state, [2,3,4-^{2}H, 3-^{13}C]-furan isotopologue. The size of the outlined circle is proportional to the value of $|(f_{obs.} - f_{calc.})/\delta f|$, where δf is the frequency measurement uncertainty, and all values represented are less than or equal to 3.

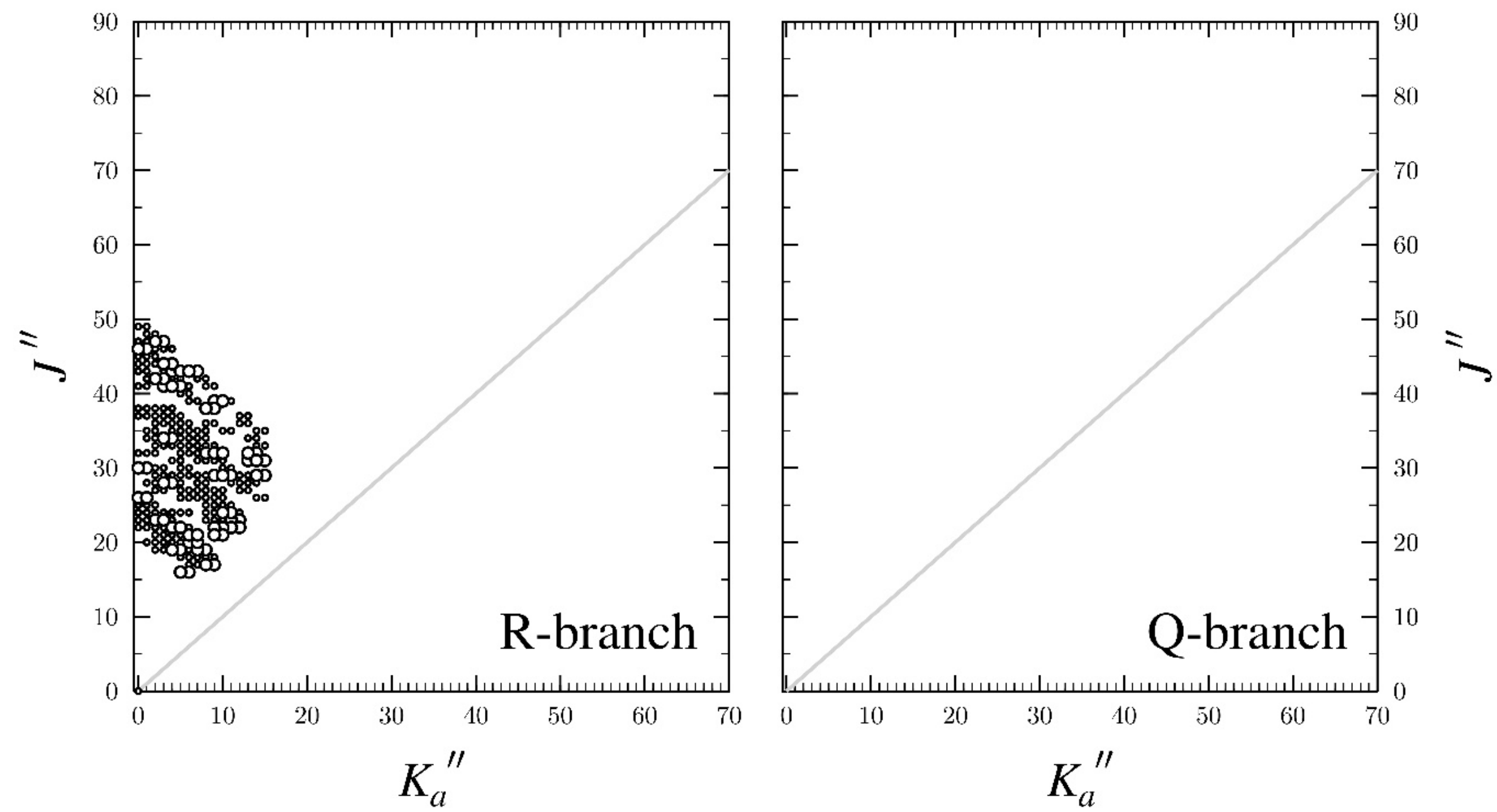

Fig. S34. Data distribution plot for the least-squares fit of spectroscopic data for the vibrational ground state, [2,3,4-^{2}H, 4-^{13}C]-furan isotopologue. The size of the outlined circle is proportional to the value of $|(f_{obs.} - f_{calc.})/\delta f|$, where δf is the frequency measurement uncertainty, and all values represented are less than or equal to 3.

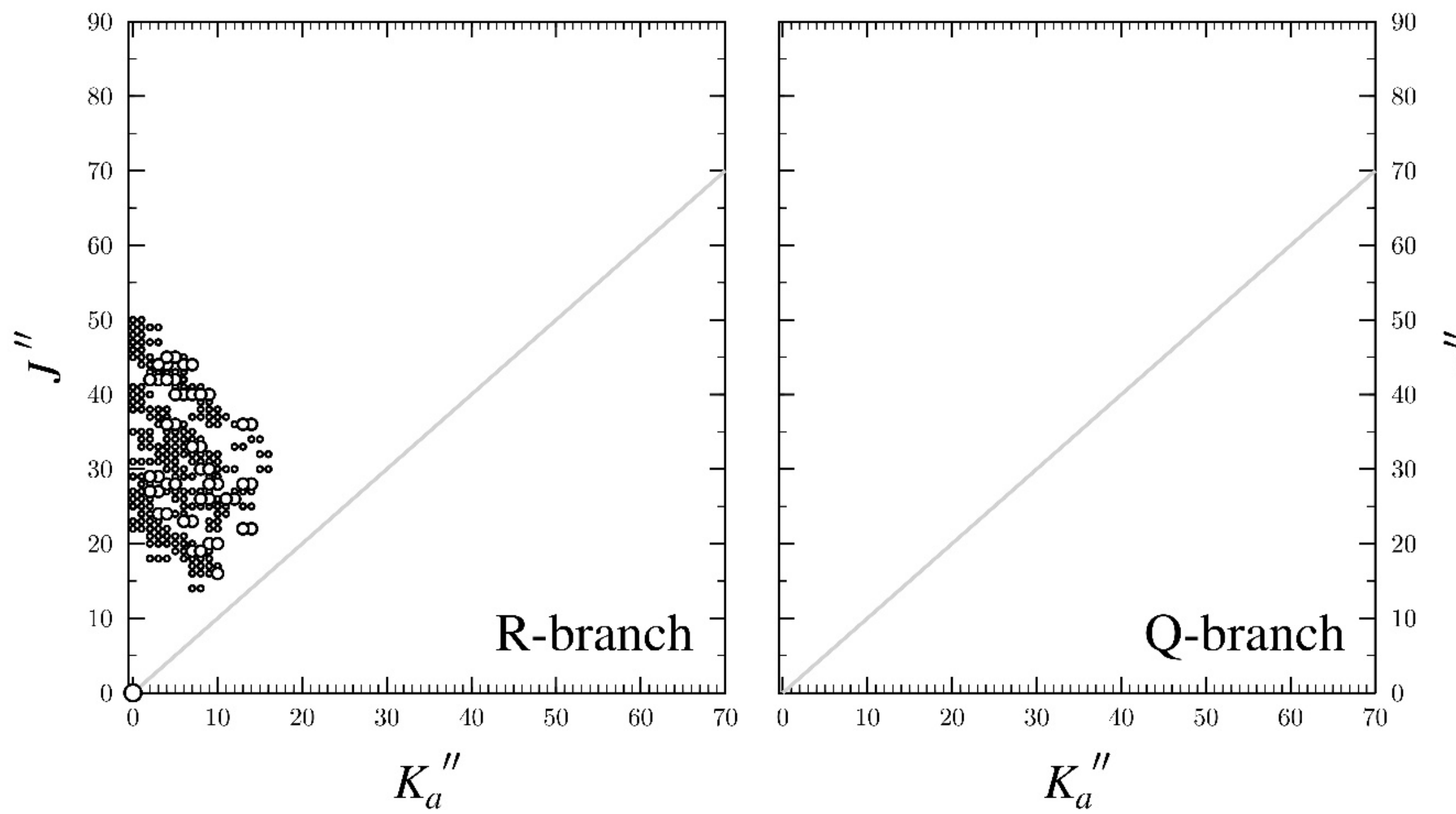

Fig. S35. Data distribution plot for the least-squares fit of spectroscopic data for the vibrational ground state, [2,3,4-^{2}H, 5-^{13}C]-furan isotopologue. The size of the outlined circle is proportional to the value of $|(f_{obs.} - f_{calc.})/\delta f|$, where δf is the frequency measurement uncertainty, and all values represented are less than or equal to 3.

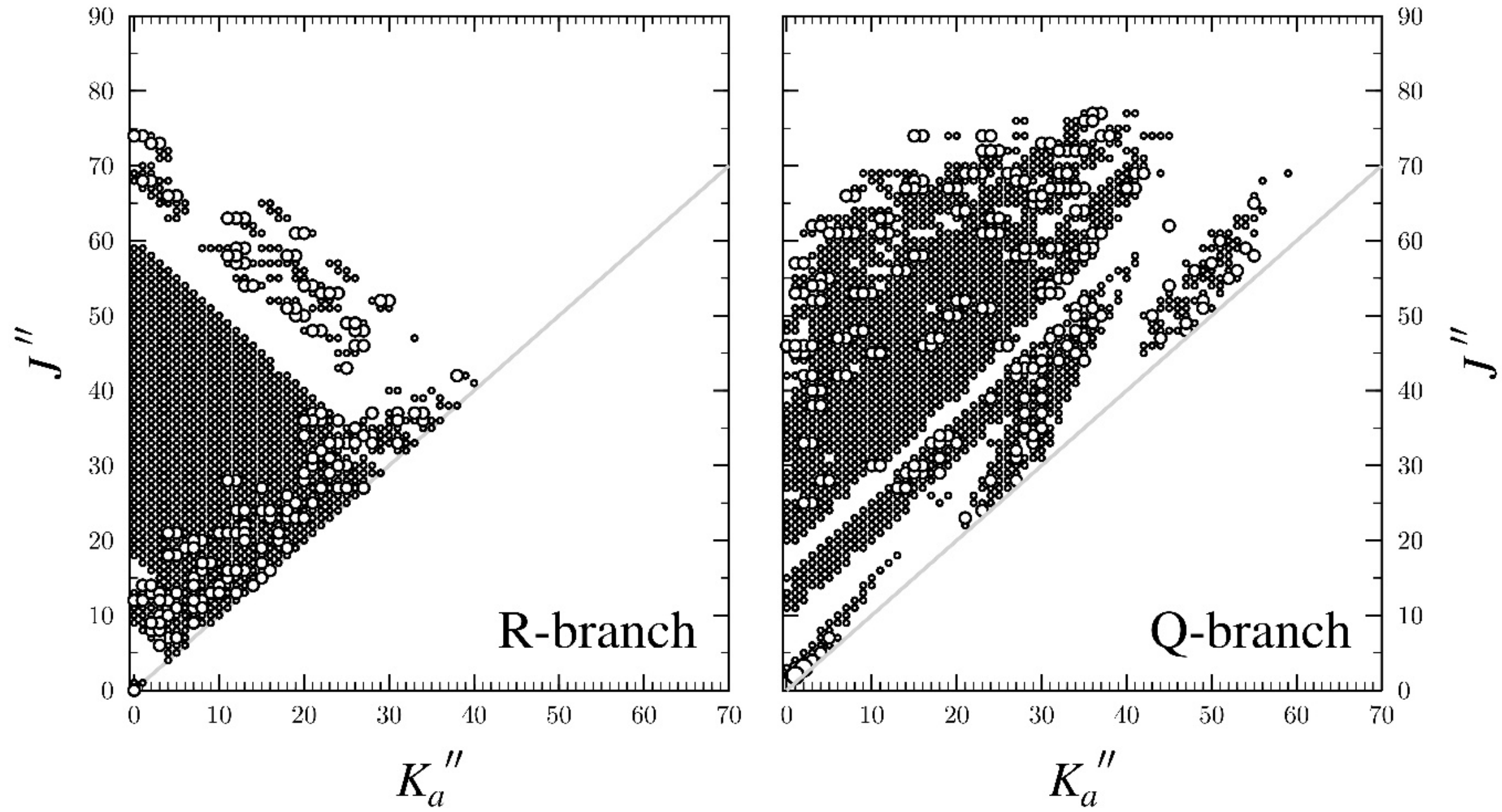

Fig. S36. Data distribution plot for the least-squares fit of spectroscopic data for the vibrational ground state, [2,3,5-^{2}H]-furan isotopologue. The size of the outlined circle is proportional to the value of $|(f_{obs.} - f_{calc.})/\delta f|$, where δf is the frequency measurement uncertainty, and all values represented are less than or equal to 3.

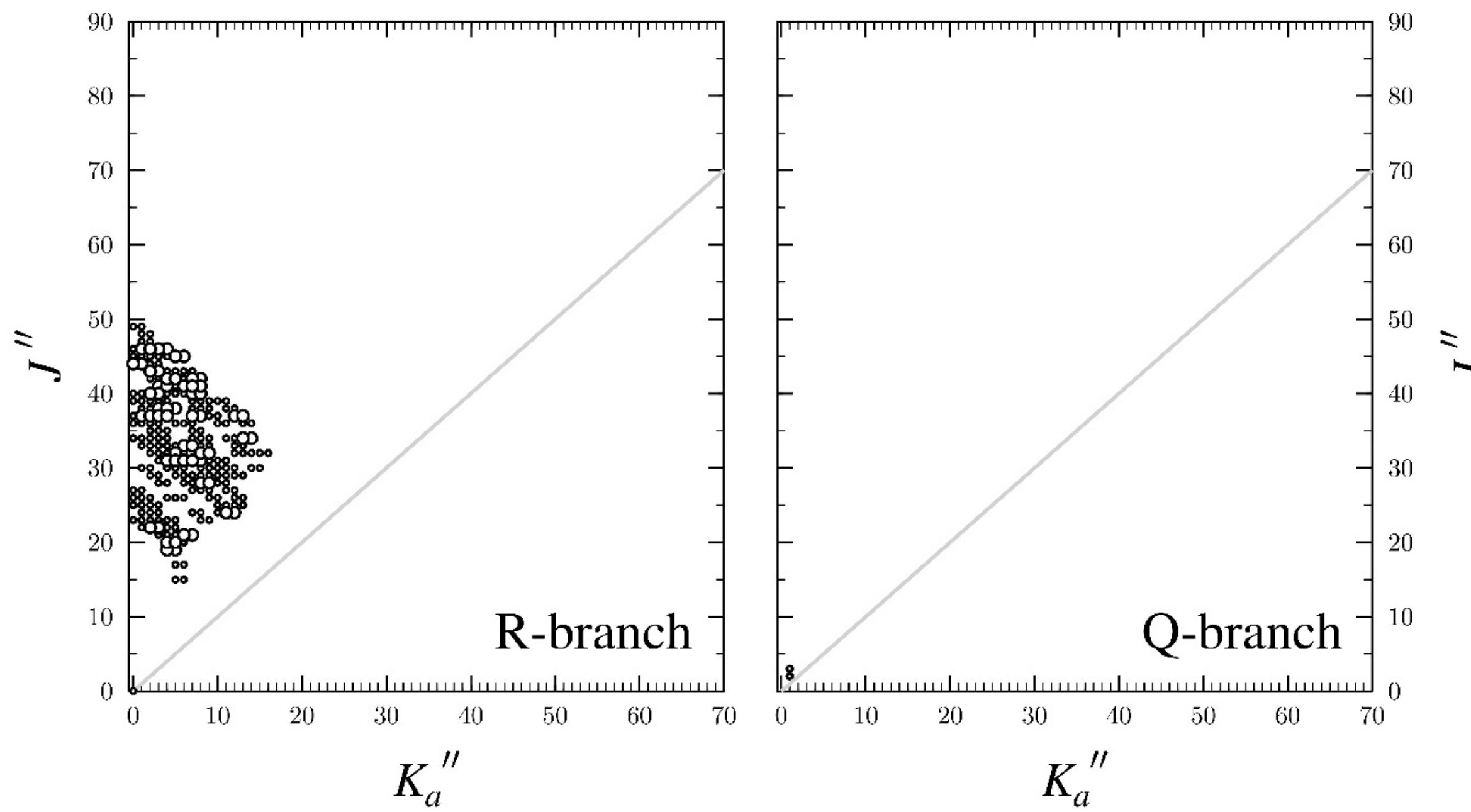

Fig. S37. Data distribution plot for the least-squares fit of spectroscopic data for the vibrational ground state, [2,3,5-^{2}H, 2-^{13}C]-furan isotopologue. The size of the outlined circle is proportional to the value of $|(f_{obs.} - f_{calc.})/\delta f|$, where δf is the frequency measurement uncertainty, and all values represented are less than or equal to 3.

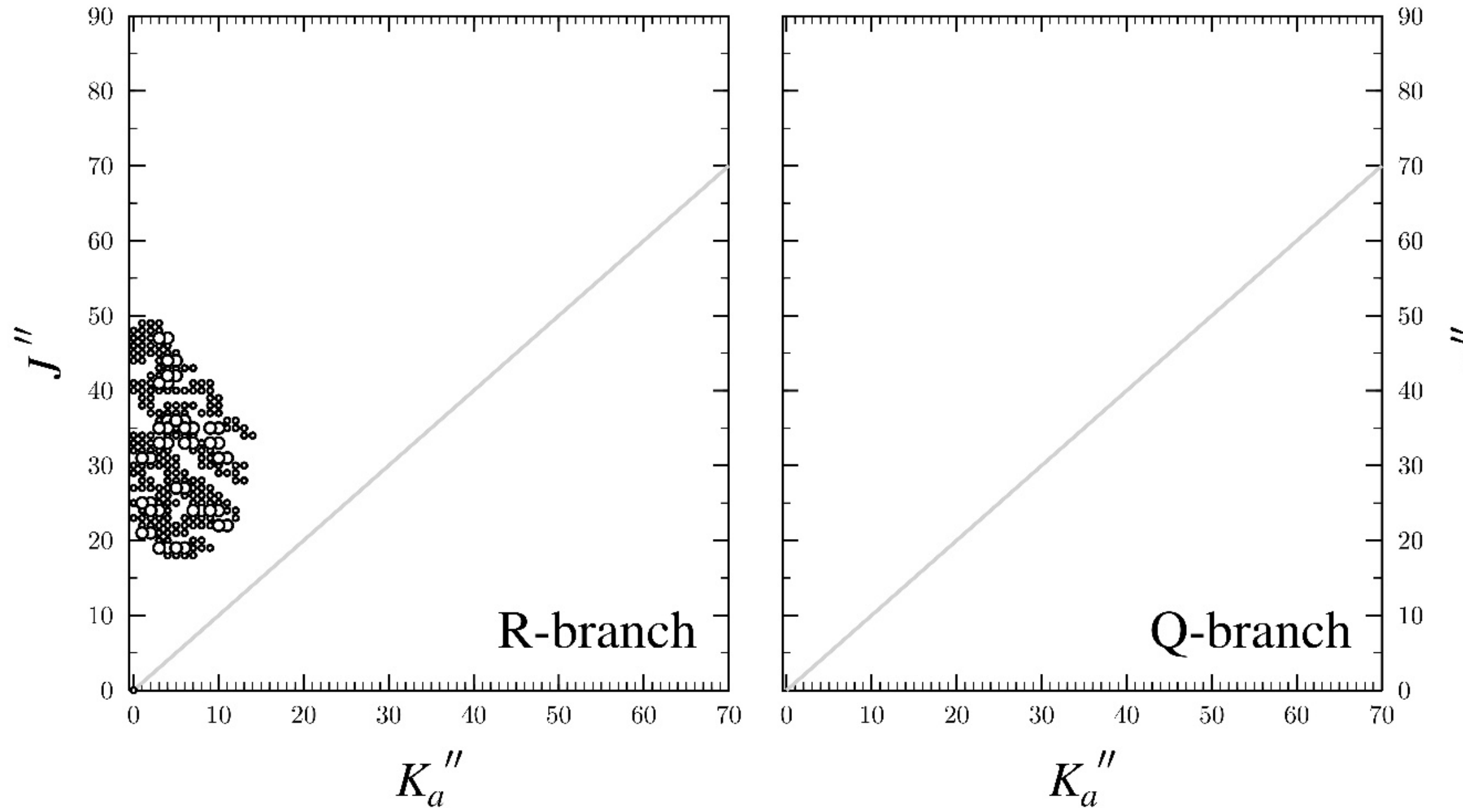

Fig. S38. Data distribution plot for the least-squares fit of spectroscopic data for the vibrational ground state, [2,3,5-^{2}H, 3-^{13}C]-furan isotopologue. The size of the outlined circle is proportional to the value of $|(f_{obs.} - f_{calc.})/\delta f|$, where δf is the frequency measurement uncertainty, and all values represented are less than or equal to 3.

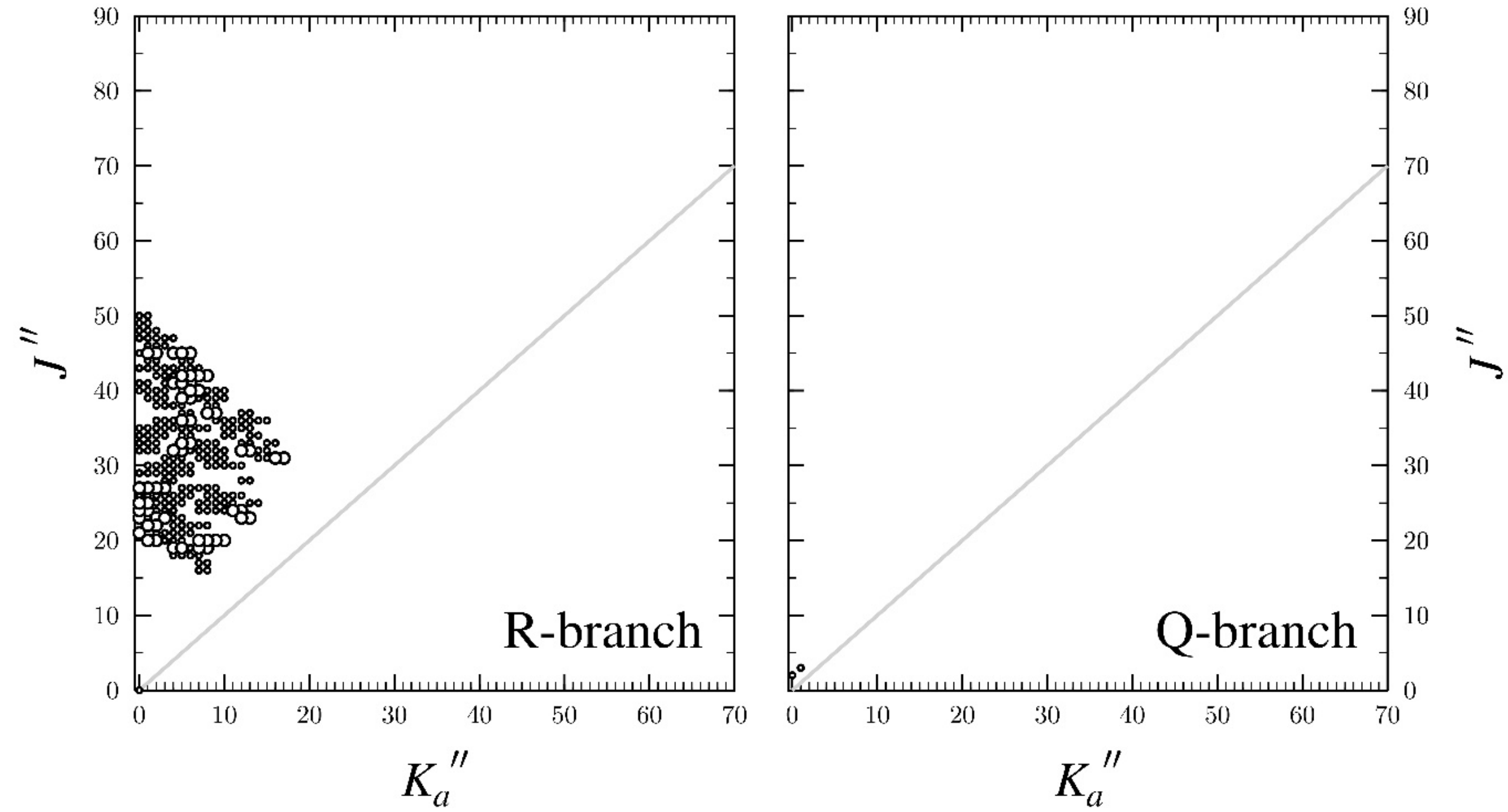

Fig. S39. Data distribution plot for the least-squares fit of spectroscopic data for the vibrational ground state, [2,3,5-^{2}H, 4-^{13}C]-furan isotopologue. The size of the outlined circle is proportional to the value of $|(f_{obs.} - f_{calc.})/\delta f|$, where δf is the frequency measurement uncertainty, and all values represented are less than or equal to 3.

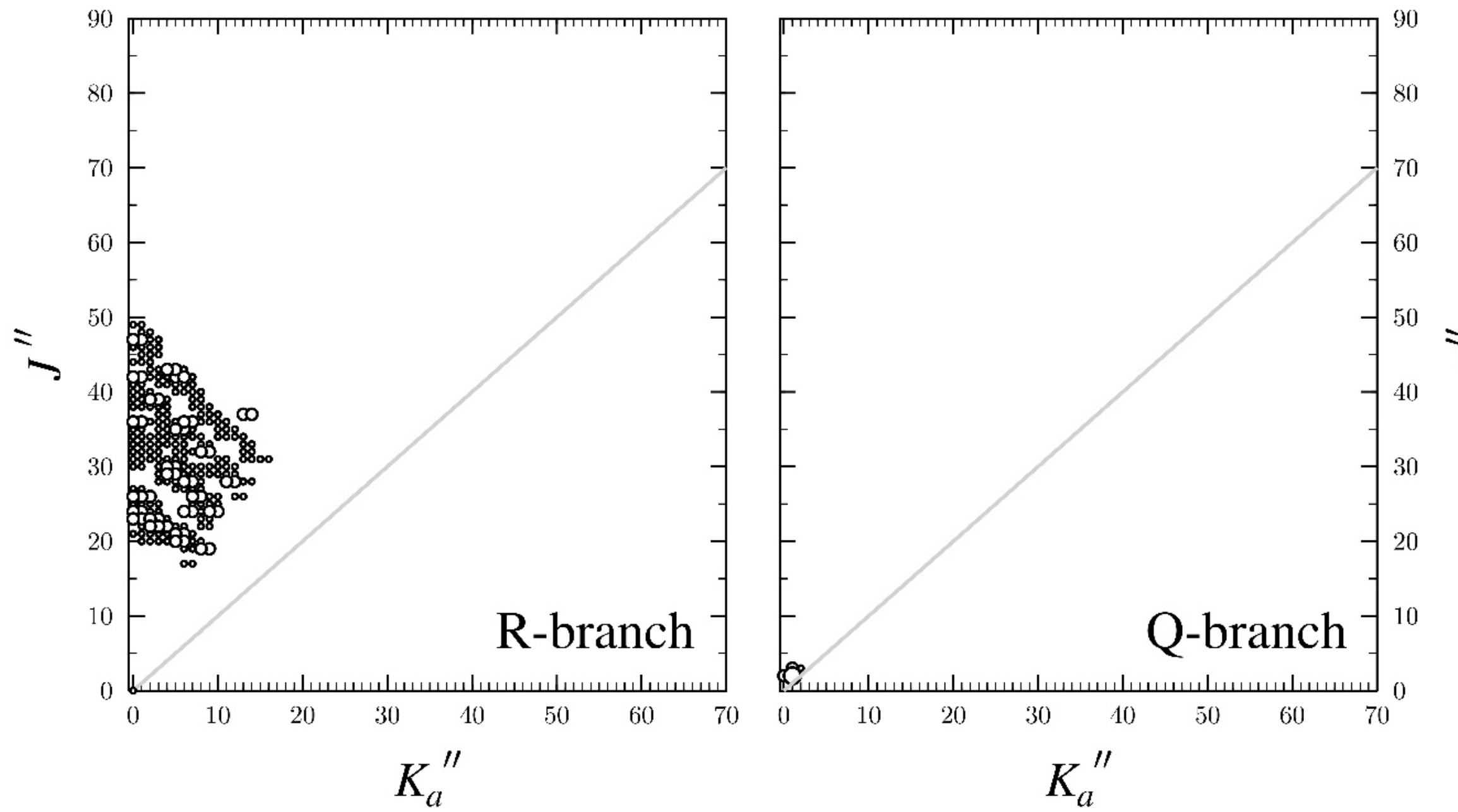

Fig. S40. Data distribution plot for the least-squares fit of spectroscopic data for the vibrational ground state, [2,3,5-^{2}H, 5-^{13}C]-furan isotopologue. The size of the outlined circle is proportional to the value of $|(f_{obs.} - f_{calc.})/\delta f|$, where δf is the frequency measurement uncertainty, and all values represented are less than or equal to 3.

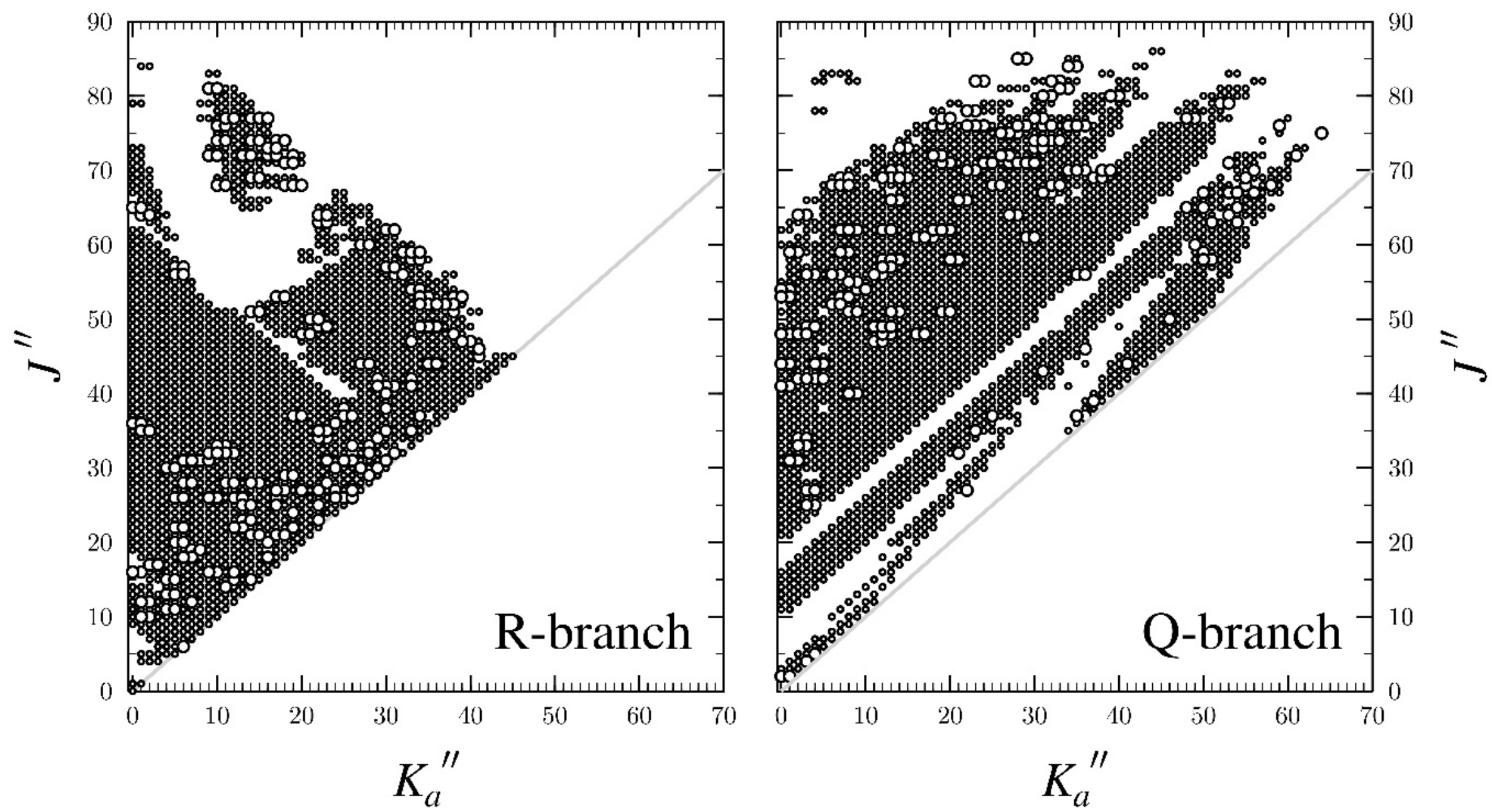

Fig. S41. Data distribution plot for the least-squares fit of spectroscopic data for the vibrational ground state, [2,3,4,5-^{2}H]-furan isotopologue. The size of the outlined circle is proportional to the value of $|(f_{obs.} - f_{calc.})/\delta f|$, where δf is the frequency measurement uncertainty, and all values represented are less than or equal to 3.

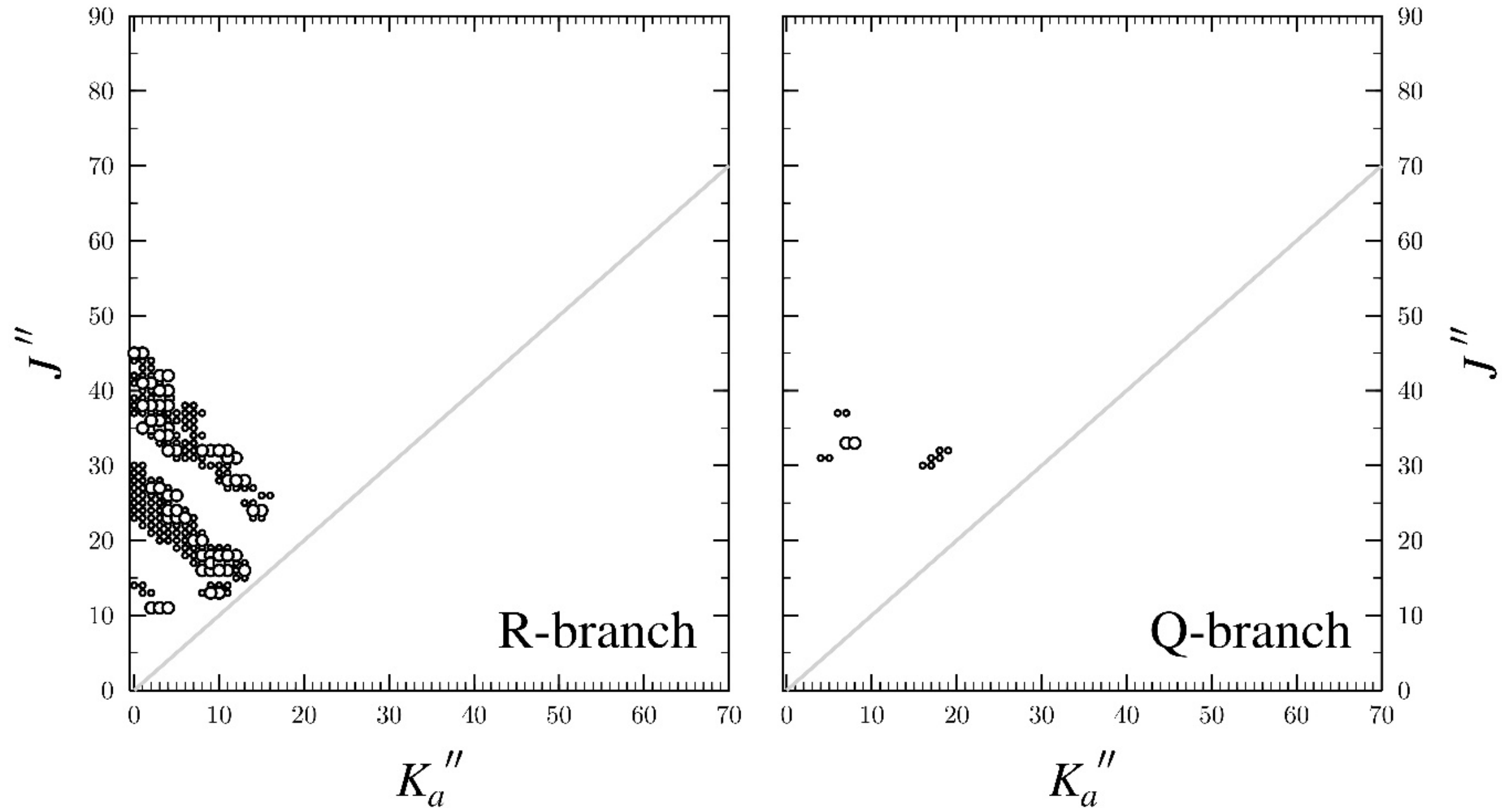

Fig. S42. Data distribution plot for the least-squares fit of spectroscopic data for the vibrational ground state, [2,3,4,5-^{2}H, ^{18}O]-furan isotopologue. The size of the outlined circle is proportional to the value of $|(f_{obs.} - f_{calc.})/\delta f|$, where δf is the frequency measurement uncertainty, and all values represented are less than or equal to 3.

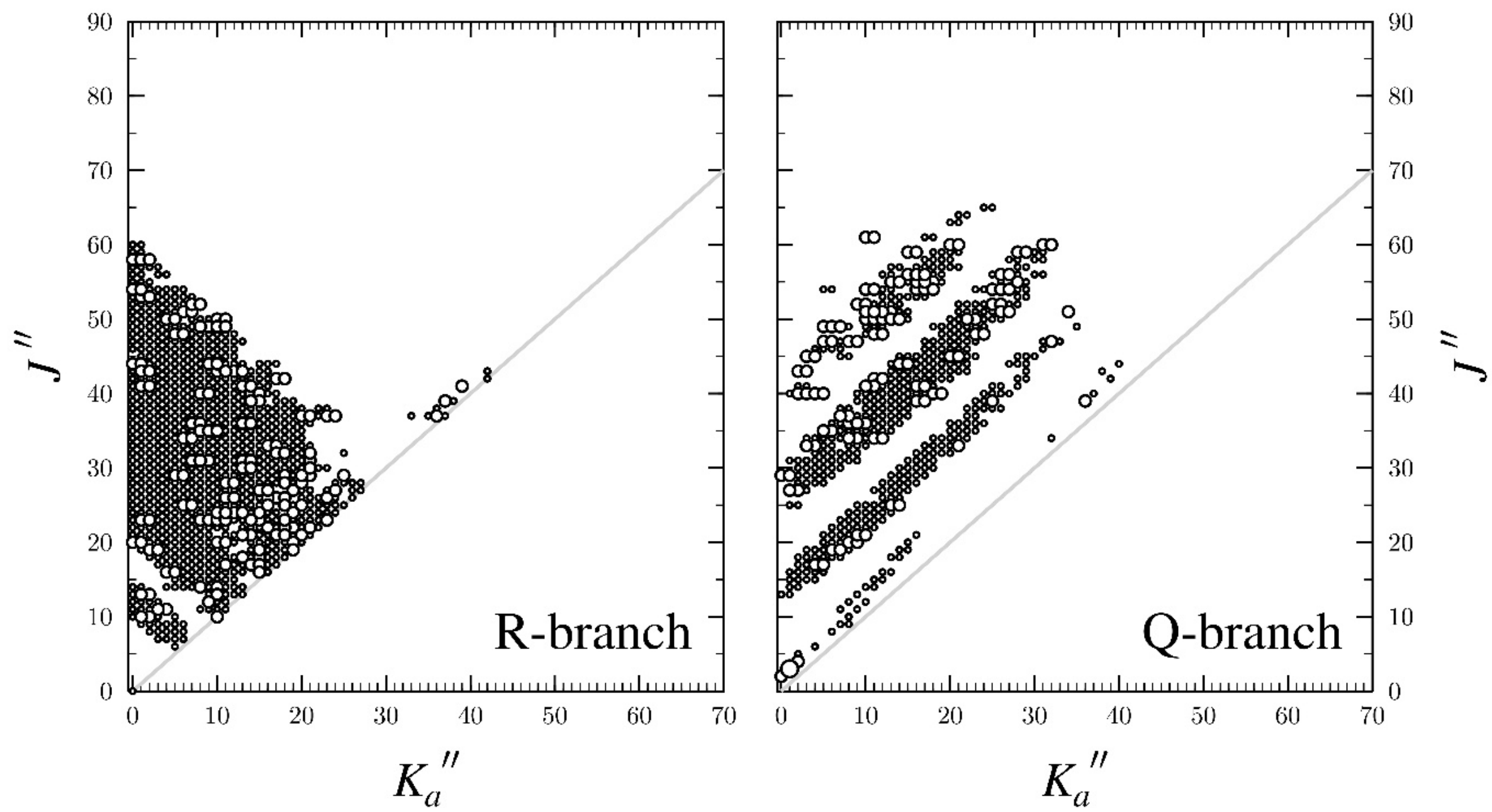

Fig. S43. Data distribution plot for the least-squares fit of spectroscopic data for the vibrational ground state, [2,3,4,5-^{2}H, 2-^{13}C]-furan isotopologue. The size of the outlined circle is proportional to the value of $|(f_{obs.} - f_{calc.})/\delta f|$, where δf is the frequency measurement uncertainty, and all values represented are less than or equal to 3.

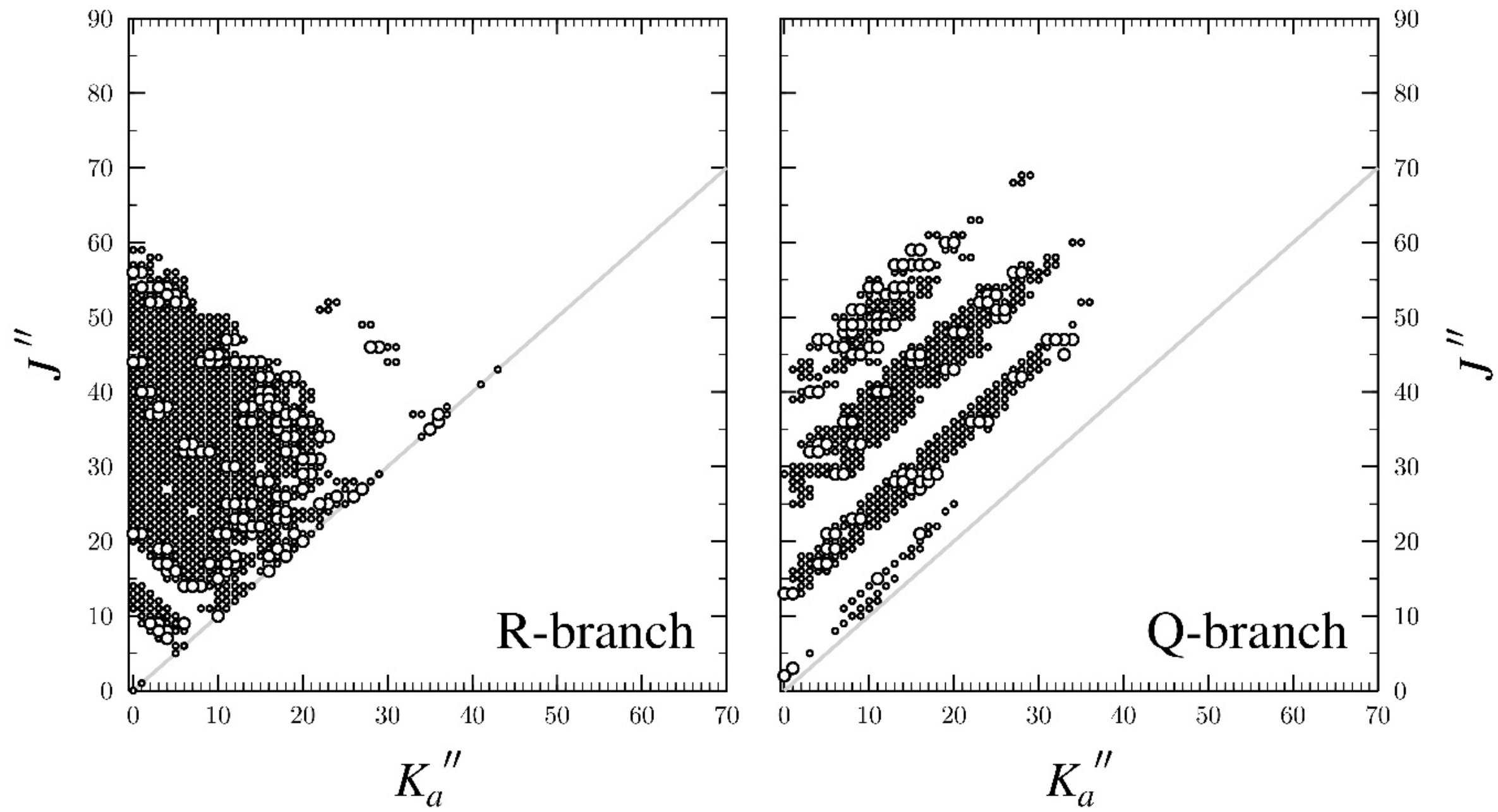

Fig. S44. Data distribution plot for the least-squares fit of spectroscopic data for the vibrational ground state, [2,3,4,5-^{2}H, 3-^{13}C]-furan isotopologue. The size of the outlined circle is proportional to the value of $|(f_{obs.} - f_{calc.})/\delta f|$, where δf is the frequency measurement uncertainty, and all values represented are less than or equal to 3.

r_e vs. r_e^{SE} Principal Axis Rotation Analysis

To compare the angles of rotation of the computed equilibrium (r_e) structure and semi-experimental equilibrium (r_e^{SE}) structure, the atomic masses and their coordinates in both structures are used to calculate the center-of-mass coordinates for each isotopologue, based on a reference set of input coordinates.

For a planar molecule lying in the *x*,*y* plane, the atomic coordinates (x_i, y_i, z_i) and exact mass (m_i) of each atom, *i*, are used to calculate the following moments and products of inertia of the structures (equation 2.3, page 12, Gordy and Cook).[1]

$$I_{xx} = \sum_i m_i y_i^2 \tag{S16}$$

$$I_{yy} = \sum_i m_i x_i^2 \tag{S17}$$

$$I_{xy} = -\sum_i m_i x_i y_i \tag{S18}$$

When the I_{xx} and I_{yy} values are mapped onto I_{aa} and I_{bb}, they must follow the convention that $I_{aa} < I_{bb}$, *i.e.*, $A > B$. The *a*- and *b*-axes moments of inertia are then used to calculate the angle, θ_i, for each isotopologue using equation 7 from Demaison *et al*:[25]

$$\theta_i = \frac{1}{2}\tan^{-1}\frac{2I_{ab}}{I_{aa} - I_{bb}} \tag{S19}$$

This angle, θ_i, is the angle of rotation between the input coordinate system and the principal axis system. This angle can vary depending on the input coordinates, and does not have intrinsic meaning, though it allows for comparison. To calculate the relative angle (θ_r) for isotopologue *i* with respect to the normal isotopologue, Eq. (S19) is used, where θ_n is the value of θ_i for the normal isotopologue.

$$\theta_r = \theta_i - \theta_n \tag{S20}$$

This relative angle is the degree of rotation between the principal axes of the analyzed isotopologue and the normal isotopologue, Eq. (S20). This analysis is completed for both the r_e and r_e^{SE} structures for all isotopologues. If there is a large difference between the θ_r rotations between r_e and r_e^{SE} structures, the difference in rotation between the two structures may be problematic and indicate an incorrect calculation/application of the vibration-rotation interaction corrections.

Table S6. Rotational constant residuals ($B_e^{xrefit} - B_e^{SE}$).

Isotopologue	r_e^{SE}**(initial)** $A_{e,\mathrm{residual}}$ (MHz)	$B_{e,\mathrm{residual}}$ (MHz)	$C_{e,\mathrm{residual}}$ (MHz)	r_e^{SE}**(final)** $A_{e,\mathrm{residual}}$ (MHz)	$B_{e,\mathrm{residual}}$ (MHz)	$C_{e,\mathrm{residual}}$ (MHz)
furan	−0.021	−0.045	0.037	−0.065	−0.115	0.047
[^{18}O]	−0.039	−0.059	0.027	−0.092	−0.105	0.032
[2-^{13}C]	−1.168	1.065	0.027	−0.307	0.077	0.033
[3-^{13}C]	−0.177	0.080	0.028	−0.112	−0.108	0.035
[2-^{2}H]	−0.141	0.088	0.032	−0.135	−0.033	0.045
[2-^{2}H, 2-^{13}C]	−0.076	−0.005	0.022	−0.111	−0.102	0.030
[2-^{2}H, 3-^{13}C]	−0.186	0.142	0.021	−0.151	−0.019	0.029
[2-^{2}H, 4-^{13}C]	0.045	−0.081	0.025	0.034	−0.197	0.033
[2-^{2}H, 5-^{13}C]	−0.078	0.003	0.022	−0.125	−0.080	0.030
[3-^{2}H]	−0.142	0.037	0.023	−0.143	−0.046	0.040
[3-^{2}H, 2-^{13}C]	−0.279	0.132	0.014	−0.255	0.005	0.025
[3-^{2}H, 3-^{13}C]	−0.089	−0.021	0.015	−0.111	−0.098	0.027
[3-^{2}H, 4-^{13}C]	−0.091	0.012	0.013	−0.107	−0.068	0.026
[3-^{2}H, 5-^{13}C]	−0.359	0.208	0.014	−0.345	0.093	0.026
[2,3-^{2}H]	−0.415	0.318	0.019	−0.158	−0.021	0.036
[2,3-^{2}H, 2-^{13}C]	−0.140	0.011	0.011	−0.098	−0.130	0.023
[2,3-^{2}H, 3-^{13}C]	−0.326	0.205	0.010	−0.179	−0.037	0.023
[2,3-^{2}H, 4-^{13}C]	−0.837	0.707	0.009	−0.189	−0.034	0.022
[2,3-^{2}H, 5-^{13}C]	−0.234	0.106	0.012	−0.152	−0.073	0.023
[2,4-^{2}H]	−0.094	0.014	0.020	−0.159	−0.021	0.039
[2,4-^{2}H, 2-^{13}C]	−0.075	−0.031	0.013	−0.149	−0.073	0.026
[2,4-^{2}H, 3-^{13}C]	−0.126	0.020	0.011	−0.183	−0.032	0.025
[2,4-^{2}H, 4-^{13}C]	−0.068	−0.035	0.014	−0.152	−0.066	0.029
[2,4-^{2}H, 5-^{13}C]	−0.085	−0.017	0.011	−0.151	−0.066	0.024
[2,5-^{2}H]	−0.018	−0.037	0.026	−0.099	−0.065	0.040
[2,5-^{2}H, 2-^{13}C]	−0.049	−0.054	0.017	−0.143	−0.087	0.026
[2,5-^{2}H, 3-^{13}C]	−0.033	−0.047	0.018	−0.117	−0.085	0.028
[3,4-^{2}H]	−0.125	−0.026	0.009	−0.133	−0.069	0.030
[3,4-^{2}H, 2-^{13}C]	−0.141	−0.034	0.002	−0.141	−0.104	0.018
[3,4-^{2}H, 3-^{13}C]	−0.135	−0.047	0.003	−0.139	−0.107	0.019
[2,3,4-^{2}H]	−0.223	0.092	0.007	−0.144	−0.047	0.028
[2,3,4-^{2}H, 2-^{13}C]	−0.213	0.056	0.000	−0.153	−0.081	0.016
[2,3,4-^{2}H, 3-^{13}C]	−0.273	0.114	0.001	−0.175	−0.056	0.017
[2,3,4-^{2}H, 4-^{13}C]	−0.165	0.018	0.000	−0.137	−0.083	0.017
[2,3,4-^{2}H, 5-^{13}C]	−0.306	0.152	−0.001	−0.176	−0.054	0.015
[2,3,5-^{2}H]	−0.092	0.005	0.015	−0.125	−0.051	0.032
[2,3,5-^{2}H, 2-^{13}C]	−0.082	−0.036	0.007	−0.138	−0.084	0.020
[2,3,5-^{2}H, 3-^{13}C]	−0.110	0.001	0.009	−0.149	−0.061	0.022
[2,3,5-^{2}H, 4-^{13}C]	−0.115	−0.001	0.008	−0.150	−0.065	0.021
[2,3,5-^{2}H, 5-^{13}C]	−0.111	−0.017	0.007	−0.163	−0.068	0.020
[2,3,4,5-^{2}H]	−0.019	−0.106	0.003	−0.074	−0.113	0.023
[2,3,4,5-^{2}H, ^{18}O]	−0.025	−0.120	−0.002	−0.069	−0.133	0.018
[2,3,4,5-^{2}H, 2-^{13}C]	−0.037	−0.116	−0.004	−0.098	−0.131	0.011
[2,3,4,5-^{2}H, 3-^{13}C]	−0.086	−0.064	−0.003	−0.107	−0.116	0.013
**Average ($\bar{x}$)**	−0.172	0.059	0.013	−0.141	−0.068	0.026
**Std. Dev. (*s*)**	0.211	0.207	0.010	0.059	0.051	0.008

Relation of the observable ground vibrational state and equilibrium rotational constants for an asymmetric-top molecule

The vibration-rotation interaction correction that relates the ground vibrational state ($B_{v1\ v2\ v3\ldots}$) and equilibrium rotational constants (B_e) involves a first-order α term and higher order terms, such as the γ and ε terms. Shown in Eq. (S21) is the vibration-rotation correction utilizing only the α and γ terms,[26] highlighting the lower impact of successive correction terms. Modern computational software calculates the α term, which is then implemented into the vibration-rotation interaction correction as a reasonable approximation of the true correction, but no published software implements any higher-order terms. Implementation of further terms, such as the γ term, would afford a more-accurate vibration-rotation interaction correction, and may further improve the inertial defects and bring them closer to their true value of 0.

$$B_{\nu 1\ \nu 2\ \nu 3} = B_e - \sum_{i}^{3N-6} \alpha_i^B \left(\nu_i + \frac{1}{2}\right) + \sum_{ij}^{3N-6} \gamma_{ij}^B \left(\nu_i + \frac{1}{2}\right)\left(\nu_j + \frac{1}{2}\right) \qquad \text{(S21)}$$

Table S7. Rotational constant residuals ($B_e^{xrefit} – B_e^{\mathrm{SE}}$) vs ($B_e^{\mathrm{CCSD(T)}} – B_e^{\mathrm{SE}}$).

Isotopologue	r_e^{SE}**(final)**			**CCSD(T)/cc-pCVTZ**		
	$A_{e,\mathrm{residual}}$ (MHz)	$B_{e,\mathrm{residual}}$ (MHz)	$C_{e,\mathrm{residual}}$ (MHz)	$A_{e,\mathrm{residual}}$ (MHz)	$B_{e,\mathrm{residual}}$ (MHz)	$C_{e,\mathrm{residual}}$ (MHz)
furan	−0.065	−0.115	0.047	−28.675	−53.552	−21.013
[^{18}O]	−0.092	−0.105	0.032	−28.693	−51.608	−20.783
[2-^{13}C]	−0.307	0.077	0.033	−38.461	−42.916	−20.665
[3-^{13}C]	−0.112	−0.108	0.035	−31.645	−49.609	−20.805
[2-^{2}H]	−0.135	−0.033	0.045	−48.334	−30.685	−19.755
[2-^{2}H, 2-^{13}C]	−0.111	−0.102	0.030	−49.166	−29.190	−19.462
[2-^{2}H, 3-^{13}C]	−0.151	−0.019	0.029	−48.450	−29.515	−19.580
[2-^{2}H, 4-^{13}C]	0.034	−0.197	0.033	−45.391	−32.692	−19.560
[2-^{2}H, 5-^{13}C]	−0.125	−0.080	0.030	−50.476	−27.714	−19.431
[3-^{2}H]	−0.143	−0.046	0.040	−35.417	−42.298	−19.935
[3-^{2}H, 2-^{13}C]	−0.255	0.005	0.025	−35.901	−40.962	−19.627
[3-^{2}H, 3-^{13}C]	−0.111	−0.098	0.027	−35.779	−41.184	−19.761
[3-^{2}H, 4-^{13}C]	−0.107	−0.068	0.026	−32.706	−44.069	−19.753
[3-^{2}H, 5-^{13}C]	−0.345	0.093	0.026	−37.791	−39.153	−19.607
[2,3-^{2}H]	−0.158	−0.021	0.036	−41.558	−32.844	−18.802
[2,3-^{2}H, 2-^{13}C]	−0.098	−0.130	0.023	−46.675	−27.066	−18.540
[2,3-^{2}H, 3-^{13}C]	−0.179	−0.037	0.023	−37.897	−35.741	−18.654
[2,3-^{2}H, 4-^{13}C]	−0.189	−0.034	0.022	−46.516	−27.095	−18.632
[2,3-^{2}H, 5-^{13}C]	−0.152	−0.073	0.023	−44.192	−29.461	−18.495
[2,4-^{2}H]	−0.159	−0.021	0.039	−41.994	−32.944	−18.769
[2,4-^{2}H, 2-^{13}C]	−0.149	−0.073	0.026	−43.019	−31.300	−18.490
[2,4-^{2}H, 3-^{13}C]	−0.183	−0.032	0.025	−40.655	−33.364	−18.613
[2,4-^{2}H, 4-^{13}C]	−0.152	−0.066	0.029	−41.270	−32.992	−18.604
[2,4-^{2}H, 5-^{13}C]	−0.151	−0.066	0.024	−42.800	−31.295	−18.482
[2,5-^{2}H]	−0.099	−0.065	0.040	−51.244	−24.613	−18.610
[2,5-^{2}H, 2-^{13}C]	−0.143	−0.087	0.026	−51.148	−24.097	−18.334
[2,5-^{2}H, 3-^{13}C]	−0.117	−0.085	0.028	−50.031	−24.894	−18.443
[3,4-^{2}H]	−0.133	−0.069	0.030	−27.313	−46.160	−18.970
[3,4-^{2}H, 2-^{13}C]	−0.141	−0.104	0.018	−27.042	−45.644	−18.677
[3,4-^{2}H, 3-^{13}C]	−0.139	−0.107	0.019	−27.691	−45.124	−18.814
[2,3,4-^{2}H]	−0.144	−0.047	0.028	−35.113	−35.501	−17.914
[2,3,4-^{2}H, 2-^{13}C]	−0.153	−0.081	0.016	−37.661	−32.341	−17.665
[2,3,4-^{2}H, 3-^{13}C]	−0.175	−0.056	0.017	−32.011	−37.916	−17.781
[2,3,4-^{2}H, 4-^{13}C]	−0.137	−0.083	0.017	−34.923	−35.080	−17.768
[2,3,4-^{2}H, 5-^{13}C]	−0.176	−0.054	0.015	−38.009	−31.847	−17.641
[2,3,5-^{2}H]	−0.125	−0.051	0.032	−44.665	−27.012	−17.736
[2,3,5-^{2}H, 2-^{13}C]	−0.138	−0.084	0.020	−45.578	−25.460	−17.490
[2,3,5-^{2}H, 3-^{13}C]	−0.149	−0.061	0.022	−42.953	−28.015	−17.594
[2,3,5-^{2}H, 4-^{13}C]	−0.150	−0.065	0.021	−44.635	−26.216	−17.587
[2,3,5-^{2}H, 5-^{13}C]	−0.163	−0.068	0.020	−45.076	−26.026	−17.475
[2,3,4,5-^{2}H]	−0.074	−0.113	0.023	−44.205	−23.601	−16.948
[2,3,4,5-^{2}H, ^{18}O]	−0.069	−0.133	0.018	−42.831	−23.616	−16.803
[2,3,4,5-^{2}H, 2-^{13}C]	−0.098	−0.131	0.011	−43.981	−23.237	−16.714
[2,3,4,5-^{2}H, 3-^{13}C]	−0.107	−0.116	0.013	−42.568	−24.608	−16.821
**Average ($\bar{x}$)**	−0.141	−0.068	0.026	−40.5	−33.6	−18.7
**Std. Dev. (*s*)**	0.059	0.051	0.008	6.9	8.3	1.1

(a)

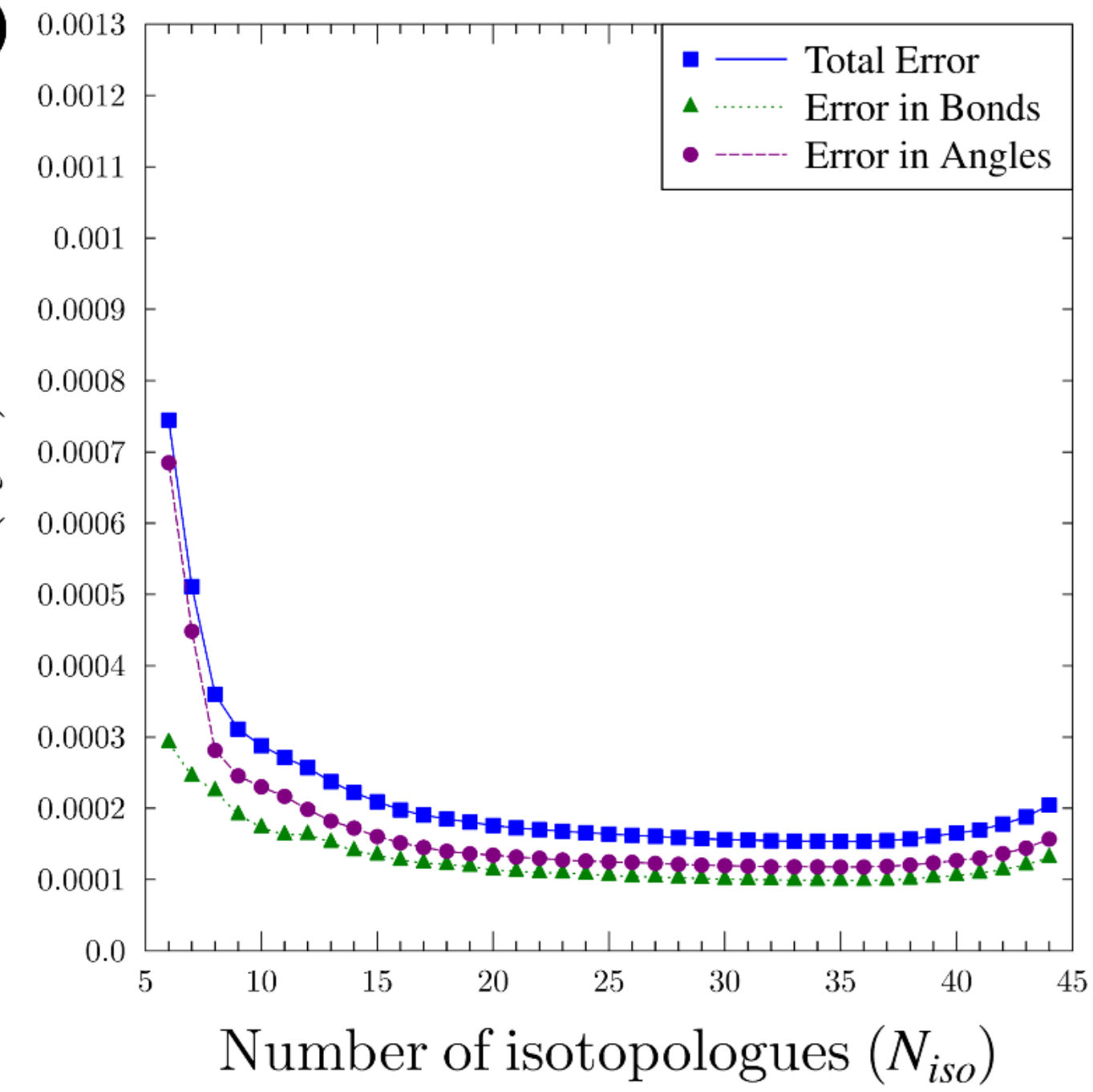

N	r_e^{SE}(initial) Isotopologue Added	N	r_e^{SE}(initial) Isotopologue Added
6	Core Set	26	[2-2H, 4-13C]
7	[2,3,4-2H, 5-13C]	27	[2,5-2H, 2-13C]
8	[3,4-2H, 3-13C]	28	[2,5-2H]
9	[3,4-2H, 2-13C]	29	[2,3,5-2H, 2-13C]
10	[2,3-2H, 4-13C]	30	[2,3,5-2H, 3-13C]
11	[3-2H, 4-13C]	31	[3,4-2H]
12	[2,3,4,5-2H, 18O]	32	[2,3,5-2H, 5-13C]
13	[2,3,4-2H, 2-13C]	33	[2,3,5-2H, 4-13C]
14	[2,3,4-2H, 4-13C]	34	[2,3-2H, 2-13C]
15	[2,4-2H, 5-13C]	35	[2,3,5-2H]
16	[2,4-2H, 3-13C]	36	[2,4-2H]
17	[2,4-2H, 2-13C]	37	[2-2H, 3-13C]
18	[2-2H, 2-13C]	38	[2,3,4-2H]
19	[2-2H, 5-13C]	39	[2,3,4-2H, 3-13C]
20	[2,5-2H, 3-13C]	40	[3-2H, 2-13C]
21	[3-2H, 3-13C]	41	[2,3-2H, 5-13C]
22	[2,4-2H, 4-13C]	42	[3-2H, 5-13C]
23	[2,3,4,5-2H, 2-13C]	43	[2,3-2H, 3-13C]
24	[2,3,4,5-2H]	44	[2,3-2H]
25	[2,3,4,5-2H, 3-13C]		

(b)

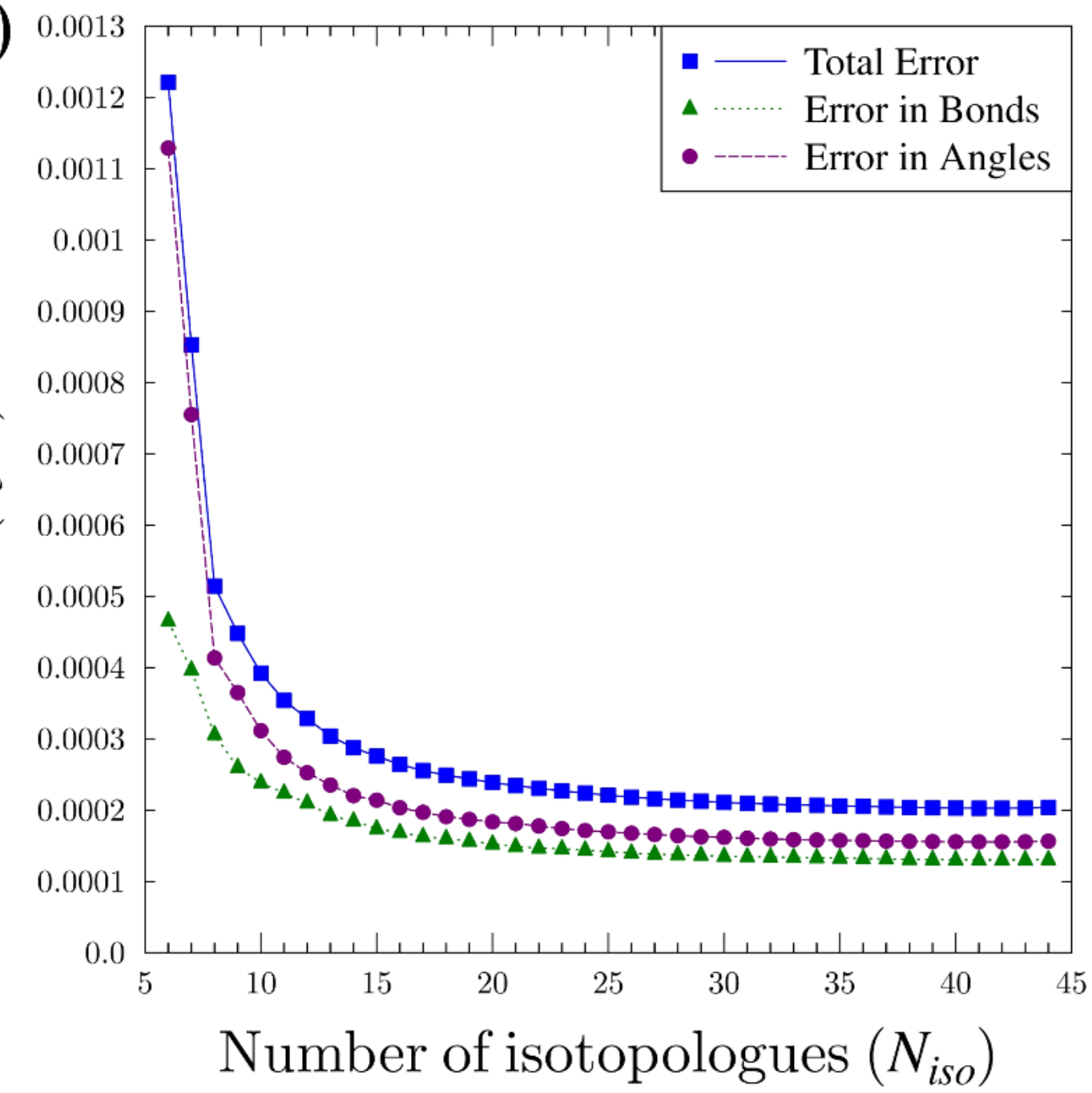

N	r_e^{SE}(final) Isotopologue Added	N	r_e^{SE}(final) Isotopologue Added
6	Core Set	26	[2,3-^{2}H, 2-^{13}C]
7	[2,3,4-^{2}H, 5-^{13}C]	27	[3-^{2}H, 3-^{13}C]
8	[2,3,4,5-^{2}H, 3-^{13}C]	28	[2,3,5-^{2}H, 2-^{13}C]
9	[2,3,4,5-^{2}H, ^{18}O]	29	[2,3,5-^{2}H, 5-^{13}C]
10	[2,4-^{2}H, 3-^{13}C]	30	[2,3,5-^{2}H, 4-^{13}C]
11	[2,4-^{2}H, 5-^{13}C]	31	[2,5-^{2}H, 2-^{13}C]
12	[3-^{2}H, 4-^{13}C]	32	[2,5-^{2}H]
13	[3,4-^{2}H, 2-^{13}C]	33	[2,3,4-^{2}H, 3-^{13}C]
14	[2-^{2}H, 5-^{13}C]	34	[3,4-^{2}H]
15	[2,5-^{2}H, 3-^{13}C]	35	[2,3,4,5-^{2}H]
16	[2,3,4,5-^{2}H, 2-^{13}C]	36	[3-^{2}H, 2-^{13}C]
17	[2,3,4-^{2}H, 4-^{13}C]	37	[2,3-^{2}H, 3-^{13}C]
18	[2-^{2}H, 2-^{13}C]	38	[2-^{2}H, 4-^{13}C]
19	[2,3,4-^{2}H, 2-^{13}C]	39	[2,3-^{2}H, 4-^{13}C]
20	[2,4-^{2}H, 4-^{13}C]	40	[2,3,4-^{2}H]
21	[2-^{2}H, 3-^{13}C]	41	[2,3,5-^{2}H]
22	[2,4-^{2}H, 2-^{13}C]	42	[2,4-^{2}H]
23	[3,4-^{2}H, 3-^{13}C]	43	[2,3-^{2}H]
24	[2,3-^{2}H, 5-^{13}C]	44	[3-^{2}H, 5-^{13}C]
25	[2,3,5-^{2}H, 3-^{13}C]		

Fig. S45. Plot of structural parameter uncertainty values (δr_e^{SE}) for (a) the r_e^{SE}(initial) geometry all three rotational constants for 40 isotopologues and only the C rotational constant for [2-^{13}C]-, [2,3-^{2}H, 4-^{13}C]-, [2,4-^{2}H]-, and [2,4-^{2}H, 4-^{13}C]-furan, and (b) the r_e^{SE}(final) geometry using all three rotational constants for 44 isotopologues. Plots depict the 2σ parameter uncertainties as a function of the number of isotopologues (N_{iso}) incorporated into the structure determination using vibration-rotation interaction corrsections compued at the CCSD(T)/cc-pCVTZ or r_e^{SE}(intial) geometries, respectively. Isotopologue ordering along the *x*-axis is provided in the tables on the right.

Furan-d_x Synthetic Details

Scheme S1 shows the H/D exchange method employed. To a stainless-steel vessel (Swagelok double-ended sample cylinder, 150 mL, model 304L-HDF-150, 1/4-inch female NPT connections, Figure S46) were added a magnetic stir bar, 10.33 g of furan (152 mmol, 1 eq), 11.97 g of D_2O (598 mmol, 3.93 eq), and 4.09 g of K_2CO_3 (29.6 mmol, 0.20 eq). The steel vessel was sealed tightly with Teflon tape and brass fittings, and was placed into an oil bath which was then heated to 235 °C with stirring behind a blast shield. The vessel remained at 235 °C for 10 h, after which the vessel was left in the oil bath and cooled to room temperature. The material recovered was a dark brown liquid containing some black solid. The material was transferred to a round-bottom flask and furan was isolated by distillation at ~31 °C. Distillation yielded 6.66 g of a mixture of furan-d_x isotopologues. Analysis by millimeter-wave rotational spectroscopy confirmed the presence of all possible H/D isotopologues of furan (Scheme S1).

An initial attempt at deuteriation using the same reagents under standard reflux at atmospheric pressure for 24 h did not result in deuterium incorporation into furan.

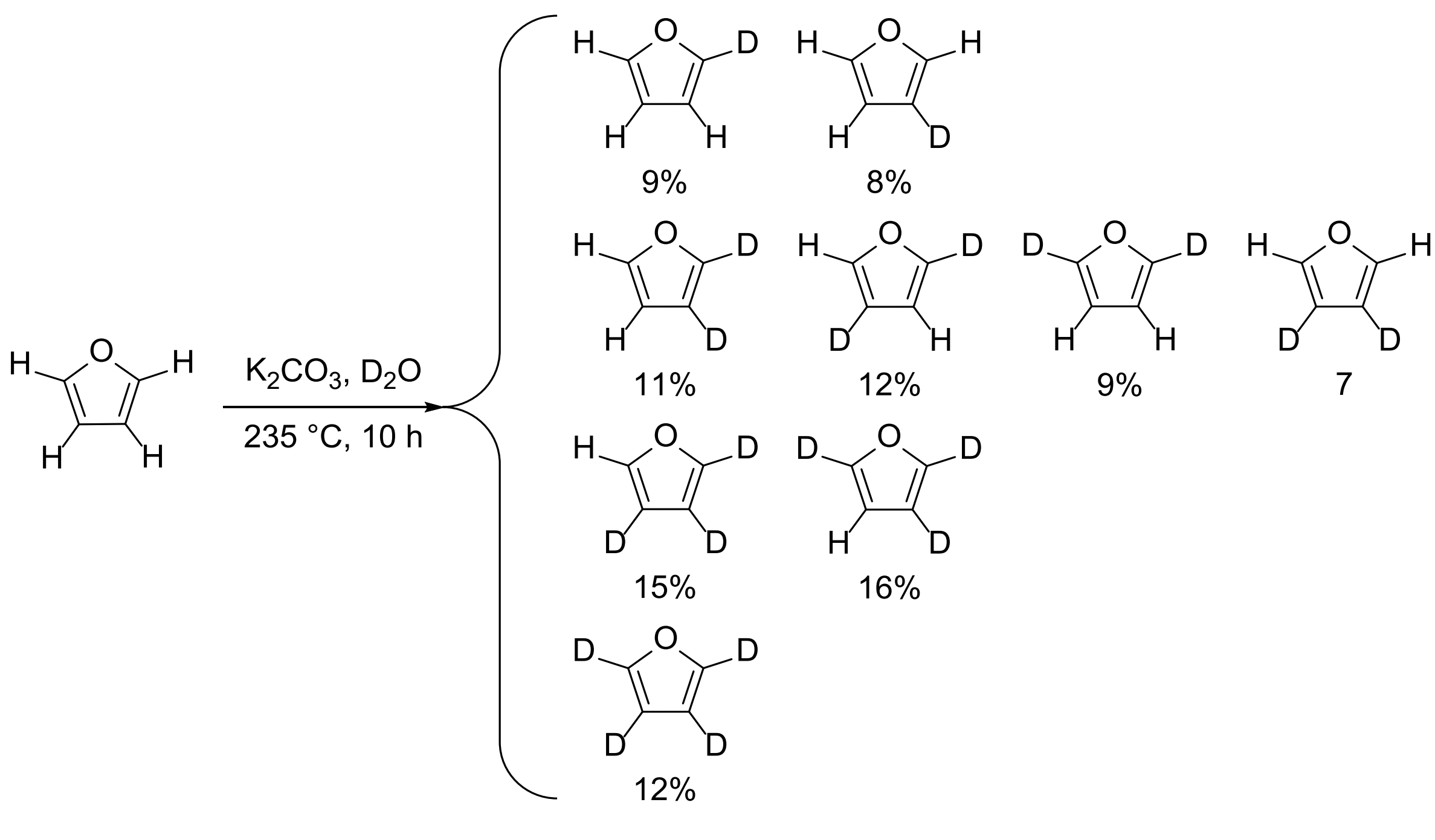

Scheme S1. Deuteriation of furan by carbonate-mediated exchange with D_2O under high-temperature, high-pressure conditions. Approximate percentages are estimated from relative intensities of transitions in the millimeter-wave rotational spectrum.

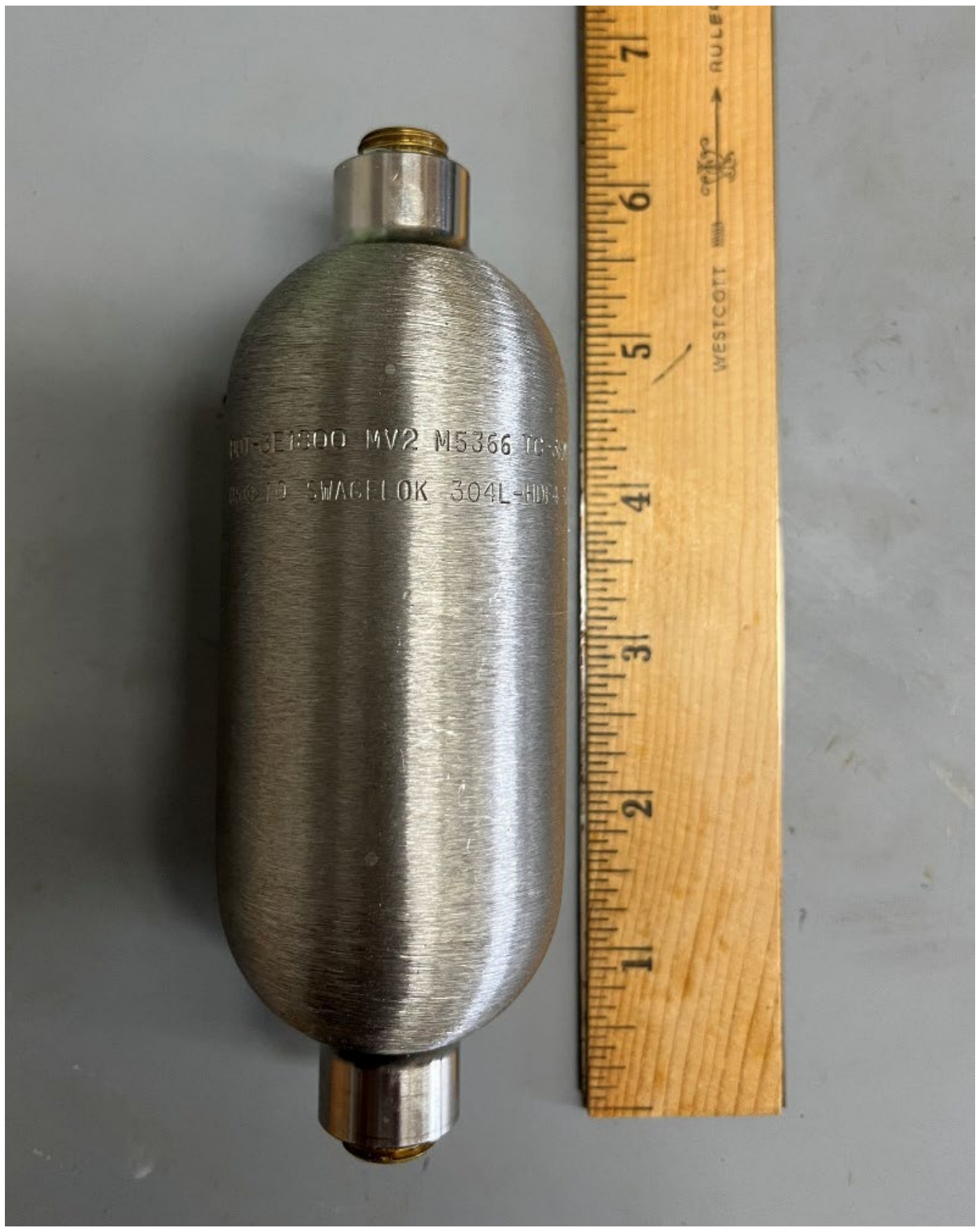

Figure S46. Photograph of vessel (with fittings) used for synthesis.

References

(1) Gordy, W.; Cook, R. *Microwave Molecular Spectra*; Wiley Interscience, 1984.

(2) Morgan, W. J.; Matthews, D. A.; Ringholm, M.; Agarwal, J.; Gong, J. Z.; Ruud, K.; Allen, W. D.; Stanton, J. F.; Schaefer, H. F., III. Geometric Energy Derivatives at the Complete Basis Set Limit: Application to the Equilibrium Structure and Molecular Force Field of Formaldehyde *J. Chem. Theory Comput.* **2018**, *14*, 1333−1350. DOI: 10.1021/acs.jctc.7b01138.

(3) Puzzarini, C.; Bloino, J.; Tasinato, N.; Barone, V. Accuracy and Interpretability: The Devil and the Holy Grail. New Routes across Old Boundaries in Computational Spectroscopy. *Chem. Rev.* **2019**, *119*, 8131–8191. DOI: 10.1021/acs.chemrev.9b00007.

(4) Feller, D. The use of systematic sequences of wave functions for estimating the complete basis set, full configuration interaction limit in water *J. Chem. Phys.* **1993**, *98*, 7059–7071. DOI: https://doi.org/10.1063/1.464749.

(5) Kutzelnigg, W.; Morgan, J. D., III. Rates of convergence of the partial-wave expansions of atomic correlation energies *J. Chem. Phys.* **1992**, *96*, 4484–4508. DOI: https://doi.org/10.1063/1.462811.

(6) Halkier, A.; Helgaker, T.; Jørgensen, P.; Klopper, W.; Koch, H.; Olsen, J.; Wilson, A. K. Basis-set convergence in correlated calculations on Ne, N_2, and H_2O. *Chem. Phys. Lett.* **1998**, *286*, 243–252.

(7) Halkier, A.; Helgaker, T.; Jørgensen, P.; Klopper, W.; Olsen, J. Basis-set convergence of the energy in molecular Hartree–Fock calculations *Chem. Phys. Lett.* **1999**, *302*, 437–446. DOI: https://doi.org/10.1016/S0009-2614(99)00197-9.

(8) Helgaker, T.; Jørgensen, P.; Olsen, J. *Molecular Electronic-Structure Theory*; John Wiley & Sons, 2000. DOI: https://doi.org/10.1002/9781119019572.ch13.

(9) Bomble, Y. J.; Stanton, J. F.; Kállay, M.; Gauss, J. Coupled-cluster methods including noniterative corrections for quadruple excitations. *J. Chem. Phys.* **2005**, *123*, 054101. DOI: 10.1063/1.1950567.

(10) Cheng, L.; Gauss, J. Analytic energy gradients for the spin-free exact two-component theory using an exact block diagonalization for the one-electron Dirac Hamiltonian. *J. Chem. Phys.* **2011**, *135*, 084114. DOI: 10.1063/1.3624397.

(11) Liu, W.; Peng, D. Exact two-component Hamiltonians revisited. *J. Chem. Phys.* **2009**, *131*, 031104. DOI: 10.1063/1.3159445.

(12) Dyall, K. G. Interfacing relativistic and nonrelativistic methods. II. Investigation of a low-order approximation. *J. Chem. Phys.* **1998**, *109*, 4201–4208. DOI: 10.1063/1.477026.

(13) In a previous investigation, we noted that the relativistic correction was insensitive to whether a re-contracted basis set was employed. In order to maintain consistency with our approach in the determination of the BTE in previous works, we have chosen to continue to employ the cc-pCVTZ basis set.

(14) Styers, W. H.; Esselman, B. J.; Wood, S. A.; Changala, P. B.; Jones, G. H.; McCarthy, M. C.; Stanton, J. F.; Woods, R. C.; McMahon, R. J. Cyclopropanone: Preparation, Rotational Spectroscopy, and Semi-Experimental Equilibrium (r_e^{SE}) Structure. *J. Am. Chem. Soc.* **2026**, *148*, 14072–14085. DOI: 10.1021/jacs.5c23052.

(15) Handy, N. C.; Yamaguchi, Y.; Schaefer, H. F. The diagonal correction to the Born–Oppenheimer approximation: Its effect on the singlet–triplet splitting of CH_2 and other molecular effects. *J. Chem. Phys.* **1986**, *84*, 4481–4484. DOI: 10.1063/1.450020.

(16) Born, M.; Huang, K. *Dynamical Theory of Crystal Lattices*; Oxford University Press, 1956.

(17) Gauss, J.; Puzzarini, C. Quantum-chemical calculation of Born-Oppenheimer breakdown parameters to rotational constants. *Mol. Phys.* **2010**, *108*, 269–277. DOI: https://doi.org/10.1080/00268970903433507.

(18) Puzzarini, C.; Gauss, J. Quantum-chemical determination of Born-Oppenheimer breakdown parameters for rotational constants: the open-shell species CN, CO^+ and BO *Mol. Phys.* **2013**, *111*, 2204–2210. DOI: https://doi.org/10.1080/00268976.2013.797614.

(19) Bak, B.; Christensen, D.; Dixon, W. B.; Hansen-Nygaard, L.; Rastrup Andersen, J.; Schottländer, M. The complete structure of furan. *J. Mol. Spectrosc.* **1962**, *9*, 124–129. DOI: 10.1016/0022-2852(62)90219-9.

(20) Tomasevich, G. R.; Tucker, K. D.; Thaddeus, P. Hyperfine structure of furan. *J. Chem. Phys.* **1973**, *59*, 131–135. DOI: 10.1063/1.1679780.

(21) Mata, F.; Martin, M. C.; Sørensen, G. O. Microwave spectra of deuterated furans. Revised molecular structure of furan. *J. Mol. Struct.* **1978**, *48*, 157–163. DOI: 10.1016/0022-2860(78)80018-0.

(22) Spycher, R. M.; Hausherr-Primo, L.; Grassi, G.; Bauder, A. Rotational spectra of isotopic furan-$(\text{argon})_n$, $n = 1,2$, complexes and their vibrationally averaged structures. *J. Mol. Struct.* **1995**, *351*, 7–17. DOI: 10.1016/0022-2860(94)08494-3.

(23) Barnum, T. J.; Lee, K. L. K.; McGuire, B. A. Chirped-Pulse Fourier Transform Millimeter-Wave Spectroscopy of Furan, Isotopologues, and Vibrational Excited States. *ACS Earth Space Chem.* **2021**, *5*, 2986–2994. DOI: 10.1021/acsearthspacechem.1c00182.

(24) Kisiel, Z. *PROSPE - Programs for ROtational SPEctroscopy*. http://info.ifpan.edu.pl/~kisiel/prospe.htm (accessed 28 April 2026).

(25) Demaison, J.; Császár, A. G.; Margulès, L. D.; Rudolph, H. D. Equilibrium Structures of Heterocyclic Molecules with Large Principal Axis Rotations upon Isotopic Substitution. *J. Phys. Chem. A* **2011**, *115*, 14078–14091. DOI: 10.1021/jp2063595.

(26) Townes, C. H.; Schawlow, A. L. *Microwave Spectroscopy*; Dover Publications, Inc., 1975.
